# Bioactive Bis(indole) Alkaloids from a *Spongosorites* sp. Sponge

**DOI:** 10.3390/md19010003

**Published:** 2020-12-23

**Authors:** Jae Sung Park, Eunji Cho, Ji-Yeon Hwang, Sung Chul Park, Beomkoo Chung, Oh-Seok Kwon, Chung J. Sim, Dong-Chan Oh, Ki-Bong Oh, Jongheon Shin

**Affiliations:** 1 Natural Products Research Institute, College of Pharmacy, Seoul National University, San 56-1, Sillim, Gwanak, Seoul 151-742, Korea; jaesung89@snu.ac.kr (J.S.P.); yahyah7@snu.ac.kr (J.-Y.H.); sungchulpark@snu.ac.kr (S.C.P.); ideally225@snu.ac.kr (O.-S.K.); dongchanoh@snu.ac.kr (D.-C.O.); 2 Department of Agricultural Biotechnology, College of Agriculture and Life Sciences, Seoul National University, Seoul 08826, Korea; eunji525@snu.ac.kr (E.C.); beomkoo01@snu.ac.kr (B.C.); 3 Department of Biological Sciences, College of Life Science and Nano Technology, Hannam University, 461-6 Jeonmin, Yuseong, Daejeon 305-811, Korea; cjsim@hnu.kr

**Keywords:** *Spongosorites* sp. sponge, bis(indole) alkaloids, spongosoritins, spongocarbamides, topsentin, sortase A inhibition

## Abstract

Six new bis(indole) alkaloids (**1**–**6**) along with eight known ones of the topsentin class were isolated from a *Spongosorites* sp. sponge of Korea. Based on the results of combined spectroscopic analyses, the structures of spongosoritins A–D (**1**–**4**) were determined to possess a 2-methoxy-1-imidazole-5-one core connecting the indole moieties, and these were linked by a linear urea bridge for spongocarbamides A (**5**) and B (**6**). The absolute configurations of spongosoritins were assigned by electronic circular dichroism (ECD) computation. The new compounds exhibited moderate inhibition against transpeptidase sortase A and weak inhibition against human pathogenic bacteria and A549 and K562 cancer cell lines.

## 1. Introduction

Alkaloids are widely recognized to play a central role in drug development [1,2,3]. These nitrogen-containing compounds have been in the limelight of medical chemistry and pharmacology research, with high structural complexity and various types of often potent bioactivity [1,2]. The level of structural complexity remarkably increases in those from the marine environment [4,5]. For example, among the most representative marine alkaloids are makaluvamines, manzamines, triptans, veranamines, coscinamides, and aplysinopsins from diverse colonial animals including sponges [2,6,7,8,9,10]. Bioactivity evaluations have shown that marine alkaloids and/or their synthetic analogs have various drug-related activities, including anti-tumoral, anti-malarial, anti-bacterial, and anti-viral, as well as enzymatic inhibition [2,4,5,11,12,13,14]. Overall, the importance of searching for novel bioactive marine alkaloids is constant.

Among the numerous sponge-derived alkaloids, topsentins are one of the most well-known classes; thus, extensive research has been conducted with respect to their structural determination, total synthesis, and bioactivity [15,16,17]. The diverse types of bioactivity reported include anti-inflammatory, anti-bacterial, anti-cancer, DNA-intercalating, and skin photoprotective, as well as the inhibition of Gram-positive bacterial enzyme sortase A, from our previous study [17,18,19,20,21]. These bis(indole) alkaloids, typically bearing an imidazole ring as the central core, were originally isolated from the sponge *Spongosorites genitrix*, which belongs to the family Halichondriidae [21,22]. Experiencing wide structural variations at both the imidazole moiety and the indole functionality, the succeeding analogs of topsentins include dragmacidins, hamacanthins, hytinadine A, nortopsentins, and rhopaladins from the genus *Spongosorites* and even a *Rhopalaea* sp. ascidian [23,24,25,26,27]. In a recent example, two new bis(indole) alkaloids, calcicamides A and B, bearing imidazole-cleaved 1,2-dicarbonyl moiety and exhibiting weak cytotoxicity against HeLa cells, were reported from the Irish sponge *Spongosorites calcicola* [28].

**Figure 1:**
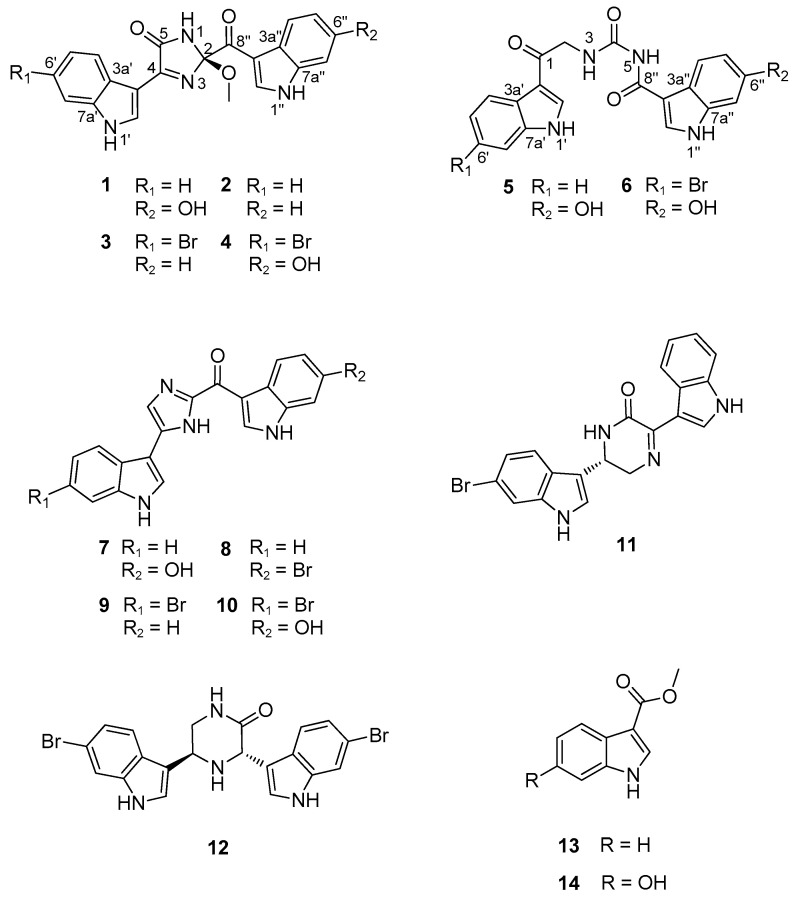
Chemical structures of compounds **1**–**14**.

Herein, as a result of the continuing search for sponge-derived bioactive compounds, we report the structures of six new bis(indole) alkaloids related to the topsentin family, along with eight known ones from a *Spongosorites* sp. sponge, collected from offshore of Jeju Island, Korea (Figure 1). The new compounds, spongosoritins A–D (**1**–**4**), possess the 2-methoxy-1-imidazole-5-one moiety as a common structural core with different auxiliary substituents at the indole moieties. The other new compounds, spongocarbamides A (**5**) and B (**6**), also have a linear urea-type linkage connecting the indole groups. Both the 2-methoxy-1-imidazole-5-one and linear urea moieties of the new compounds are unprecedented in topsentin alkaloids. The eight known compounds (**7**–**14**) consist of four topsentins, two hamacanthins, and two monoindoles. Consistent with our previous results, these indole-bearing compounds exhibited moderate inhibition of *Staphylococcus aureus*-derived sortase A (SrtA) and weak antibacterial activity against human pathogenic strains. In addition, several compounds showed weak to moderate cytotoxicity against A549 and K562 cancer cell lines.

## 2. Results and Discussion

### 2.1. Structure Elucidation

The molecular formula of spongosoritin A (**1**) was deduced by high resolution fast atom bombardment mass spectrometry (HRFABMS) analysis to be C_21_H_16_N_4_O_4_ with an unsaturation degree of 16: [M + H]^+^ *m/z* 389.1248 (calcd for C_21_H_17_N_4_O_4_, 389.1250). In accordance with the high unsaturation degree, the ^13^C NMR data of this compound presented most of the carbon signals at the aromatic/olefinic zone (δ_C_ *ca* 150–100). The corresponding HSQC-based proton signals were also observed at the deshielded region (δ_H_ 8.67–6.70) in the ^1^H NMR data, indicating a polyaromatic nature for **1** (Table 1). Further examination of these data also revealed the presence of three exchangeable protons at the far deshielded region, δ_H_ 12.00, 11.80, and 10.10. Thus, **1** was thought to be an alkaloid bearing indoles or similar aromatic moieties. 

**Figure 2:**
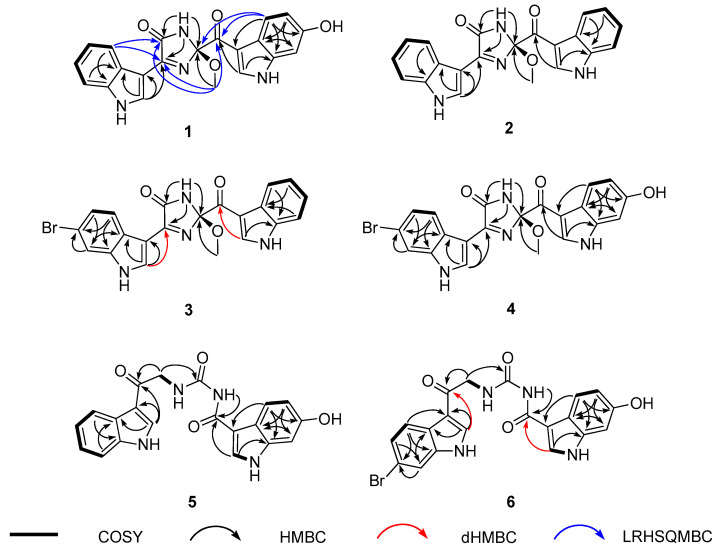
Key correlations of ^1^H-^1^H COSY (bold line), HMBC (arrow), dHMBC (red arrow), and LRHSQMBC (blue arrow) experiments for compounds **1**–**6**.

The preliminary spectroscopic interpretation was further investigated by combined ^1^H-^1^H COSY and HMBC experiments. First, the COSY data revealed a linear array of four aromatic protons, H-4′–H-7′ (δ_H_ 8.18, 7.16, 7.23, and 7.51, respectively) constructing an *ortho*-disubstituted benzene. Then the HMBC correlations of these and an isolated H-2′ methine proton (δ_H_ 8.67) with neighboring carbons readily deduced an indole moiety (N-1′ and C-2′-C-7a′) (Figure 2). Subsequently, a dHMBC correlation with H-2′ attached a nonprotonated carbon (C-4, δ_C_ 160.1) at C-3′ (δ_C_ 105.9) of this indole, whose functionality was clarified later. Another indole moiety (N-1′′ and C-2′′–C-8′′) was also constructed by the COSY-based ABX-type spin-coupling pattern of three protons, H-4′′ (δ_H_ 7.92), H-5′′ (δ_H_ 6.70), and H-7′′ (δ_H_ 6.87), as well as the HMBC correlations of these and an isolated H-2′′ (δ_H_ 8.55) with aromatic carbons. A hydroxy group was attached at C-6′′ (δ_C_ 154.2) from the significantly deshielded chemical shift of this carbon. Similarly, a ketone was placed at C-8′′ (δ_C_ 186.2) from its chemical shift as well as a three-bond dHMBC correlation with H-2′′.

The 2D NMR interpretation of **1** remained a partial structure with the formula C_4_H_4_N_2_O_2_ bearing an unsaturation degree of 3 and connecting two indole moieties. A methoxy group (δ_C_/δ_H_ 49.3/3.28) was readily evidenced by the HSQC data. Then the other elements were thought to form a cyclic moiety with two double bonds from the chemical shifts of three non-protonated carbons at δ_C_ 166.4, 160.1, and 106.9. This was fulfilled by sequential interpretation of the HMBC data, and then extended by the long-range heteronuclear single quantum multiple bond correlation (LRHSQMBC) data for long-range couplings (Figure 2) [29]. First, the methoxy protons (2-OCH_3_) showed a three-bond HMBC correlation with C-2 (δ_C_ 106.9). This oxygenated carbon was directly attached at C-8′′ by LRHSQMBC couplings at H-4′′/C-2 and C-8′′ and 2-OCH_3_/C-8′′.

Meanwhile the exchangeable NH-1 proton (δ_H_ 10.1) was linked with all three carbons at δ_C_ 166.4, 160.1, and 106.9 (C-2) by the HMBC data. Of these, the carbon at δ_C_ 160.1 was preliminarily found to be connected at C-3′ of the C-2′ indole moiety. Subsequently, the LRHSQMBC-based extended correlations of the former two carbons with H-5′ placed these at C-5 and C-4, respectively. Their deshielded chemical shifts, in conjunction with the molecular formula and unsaturation degree, defined them as an amide carbonyl (C-5) and an imine (C-4) with *sp*^2^ nitrogen, together forming a cyclic moiety. This was confirmed by the LRHSQMBC data, in which both carbons showed long-range correlations with the 2-OCH_3_ protons, constructing a 2-methoxy-1-imidazole-5-one moiety (Figure 2). Alternatively, the switch of functionality between C-4 and C-5 formed a four-member cyclic diazete moiety (N = C–N) consisting of C-2, C-5, and two nitrogen atoms, while the C-4 carbon was a ketone. This interpretation was readily erased by a far shielded chemical shift of C-4 (δ_C_ 160.1) as a ketone. Thus, the structure of spongosoritin A (**1**) was determined to be an imidazolone-bearing bis(indole) alkaloid related to the topsentin class. The presence of imidazolone core is unprecedented among the topsentins, to the best of our knowledge. The absolute configuration at the C-2 stereogenic center was discussed later with congeners.

The ^13^C and ^1^H NMR data of spongosoritin B-D (**2**–**4**) were very similar to those of **1** at the imidazolone moiety (Table 1). Therefore, the structural difference must occur at the substitutions at the indole moieties, as commonly found in the topsentin alkaloids. Bearing this in mind, the molecular formula of **2** was analyzed for C_21_H_16_N_4_O_3_ by HRFABMS data: [M + H]^+^ *m/z* 373.1303 (calcd for C_21_H_17_N_4_O_3_, 373.1301). The combined 1D and 2D NMR data readily revealed the presence of a fully protonated indole replacing the hydroxy-bearing one (N-1′′and C-2′′-C-7a′′) in **1** (Figure 2), defining spongosoritin B (**2**) as a dehydroxy derivative of spongosoritin A (**1**). 

Spongosoritin C (**3**) exhibited a 1:1 peak ratio of [M]^+^ and [M + 2]^+^ in low-resolution electrospray ionization mass spectrometry (LRESIMS) analysis, which is a characteristic pattern attributed to the presence of a bromine. Indeed, the molecular formula of **3** was established to be C_21_H_15_BrN_4_O_3_ by the HRFABMS data: [M + H]^+^ *m/z* 451.0413 (calcd for C_21_H_16_Br^79^N_4_O_3_, 451.0406). The position of bromine on an indole group was verified at C-6′ (δ_C_ 115.7) by its shielded carbon chemical shift and by the HMBC correlations with the neighboring H-4′ (δ_H_ 8.17), H-5′ (δ_H_ 7.30), and H-7′ (δ_H_ 7.72). Then this bromoindole moiety was directly connected to C-4 of the imidazolone ring by the crucial HMBC correlation at H-2′ (δ_H_ 8.69)/ C-4 (δ_C_ 161.1) (Figure 2). Based on the same 2D NMR analysis, the other indole moiety was found to be a fully protonated one, and was assigned as a brominated derivative of **2**.

The molecular formula of spongosoritin D (**4**) was analyzed for C_21_H_15_BrN_4_O_4_ by HRFABMS analysis: [M + H]^+^ *m/z* 467.0351 (calcd for C_21_H_16_Br^79^N_4_O_4_, 467.0355). As indicated by the MS and ^13^C and ^1^H NMR data (Table 1), this compound was anticipated to possess one bromoindole and one hydroxyindole. This interpretation was confirmed by combined 2D NMR analysis, in which these substituents were first secured at C-6′ (δ_C_ 115.1) and C-6′′ (δ_C_ 154.3) by their HMBC correlation with neighboring ring protons. Subsequently, the indole units were connected to the imidazolone core by diagnostic 3-bond dHMBC correlations at H-2′ (δ_H_ 8.67)/C-4 (δ_C_ 160.9) and H-2′′ (δ_H_ 8.53)/C-8′′ (δ_C_ 185.9), respectively. Thus, the structure of spongosoritin D (**4**) showed that it was a member of the spongosoritin class possessing two substituted indoles.

**Figure 3:**
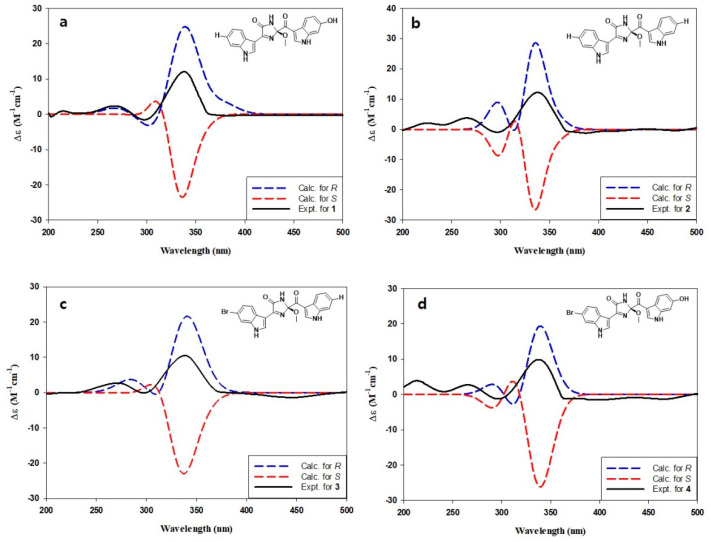
(**a**–**d**) Comparison of measured and calculated ECD profiles of compounds **1**–**4**, respectively.

Compounds **1**–**4** possessed a common stereogenic center at the methoxy-bearing C-2 of the imidazolone moiety. In order to determine the absolute configuration at this position, a density functional theory (DFT)-based computational method was carried out. That is, the initial approach was to measure the specific rotations for **1**–**4** to verify the same absolute configuration at the C-2 center. However, the obtained rotations were severely irregular and resulted in alternating signs, possibly due to the presence of significantly different conformers (Supplementary Figures S40–S43) [30,31]. Notable examples of this phenomenon among marine natural products would be the sponge-derived discorhabdins, fungus-derived herqueinones, and ascidian-derived isocadiolides [32,33,34,35,36]. This problem was solved by the ECD measurements. Unlike the optical rotations measured at the fixed wavelength of 589 nm, the ECD profiles of **1**–**4** were stable in the range of 200–400 nm and showed very similar profiles to each other (Figure 3) [32]. Subsequently, the measured data were compared with the calculated ECD profiles, thus assigning the 2*R* configuration for spongosoritins A–D.

In addition to **1**–**4**, two structurally related but remarkably distinct congeners were isolated. The molecular formula of spongocarbamide A (**5**) was deduced to be C_20_H_16_N_4_O_4_ by HRFABMS data: [M + H]^+^ *m/z* 377.1249 (calcd for C_20_H_17_N_4_O_4_, 377.1250). The ^13^C and ^1^H NMR data of this compound were highly reminiscent of those of **1**–**4**, indicating the presence of two indole moieties (Table 2). Based on the results of combined 2D NMR analyses, these were identified to be each one of fully protonated and hydroxy-bearing indole as **1** (Figure 2). The remaining signals in the NMR data were three non-protonated carbons (δ_C_ 189.7, 165.5 and 154.5), one methylene (δ_C_/δ_H_ 46.5/4.71 (2H)), and two exchangeable NH protons (δ_H_ 10.20 and 9.35). Of the non-protonated carbons, two carbonyls were secured at C-1 (δ_C_ 189.7) and C-8′′ (δ_C_ 165.5) by their diagnostic 3-bond HMBC correlation with H-2′ (δ_H_ 8.47) and H-2′′ (δ_H_ 8.34), respectively.

The COSY data showed a spin correlation between NH (δ_H_ 9.35) and methylene protons, which were directly connected to each other, forming an amino-methylene group by the vicinal coupling constant (*J* = 5.2 Hz). This group was placed between the C-1 carbonyl and another nonprotonated carbon (δ_C_ 154.5) by their HMBC correlation with the methylene protons. Based on the chemical shift of C-1 carbonyl (δ_C_ 189.7), the amino-methylene group was unambiguously designated as 3-NH and 2-CH_2_, leaving the other carbon at C-4. Additionally, the HMBC data showed a long-range correlation between an NH (δ_H_ 10.20) and C-8′′, assigning this amide nitrogen at 5-NH and functionalizing the C-4 carbon as a urea. Although it was not directly proven by either 2D NMR or MS fragmentation analysis, the chemical shift of C-4 (δ_C_ 154.5) was decisive for the assignment of the urea group. Thus, the structure of spongocarbamide A (**5**) was determined to be a linear bis(indole) alkaloid bearing a urea group.

The molecular formula of spongocarbamide B (**6**) was deduced to be C_20_H_15_BrN_4_O_4_ by HRFABMS analysis: [M + H]^+^ *m/z* 455.0356 (calcd for C_20_H_16_Br^79^N_4_O_4_, 455.0355). The spectroscopic data of this compound were similar to those of **5**, revealing the same urea and linear portion between them (Table 2). Therefore, the structural difference was anticipated to exist at the bis(indole) moieties. As for the topsentin congeners, the indole groups were identified by combined 1D and 2D NMR analyses followed by their HMBC- and dHMBC-based connectivity with the linear portion (Figure 2). Conclusively, the bromoindole and hydroxyindole were placed at C-2′ and C-2′′, respectively, identical to **4**, defining spongocarbamide B (**6**) as a brominated derivative of spongocarbamide A (**5**).

In addition to new compounds **1**–**6**, the crude extract of *Spongosorites* sp. contained several indole-bearing congeners. Extensive chromatographic separation of these followed by combined spectroscopic analyses led to the identification of eight previously reported compounds. Categorized as four topsentins, two hamacanthins, and two monoindoles, these were topsentin (**7**) [37], isobromodeoxytopsentin (**8**) [14], bromodeoxytopsentin (**9**) [14], bromotopsentin (**10**) [14], (*R)*-6′′-debromohamacanthin A (**11**) [38], (3*S*, 5*R*)-6-dibromo-*trans*-3,4-dihydrohamacanthin B (**12**) [39], 3-carbomethoxyindole (**13**) [40], and 6-hydroxy-3-carbomethoxyindole (**14**) [40]. The spectroscopic data of these were in good accordance with those in the literature.

New compounds **1**–**6** from *Spongosorites* sp. showed remarkable structural similarity with topsentins by the presence of not only bis(indole) moieties but also two nitrogen atoms associated with carbonyl group(s). The diversity of indole functionalities and the unanimously presented C-8′′ keto group are further supportive of the structural relation. On the contrary, the new compounds possessed either imidazolone core (**1**–**4**) or linear urea linkage (**5** and **6**), which is unprecedented among topsentin alkaloids. Regarding this, the high oxidant state of the new compounds raises the possibility that these are artifacts derived from topsentins or structurally related precursors during the storage or isolation process. Despite the significant differences in both chemical structure and reaction type, dye-sensitized photooxidation of mono- and bis-phenylimidazoles to corresponding imidazolones would increase the possibility that the new compounds are artifacts derived from natural alkaloids [41]. However, the significant structural deviations of new compounds from topsentins prevented us from postulating plausible mechanisms for either their biotic or abiotic production. Although their origin remains unclear, the structural novelty of these new compounds and their bioactivity, described later, contribute to the value of sponge-derived topsentin alkaloids.

### 2.2. Biological Activity

In our previous studies, topsentin alkaloids showed antibacterial and cytotoxic activities and SrtA inhibition [17,19]. The same bioassays were pursued with the new compounds and the known congeners, with a particular emphasis on SrtA inhibition. The rationale of SrtA inhibition is that this transpeptidase decorates the surfaces of Gram-positive bacteria with a diverse array of proteins that enable microbes to effectively interact with their environment, and it is not required for bacterial growth or viability [42,43]. It is thus considered a promising target for the development of anti-virulence drugs aimed at interfering with important virulence mechanisms such as adhesion to host tissues. To assess the SrtA inhibitory activity of compounds **1**–**14**, recombinant SrtA derived from *S*. *aureus* ATCC6538p was purified from *Escherichia coli* extracts using metal chelate-affinity chromatography [44]. The enzyme activity was determined from the fluorescence intensity upon cleavage of a peptide substrate containing the LPETG motif [45,46]. The inhibitory potency of the isolated compounds against recombinant SrtA, expressed as IC_50_ values, is shown in Table 1 and was compared to that of known SrtA inhibitors berberine chloride (IC_50_ = 87.4 μM) and triphasiol (IC_50_ = 37.9 μM) [47]. Compounds **2**, **3**, **5**, **6**, and **8**–**11** displayed moderately inhibited SrtA activity (IC_50_ values of 79.4–34.0 μM). Among them, bromodeoxytopsentin (**9**) exhibited the most potent inhibitory activity (IC_50_ = 34.0 μM). The noticeably weak inhibition of monoindoles (**13** and **14**) emphasizes the role of bis(indole) in SrtA activity inhibition (Table 3).

The antimicrobial activity of compounds **1–14** was also evaluated against phylogenetically diverse pathogenic bacterial and fungal strains [48]. Among these compounds, compounds **7** and **10–12** displayed moderate to significant antibacterial activity (minimum inhibitory concentration (MIC) values of 64–8 μg/mL) against Gram-positive *Staphylococcus aureus* strain Newman, *Enterococcus faecalis* ATCC19433, and *Enterococcus faecium* ATCC19434. Compounds **7** and **10**–**12** also showed inhibitory activity against Gram-negative *Klebsiella pneumoniae* ATCC10031 (MIC = 64–8 μg/mL) and *Salmonella enterica* ATCC14028 (MIC = 64–8 μg/mL), but not *Escherichia coli* ATCC25922 (MIC > 128 μg/mL), using ampicillin and tetracycline as positive control compounds. However, new compounds **1–6** showed far weaker antibacterial activity than topsentin congeners. The antifungal activity of compounds **1–14** was also evaluated against pathogenic fungal strains *Candida albicans* ATCC10231, *Aspergillus fumigatus* HIC6094, *Trichophyton rubrum* NBRC9185, and *Trichophyton mentagrophytes* IFM40996 using amphotericin B as a positive control compound. However, none of these compounds exhibited inhibitory activity (MIC > 128 μg/mL) against the tested strains. In addition to cytotoxicity, several compounds showed weak inhibition against A549 and K562 cancer cell lines. Consistent with the antibacterial activity, the cytotoxicity of new compounds **1–6** was remarkably weaker than that of topsentins **7–9** and **12,** suggesting the contribution of imidazole or a similar ring moiety to the bioactivity.

## 3. Materials and Methods

### 3.1. General Experimental Procedures

Optical rotations were measured using a JASCO P-1020 polarimeter (JASCO Inc., Easton, MD, USA) with a 1 cm cell. UV spectra were recorded by a Hitachi U-3010 spectrophotometer (Hitachi, Fukuoka, Japan). CD spectra were obtained with an Applied Photophysics Chirascan Plus CD spectrometer (Applied Photophysics, Leatherhead, Surrey, UK) using a 0.2 mm cell. IR spectra were acquired on a JASCO 4200 FT-IR spectrometer (JASCO Inc., Easton, MD, USA) using a ZnSe cell. High-resolution FABMS spectrometric data were obtained using a Jeol JMS 700 mass spectrometer (JEOL Ltd., Tokyo, Japan) with *meta*-nitrobenzyl alcohol (NBA) as a matrix at the National Center for Inter-University Research Facilities (Seoul, Korea). NMR spectra were recorded using Bruker Avance 600 and 800 MHz (Bruker, Billerica, MA, USA) in DMSO-*d*_6_ with solvent peaks at δ_H_ 2.50/δ_c_ 39.50 as the internal standards. The dHMBC NMR experiments were performed on a Bruker Avance 800 MHz utilizing the standard Bruker pulse sequence code (hmqcgpgf) with data points of 2048 × 570 and optimized for *J*_CH_ measurement at 2Hz. The LRHSQMBC NMR experiment was measured on a Bruker Avance 800 MHz using the same pulse program described in a previous study, with data points of 2048 × 620 and optimized for *J*_CH_ measurement at 2 Hz [28]. HPLC was performed using a SpectraSYSTEM p2000 (Thermo Fisher Scientific, Waltham, MA, USA) equipped with a refractive index detector (SpectraSYSTEM RI-150 (Thermo Fisher Scientific, Waltham, MA, USA) and a UV-Vis detector (Gilson UV-Vis-151 (Gilson Inc., Middleton, WI, USA). All solvents were of spectroscopy grade or distilled prior to use. 

### 3.2. Animal Material

A specimen of *Spongosorites sp.* sponge (sample number 14J-17) was collected by hand using SCUBA offshore of Seogwipo, Jeju Island, Korea, at a depth of 25–30 m on 1 October 2014. The specimen was a small piece (5.5 × 3.5 × 1.5 cm). The surface was smooth and covered with other sponge. Oscules were 0.5–1.5 cm in diameter. The texture was firm and compressible. The live color was yellowish brown, turning to dark brown after collection. The skeleton was composed of small and large oxeas (70–200 × 3–10 μm, 900–1200 × 30–40 μm), styles (rare, 760–1100 × 18–36 μm), and strongyles (rare, 420 × 1020 × 24–42 μm). A voucher specimen (registry no. Spo. 83) is deposited in the Natural History Museum, Hannam University, Daejeon, Korea. 

### 3.3. Extraction and Isolation

The sponge specimen was immediately frozen at −25 °C until use. Lyophilized and macerated specimens (120 g) were repeatedly extracted with MeOH (3 × 2 L) and CH_2_Cl_2_ (3 × 2 L). The combined yellow extracts (29.8 g) were dried in vacuo and partitioned between H_2_O (18.0 g) and *n*-BuOH (10.7 g), then the latter layer was repartitioned between H_2_O-MeOH (15:85, 7.1 g) and *n*-hexane (2.6 g). The H_2_O-MeOH layer was separated by vacuum flash chromatography with ODS resin using sequential mixtures of MeOH and H_2_O as the eluents (6 fractions in H_2_O-MeOH gradient, from 50:50 to 0:100), followed by acetone and finally EtOAc. Based on the aromatic proton signals observed in ^1^H-NMR analysis, 3 fractions were chosen for HPLC separation: 40:60 H_2_O-MeOH (0.51 g), 30:70 H_2_O-MeOH (1.08 g), and 20:80 H_2_O-MeOH (0.40 g).

The first fraction, 50:50 H_2_O-MeOH, was separated by semi-preparative reversed-phase HPLC (YMC-ODS column, 10 × 250 mm; H_2_O-MeOH (70:30); 2.0 mL/min) to yield **14** (*t*_R_ = 60.1 min). The 40:60 H_2_O-MeOH fraction was separated by semi-preparative HPLC (H_2_O-MeOH (54:46); 2.0 mL/min) to yield 4 peaks at *t*_R_ = 35.1, 46.8, 62.4, and 66.3 min. The first peak was purified by reversed-phase analytical HPLC (YMC-ODS column, 4.6 × 250 mm; H_2_O-MeCN (70:30); 0.7 mL/min) to afford **1** (*t*_R_ = 26.8 min). The third peak obtained at *t*_R_ = 62.4 min was purified by similar HPLC conditions (H_2_O-MeCN (72:28); 0.7 mL/min) to afford **5** (*t*_R_ = 34.1 min). Compounds **13** and **7** from the original HPLC peaks at *t*_R_ = 46.8 and 66.3 min, respectively, were pure by ^1^H NMR analysis, so were not purified further.

The 30:70 H_2_O-MeOH fraction from vacuum flash chromatography was separated by semi-preparative HPLC (H_2_O-MeOH (43:57); 2.0 mL/min) to afford 4 peaks at *t*_R_ = 34.7, 46.2, 52.9, and 60.1 min. The first peak was separated by analytical HPLC (H_2_O-MeCN gradient (from 75:25 to 60:40 in 80 min); 0.7 mL/min) to yield compounds **4** (*t*_R_ = 72.0 min) and **2** (*t*_R_ = 78.3 min). The third peak at *t*_R_ = 52.9 min was purified by the same gradient HPLC condition to afford **6** (*t*_R_ = 54.1 min). Compounds **8** and **10** from the original HPLC peaks at *t*_R_ = 46.2 and 60.1 min, respectively, were pure by ^1^H NMR analysis, so were not purified further. Finally, the 20:80 H_2_O-MeOH fraction was separated by semi-preparative HPLC (H_2_O-MeOH, (36:64); 2.0 mL/min) to yield 4 peaks at *t*_R_ = 48.7, 52.6, 54.1, and 60.3 min. The peak at *t*_R_ = 54.1 min was then purified by analytical HPLC (0.7 mL/min; H_2_O-MeCN (62:38)) to afford **3** (*t*_R_ = 54.7 min). The other peaks obtained at *t*_R_ = 48.7, 52.6, and 60.3 min were determined to be **9**, **11**, and **12**, respectively. Overall, the amounts of isolated materials were 7.8, 2.1, 1.1, 4.8, 0.8, 0.7, 28.1, 33.1, 2.3, 1.8, 2.8, 2.1, 1.8, and 1.4 mg for **1**–**14**, respectively.

*Spongosoritin A* (**1**)**.** yellow, amorphous solid; ${\left[\alpha \right]}_{D}^{\mathrm{25}}$ ± 40 (*c* 1.0, MeOH); UV (MeOH) λ_max_ (log*ε*) 280 (3.40), 330 (3.31) nm; ECD (*c* 1.3 × 10^−3^ M, MeOH) λ_max_ (Δε) 338 (+12.0), 298 (−1.6) nm; IR (ZnSe) *ν*_max_ 3289, 2353, 1717, 1599, 1516, 1431 cm^−1^; ^1^H and ^13^C NMR data, see Table 1; HRFABMS *m/z* 389.1248 [M + H]^+^ (calcd for C_21_H_17_N_4_O_4_, 389.1250).

*Spongosoritin B* (**2**)**.** yellow, amorphous solid; ${\left[\alpha \right]}_{D}^{\mathrm{25}}$ ± 7 (*c* 0.8, MeOH); UV (MeOH) λ_max_ (log*ε*) 280 (3.15), 330 (3.09) nm; ECD (*c* 1.1 × 10^−3^ M, MeOH) λ_max_ (Δε) 336 (+12.2), 296 (−0.9) nm; IR (ZnSe) *ν*_max_ 3278, 2354, 1718, 1598, 1516 cm^−1^; ^1^H and ^13^C NMR data, see Table 1; HRFABMS *m/z* 373.1303 [M + H]^+^ (calcd for C_21_H_17_N_4_O_3_, 373.1301).

*Spongosoritin C* (**3**)**.** yellow, amorphous solid; ${\left[\alpha \right]}_{D}^{\mathrm{25}}$ ± 10 (*c* 0.8, MeOH); UV (MeOH) λ_max_ (log*ε*) 280 (3.36), 330 (3.33) nm; ECD (*c* 1.0 × 10^−3^ M, MeOH) λ_max_ (Δε) 343 (+12.0), 300 (−1.5) nm; IR (ZnSe) *ν*_max_ 3312, 2354, 1712, 1599, 1515, 1425 cm^−1^; ^1^H and ^13^C NMR data, see Table 1; HRFABMS *m/z* 451.0413 [M + H]^+^ (calcd for C_21_H_16_Br^79^N_4_O_3_, 451.0406).

*Spongosoritin D* (**4**)**.** yellow, amorphous solid; ${\left[\alpha \right]}_{D}^{\mathrm{25}}$ ± 20 (*c* 1.0, MeOH); UV (MeOH) λ_max_ (logε) 280 (3.50), 330 (3.40) nm; ECD (*c* 0.8 × 10^−3^ M, MeOH) λ_max_ (Δε) 338 (+9.9), 296 (−1.3) nm; IR (ZnSe) *ν*_max_ 3278, 2354, 1750, 1711, 1600, 1515, 1424 cm^−1^; ^1^H and ^13^C NMR data, see Table 1; HRFABMS *m/z* 467.0351 [M + H]^+^ (calcd for C_21_H_16_Br^79^N_4_O_4_, 467.0355).

*Spongocarbamide A* (**5**)**.** yellow, amorphous solid; UV (MeOH) λ_max_ (log*ε*) 290 (3.34) nm; IR (ZnSe) *ν*_max_ 3278, 2354, 1684, 1640 cm^−1^; ^1^H and ^13^C NMR data, see Table 2; HRFABMS *m/z* 377.1249 [M + H]^+^ (calcd for C_20_H_17_N_4_O_4_, 377.1250).

*Spongocarbamide B* (**6**)**.** yellow, amorphous solid; UV (MeOH) λ_max_ (log*ε*) 280 (3.34) nm; IR (ZnSe) *ν*_max_ 3290, 2354, 1684, 1641, 1516, 1444 cm^−1^; ^1^H and ^13^C NMR data, see Table 2; HRFABMS *m/z* 455.0356 [M + H]^+^ (calcd for C_20_H_16_Br^79^N_4_O_4_, 455.0355).

### 3.4. ECD Calculations

Based on the density functional theory (DFT) calculations, the geometries were optimized to the ground-state level by Turbomole computer software. The basis parameter sets used were def-SVP and the B3-LYP functional for all atoms. The stabilized ground state of each model compound was further confirmed through the harmonic frequency calculation. The calculated ECD data were derived from the optimized structures obtained with TD-DFT at the B3-LYP functional. The ECD spectra were simulated by overlapping Gaussian functions for each transition, where *σ* is the width of the band at height 1/*e*. Values Δ*E_i_* and *R_i_* are the excitation energy and rotatory strength for transition *i*, respectively. In the current work, the value of *σ* was 0.10 eV.

$$\Delta \varepsilon \left(E\right)=\;\frac{1}{2.297\;\times 10^{-39}}\;\frac{1}{\sqrt{2\pi \sigma }}\overset{A}{\underset{i}{\sum }}\Delta E_{i}R_{i}e^{\left[-{\left(E-\Delta E_{i}\right)}^{2}/{\left(2\sigma \right)}^{2}\right]}$$

### 3.5. SrtA Inhibition Assay

Before evaluating the SrtA inhibitory activity of the test compounds, recombinant SrtA from *S*. *aureus* ATCC6538p was prepared according to methods described in a previous paper [44]. The SrtA inhibitory activity of the test compounds was determined according to previously described methods [45,46] with minor modifications. The reaction was carried out with 100 μL buffer (50 mM Tris-HCl, 5 mM CaCl_2_, and 150 mM NaCl, pH 7.5), 7.5 µM synthetic peptide dabcyl-LPETG-edans (AnaSpec, Inc., Fremont, CA, USA), 7.5 µM purified SrtA, and test samples at various concentrations. Each sample was dissolved in dimethyl sulfoxide (DMSO) and diluted with reaction buffer to obtain a final concentration of 1% DMSO, which did not influence enzyme activity. The SrtA inhibition assay was conducted at 37 °C for 1 h, and inhibition was quantified fluorometrically using a microplate reader (FLx800, BioTek Instruments, Winooski, VT, USA) at excitation and emission wavelengths of 350 and 495 nm, respectively. Berberine chloride and triphasiol [47] were used as reference inhibitors of SrtA.

### 3.6. Antibacterial, Antifungal, and Cytotoxic Assays

The antibacterial activity of compounds **1**–**14** was evaluated against Gram-positive bacteria (*S*. *aureus* strain Newman, *E*. *faecalis* ATCC19433, and *E*. *faecium* ATCC19434) and Gram-negative bacteria (*K*. *pneumoniae* ATCC10031, *S*. *enterica* ATCC14028, and *E*. *coli* ATCC25922) using a previously reported method [48]. For antifungal activity, *C*. *albicans* ATCC10231, *A*. *fumigatus* HIC6094, *T*. *mentagrophytes* IFM40996, and *T*. *rubrum* NBRC9185 strains were used to measure the inhibition of compounds **1**–**14** by following previously reported procedures [48]. An MTT assay was performed as previously described to determine the cytotoxicity against A549 and K562 cell lines [19]. These cancer cell lines were purchased from the Korean Cell Line Bank (KCLB), Seoul, Republic of Korea.

## 4. Conclusions

Six new bis(indole) alkaloids along with eight known ones of the topsentin class were isolated from a *Spongosorites* sp. sponge collected from Jeju Island, Korea. Based on the results of combined spectroscopic analyses, new compounds spongosoritin A–D (**1**–**4**) were defined to possess 2-methoxy-1-imidazole-5-one as a common structural core, while spongocarbamides A (**5**) and B (**6)** had a linear urea-type linkage connecting the indole groups. Both the 2-methoxy-1-imidazole-5-one and linear urea moieties of the new compounds were unprecedented among the topsentins and related indole alkaloids. These indole-bearing compounds exhibited moderate inhibition of sortase A and weak antibacterial activity against human pathogenic strains. In addition, several compounds were weakly cytotoxic against A549 and K562 cancer cell lines. Thus, the new compounds could contribute significantly to both the structural diversity and bioactivity of sponge-derived topsentin alkaloids.

## Figures and Tables

**Table 1:** ^13^C and ^1^H NMR assignments for compounds **1**–**4***^a^*.

No.	1 *^b^*		2 ^*b*^		3 ^*b*^		4 ^*b*^	
	ppm, Type	δ, mult (*J* in Hz)	ppm, Type	δ, mult (*J* in Hz)	ppm, Type	δ, mult (*J* in Hz)	ppm, Type	δ, mult (*J* in Hz)
1		10.10, s		10.20, s		10.20, s		10.10, s
2	106.9, C		106.9, C		107.0, C		107.0, C	
4	160.1, C		161.1, C		161.1, C		160.9, C	
5	166.4, C		166.4, C		166.1, C		166.2, C	
1′		12.00, s		ND *^c^*		12.10, s		11.90, s
2′	132.9, CH	8.67, s	133.0, CH	8.68, s	133.9, CH	8.69, d (3.0)	132.9, CH	8.67, s
3′	105.9, C		105.9, C		106.0, C		106.0, C	
3a′	125.6, C		125.6, C		124.6, C		124.4, C	
4′	121.5, CH	8.18, d (7.8)	121.5, CH	8.18, d (7.8)	123.1, CH	8.17, d (7.8)	123.1, CH	8.09, d (8.2)
5′	121.5, CH	7.16, dd (8.0, 7.8)	121.6, CH	7.16, dd (7.8, 7.0)	124.4, CH	7.30, dd (7.8, 1.5)	124.6, CH	7.30, dd (8.2, 1.9)
6′	123.1, CH	7.23, dd (8.0, 7.4)	123.1, CH	7.23, dd (8.0, 7.0)	115.7, C		115.1, C	
7′	112.2, CH	7.51, d (8.0)	112.4, CH	7.51, d (8.0)	115.1, CH	7.72, d (1.5)	115.7, CH	7.72, d (1.9)
7a′	136.4, C		136.5, C		137.4, C		137.4, C	
1′′		11.80, s		ND *^c^*		12.20, s		ND *^c^*
2′′	136.4, CH	8.55, s	137.7, CH	8.74, s	137.7, CH	8.73, d (3.0)	123.1, CH	8.53, s
3′′	112.3, C		112.1, C		112.1, C		112.1, C	
3a′′	119.5, C		126.7, C		126.7, C		119.5, C	
4′′	121.7, CH	7.92, d (8.6)	121.2, CH	8.14, d (7.8)	121.2, CH	8.09, d (8.2)	121.7, CH	7.91, d (8.4)
5′′	112.2, CH	6.70, dd (8.6, 2.0)	122.2, CH	7.19, dd (7.8, 7.6)	122.3, CH	7.22, dd (8.2, 7.7)	112.7, CH	6.69, dd (8.4, 2.1)
6′′	154.2, C		123.0, CH	7.24, dd (8.0, 7.6)	123.0, CH	7.22, dd (7.7, 7.6)	154.3, C	
7′′	97.5, CH	6.87, d (2.0)	112.4, CH	7.55, d (8.0)	112.4, CH	7.54, d (7.6)	97.5, CH	6.88, d (2.1)
7a′′	137.1, C		136.0, C		135.9, C		137.1, C	
8′′	186.2, C		186.5, C		186.3, C		185.9, C	
2-OMe	49.3, CH_3_	3.28, s	49.3, CH_3_	3.30, s	49.3, CH_3_	3.30, s	49.4, CH_3_	3.28, s

*^a^* Data were obtained in DMSO-*d*_6_. *^b^* Data were measured at 500/125 (**1** and **2**) and 800/200 (**3** and **4**) MHz for ^1^H/^13^C, respectively. *^c^* Not detected.

**Table 2:** ^13^C and ^1^H NMR assignments for compounds **5** and **6** *^a^*.

No.	5 ^*b*^		6 ^*b*^	
	ppm, Type	δ, mult (*J* in Hz)	ppm, Type	δ, mult (*J* in Hz)
1	189.7, C		189.7, C	
2	46.5, CH_2_	4.71, d (5.2)	46.6, CH_2_	4.74, d (5.2)
3		9.35, t (5.2)		9.33, t (5.2)
4	154.5, C		154.5, C	
5		10.20, s		10.20, s
1′		12.00, s		ND^*c*^
2′	133.7, CH	8.47, s	134.6, CH	8.50, s
3′	113.8, C		113.9, C	
3a′	125.4, C		124.4, C	
4′	121.1, CH	8.18, d (7.8)	122.8, CH	8.11, d (8.4)
5′	121.9, CH	7.22, dd (7.8, 7.0)	128.4, CH	7.36, dd (8.4, 1.6)
6′	122.9, CH	7.23, dd (8.0, 7.0)	114.9, C	
7′	112.2, CH	7.49, d (8.0)	115.5, CH	7.69, d (1.6)
7a′	136.5, C		137.4, C	
1′′		11.50, s		11.60, s
2′′	129.3, CH	8.34, d (2.2)	129.2, CH	8.33, s
3′′	108.4, C		108.4, C	
3a′′	119.5, C		119.4, C	
4′′	121.5, CH	7.96, d (8.5)	121.5, CH	7.95, d (8.6)
5′′	111.8, CH	6.70, dd (8.5, 2.0)	111.8, CH	6.74, dd (8.6, 2.0)
6′′	154.0, C		154.0, C	
7′′	97.1, CH	6.80, d (2.0)	97.1, CH	6.84, d (2.0)
7a′′	137.6, C		137.6, C	
8′′	165.5, C		165.5, C	

*^a^* Data were obtained in DMSO-*d*_6_. *^b^* Data were measured at 600/150 (**5**) and 800/200 (**6**) MHz. *^c^* Not detected.

**Table 3:** Results of antibacterial, sortase A inhibitory, and cytotoxic assays of compounds **1**–**14.**

	Antibacterial Activity MIC (μg/mL)								
	Gram (+) Positive			Gram (−) Negative			IC_50_ (μM)		
No.	*S. aureus*	*Enterococcus faecalis*	*Enterococcus faecium*	*Klebsiella pneumonia*	*Salmonella enterica*	*E. coli*	Srt A	A549	K562
**1**	>128	>128	>128	>128	>128	>128	>329.8	77.3	24.2
**2**	64	>128	>128	128	128	>128	62.7	55.7	28.5
**3**	32	128	>128	>128	64	>128	43.9	61.2	37.7
**4**	16	128	128	>128	64	>128	>274.7	70.9	54.2
**5**	>128	>128	>128	>128	>128	>128	79.4	>100	92.8
**6**	64	>128	>128	>128	128	>128	52.4	>100	>100
**7**	32	64	64	64	64	>128	>374.1	25.3	15.3
**8**	32	>128	>128	>128	>128	>128	41.3	22.7	12.5
**9**	32	>128	>128	>128	>128	>128	34.0	29.5	14.4
**10**	8	32	16	64	16	>128	53.2	38.9	43.2
**11**	8	32	32	8	16	>128	47.5	39.6	>100
**12**	8	16	>128	8	8	>128	>263.4	34.3	9.7
**13**	128	>128	>128	>128	>128	>128	164.4	59.2	>100
**14**	128	>128	>128	>128	>128	>128	191.1	66.8	>100
ampicillin	0.13	0.5	1	ND *^a^*	0.25	8			
tetracycline	ND *^a^*	ND *^a^*	ND *^a^*	0.25	ND *^a^*	ND *^a^*			
triphasiol							37.9		
berberine chloride							87.4		
doxorubicin								0.64	0.92

*^a^* Not detected.

## Data Availability

The data presented in this study are available in the supplementary material.

## Supplementary Materials

The following are available online at https://www.mdpi.com/1660-3397/19/1/3/s1, Figures S1–S43: 1D and 2D NMR spectra and HRFABMS data of **1**–**6**.
