# Supplementary material for: Bioactive Bis(indole) Alkaloids from a Spongosorites sp. Sponge
Source: Mar Drugs. 2020 Dec 23;19(1):3. doi: 10.3390/md19010003 (PMC7824209; doi:10.3390/md19010003)
Supplement: Supplementary file 1 [file marinedrugs-19-00003-s001.pdf]

Supporting Information

Bioactive Bis(indole) Alkaloids from a *Spongosorites* sp. Sponge

Jae Sung Park [1], Eunji Cho [2], Ji-Yeon Hwang [1], Sung Chul Park [1], Beomkoo Chung [2], Oh-Seok Kwon [1], Chung J. Sim [3], Dong-Chan Oh [1], Ki-Bong Oh [2,*] and Jongheon Shin [1,*]

[1] Natural Products Research Institute, College of Pharmacy, Seoul National University, San 56-1, Sillim, Gwanak, Seoul 151-742, Korea; jaesung89@snu.ac.kr (J.S.P.); yahyah7@snu.ac.kr (J.-Y.H.); sungchulpark@snu.ac.kr (S.C.P.); ideally225@snu.ac.kr (O.-S.K.); dongchanoh@snu.ac.kr (D.-C.O.)

[2] Department of Agricultural Biotechnology, College of Agriculture and Life Sciences, Seoul National University, Seoul 08826, Korea; eunji525@snu.ac.kr (E.C.); beomkoo01@snu.ac.kr (B.C.)

[3] Department of Biological Sciences, College of Life Science and Nano Technology, Hannam University, 461-6 Jeonmin, Yuseong, Daejeon 305-811, Korea; cjsim@hnu.kr

* Correspondence: ohkibong@snu.ac.kr (K.-B.O.); shinj@snu.ac.kr (J.S.); Tel.: +82-2-880-4646 (K.-B.O.); +82-2-880-2484 (J.S.)

Contents

Figure S1. The ^{1}H NMR (800 MHz, DMSO-*d6*) spectrum of **1** S4

Figure S2. The ^{13}C NMR (200 MHz, DMSO-*d6*) spectrum of **1** S5

Figure S3. The HSQC (800 MHz, DMSO-*d6*) spectrum of **1** S6

Figure S4. The COSY (800 MHz, DMSO-*d6*) spectrum of **1** S7

Figure S5. The HMBC (800 MHz, DMSO-*d6*) spectrum of **1** S8

Figure S6. The LRHSQMBC at 2 Hz (800 MHz, DMSO-*d6*) spectrum of **1** S9

Figure S7. The HRFABMS data of **1** S10

Figure S8. The ^{1}H NMR (800 MHz, DMSO-*d6*) spectrum of **2** S11

Figure S9. The ^{13}C NMR (200 MHz, DMSO-*d6*) spectrum of **2** S12

Figure S10. The HSQC spectrum (800 MHz, DMSO-*d6*) spectrum of **2** S13

Figure S11. The COSY spectrum (800 MHz, DMSO-*d6*) spectrum of **2** S14

Figure S12. The HMBC spectrum (800 MHz, DMSO-*d6*) spectrum of **2** S15

Figure S13. The HRFABMS data of **2** S16

Figure S14. The ^{1}H NMR (800 MHz, DMSO-*d6*) spectrum of **3** S17

Figure S15. The ^{13}C NMR (200 MHz, DMSO-*d6*) spectrum of **3** S18

Figure S16. The HSQC (800 MHz, DMSO-*d6*) spectrum of **3** S19

Figure S17. The COSY (800 MHz, DMSO-*d6*) spectrum of **3** S20

Figure S18. The HMBC (800 MHz, DMSO-*d6*) spectrum of **3** S21

Figure S19. The dHMBC at 2Hz (800 MHz, DMSO-*d6*) spectrum of **3** S22

Figure S20. The HRFABMS data of **3** S23

Figure S21. The ^{1}H NMR (800 MHz, DMSO-*d6*) spectrum of **4** S24

Figure S22. The ^{13}C NMR (200 MHz, DMSO-*d6*) spectrum of **4** S25

Figure S23. The HSQC (800 MHz, DMSO-*d6*) spectrum of **4** S26

Figure S24. The COSY (800 MHz, DMSO-*d6*) spectrum of **4** S27

Figure S25. The HMBC (800 MHz, DMSO-*d6*) spectrum of **4** S28
Figure S26. The HRFABMS data of **4** S29
Figure S27. The ^{1}H NMR (800 MHz, DMSO-*d6*) spectrum of **5** S30
Figure S28. The ^{13}C NMR (200 MHz, DMSO-*d6*) spectrum of **5** S31
Figure S29. The HSQC (800 MHz, DMSO-*d6*) spectrum of **5** S32
Figure S30. The COSY (800 MHz, DMSO-*d6*) spectrum of **5** S33
Figure S31. The HMBC (800 MHz, DMSO-*d6*) spectrum of **5** S34
Figure S32. The HRFABMS data of **5** S35
Figure S33. The ^{1}H NMR (800 MHz, DMSO-*d6*) spectrum of **6** S36
Figure S34. The ^{13}C NMR (200 MHz, DMSO-*d6*) spectrum of **6** S37
Figure S35. The HSQC (800 MHz, DMSO-*d6*) spectrum of **6** S38
Figure S36. The COSY (800 MHz, DMSO-*d6*) spectrum of **6** S39
Figure S37. The HMBC (800 MHz, DMSO-*d6*) spectrum of **6** S40
Figure S38. The dHMBC at 4Hz (800 MHz, DMSO-*d6*) spectrum of **6** S41
Figure S39. The HRFABMS data of **6** S42
Figure S40. Calculated optical rotations of major conformers of **1** S43
Figure S41. Calculated optical rotations of major conformers of **2** S44
Figure S42. Calculated optical rotations of major conformers of **3** S45
Figure S43. Calculated optical rotations of major conformers of **4** S46

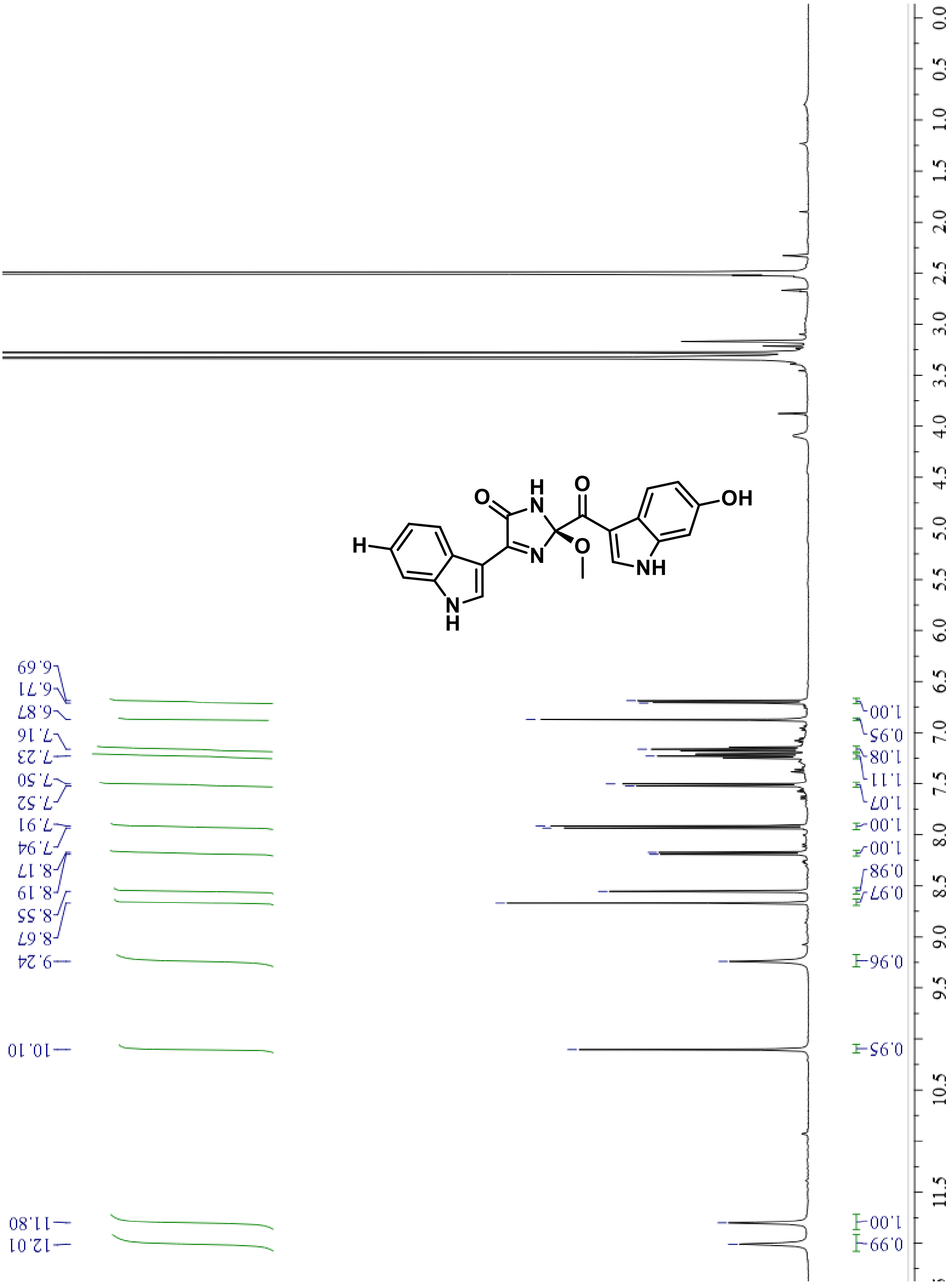

Figure S1. The ^{1}H NMR (800 MHz, DMSO-*d6*) spectrum of **1.**

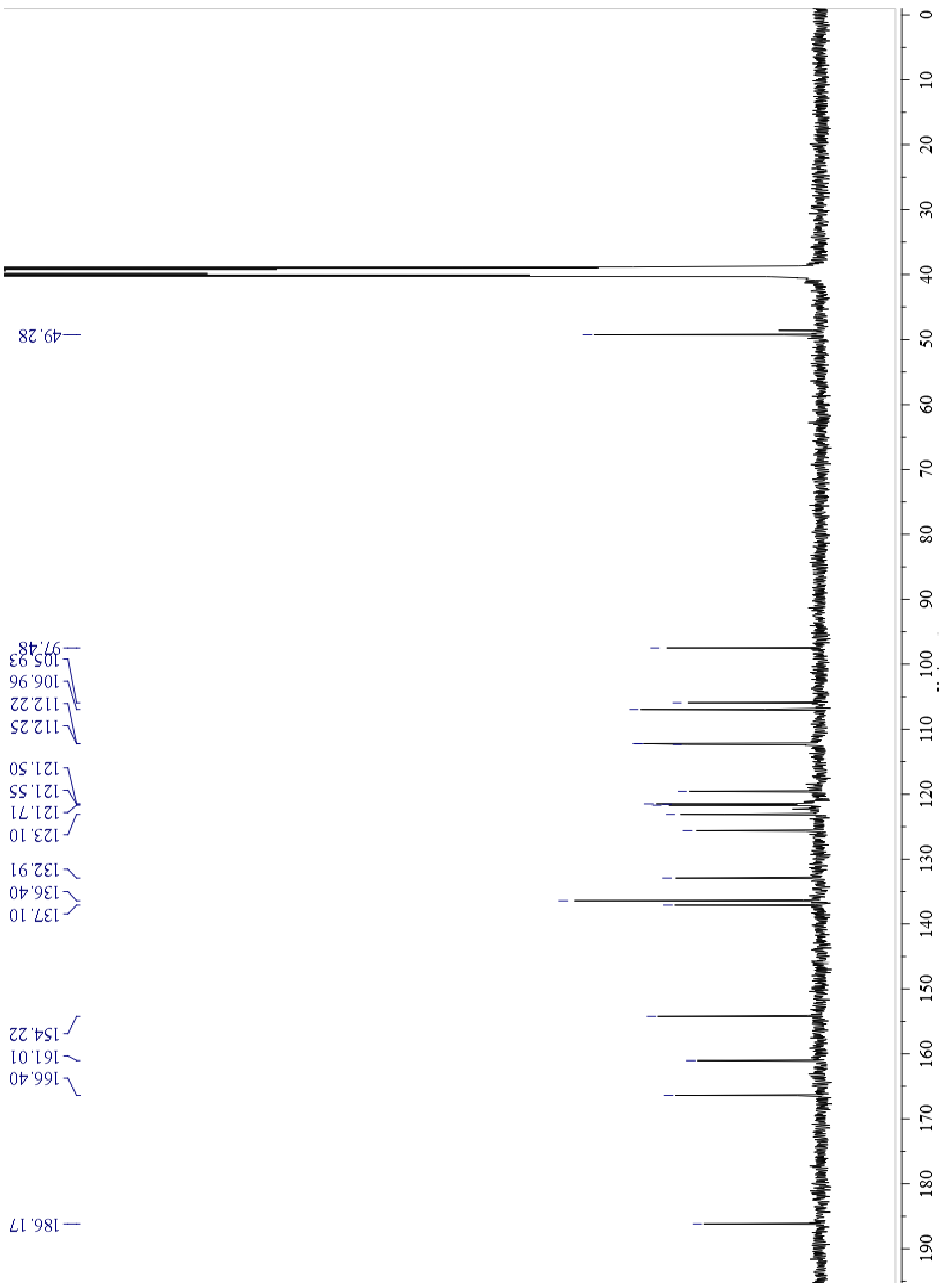

Figure S2. The ^{13}C NMR (200 MHz, DMSO-*d6*) spectrum of **1.**

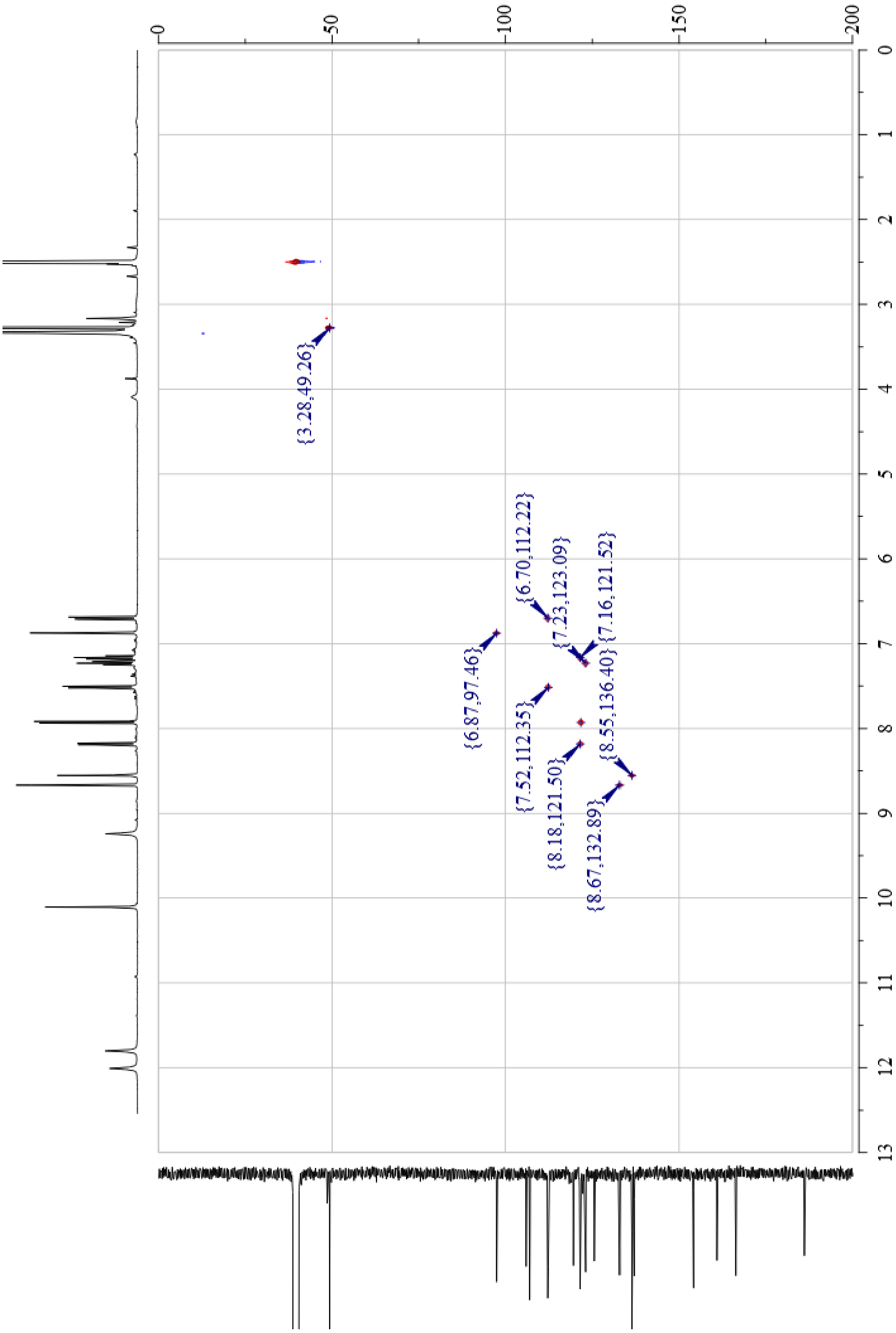

Figure S3. The HSQC (800 MHz, DMSO-*d6*) spectrum of **1.**

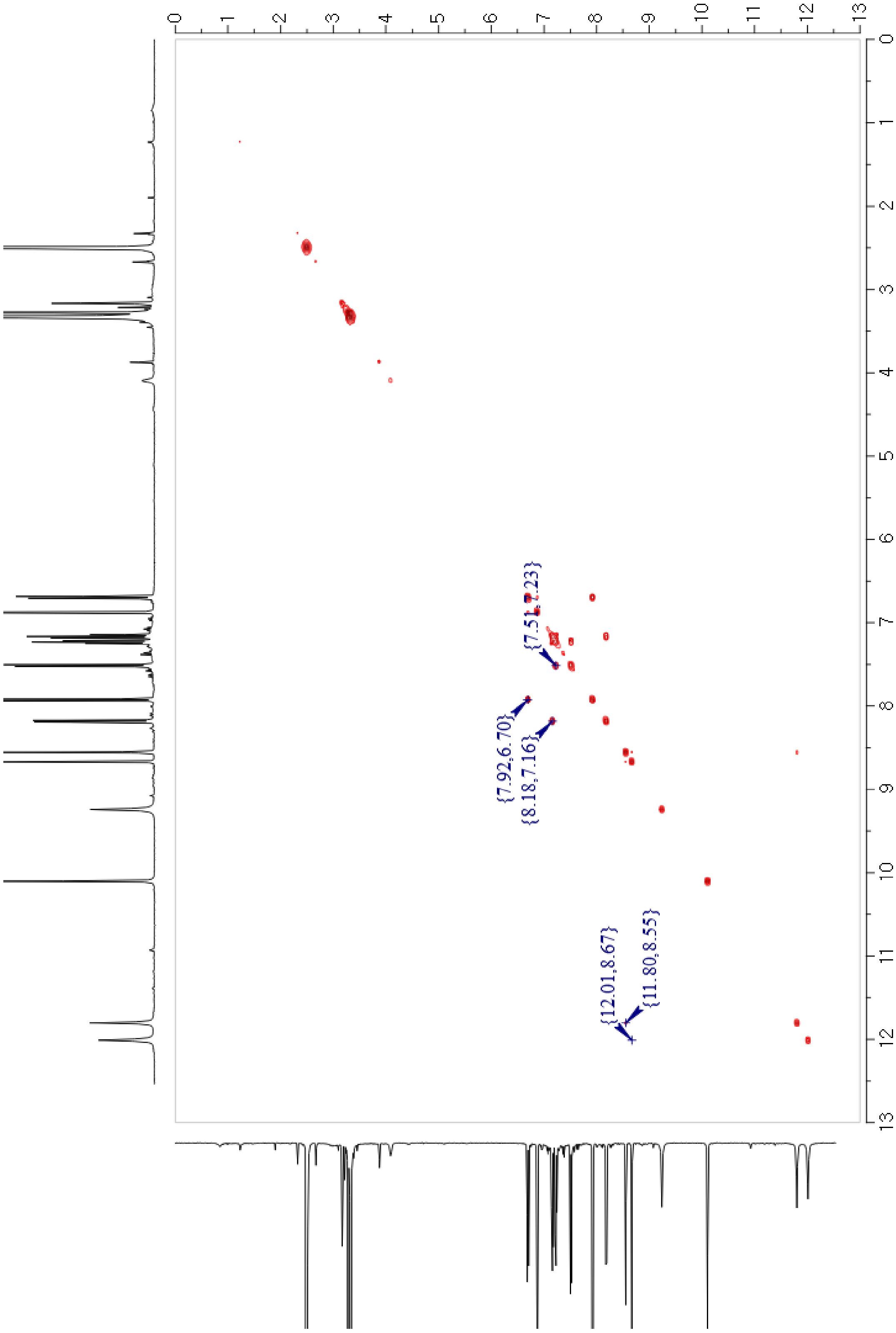

Figure S4. The COSY (800 MHz, DMSO-*d6*) spectrum of **1.**

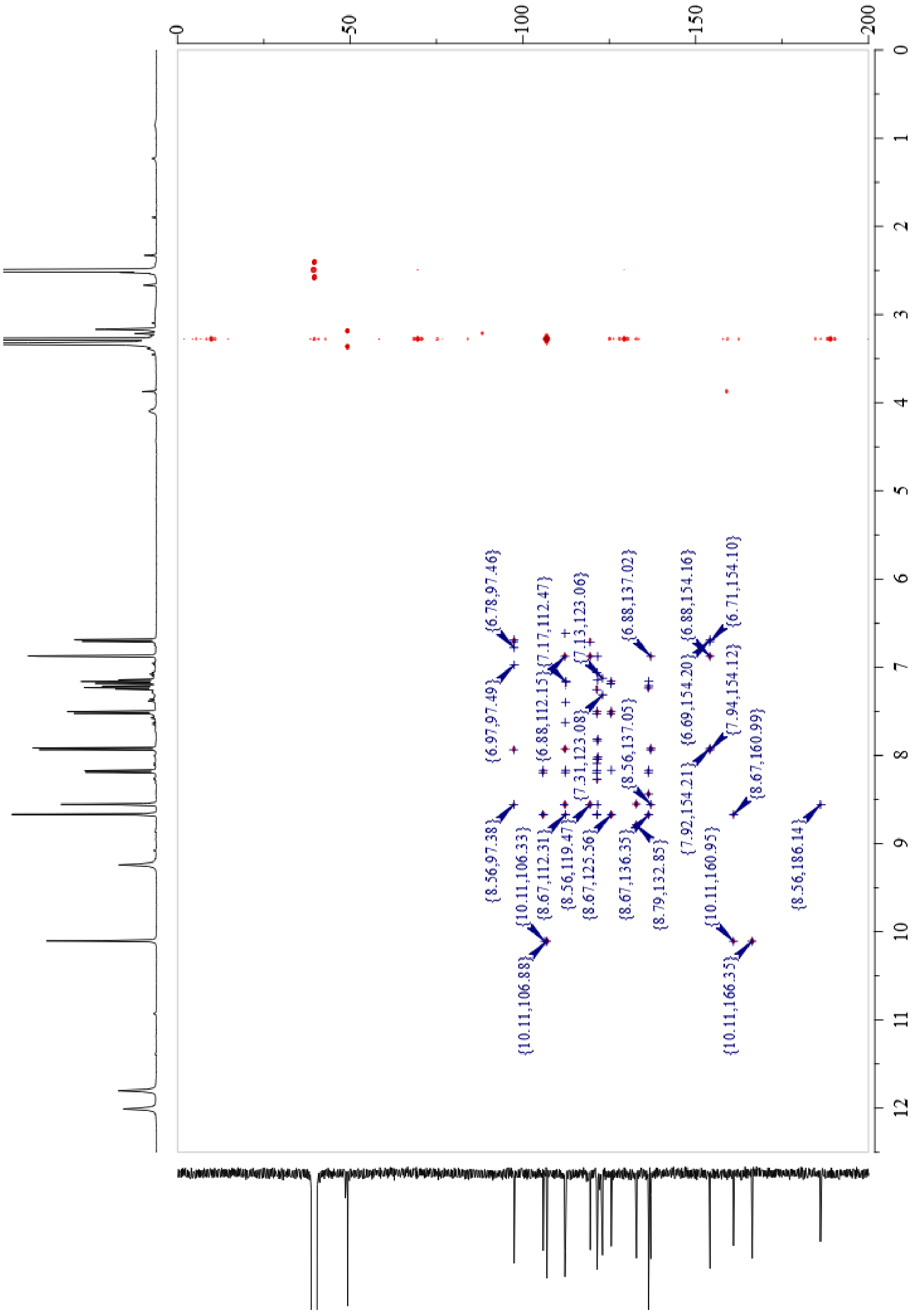

Figure S5. The HMBC (800 MHz, DMSO-*d6*) spectrum of **1.**

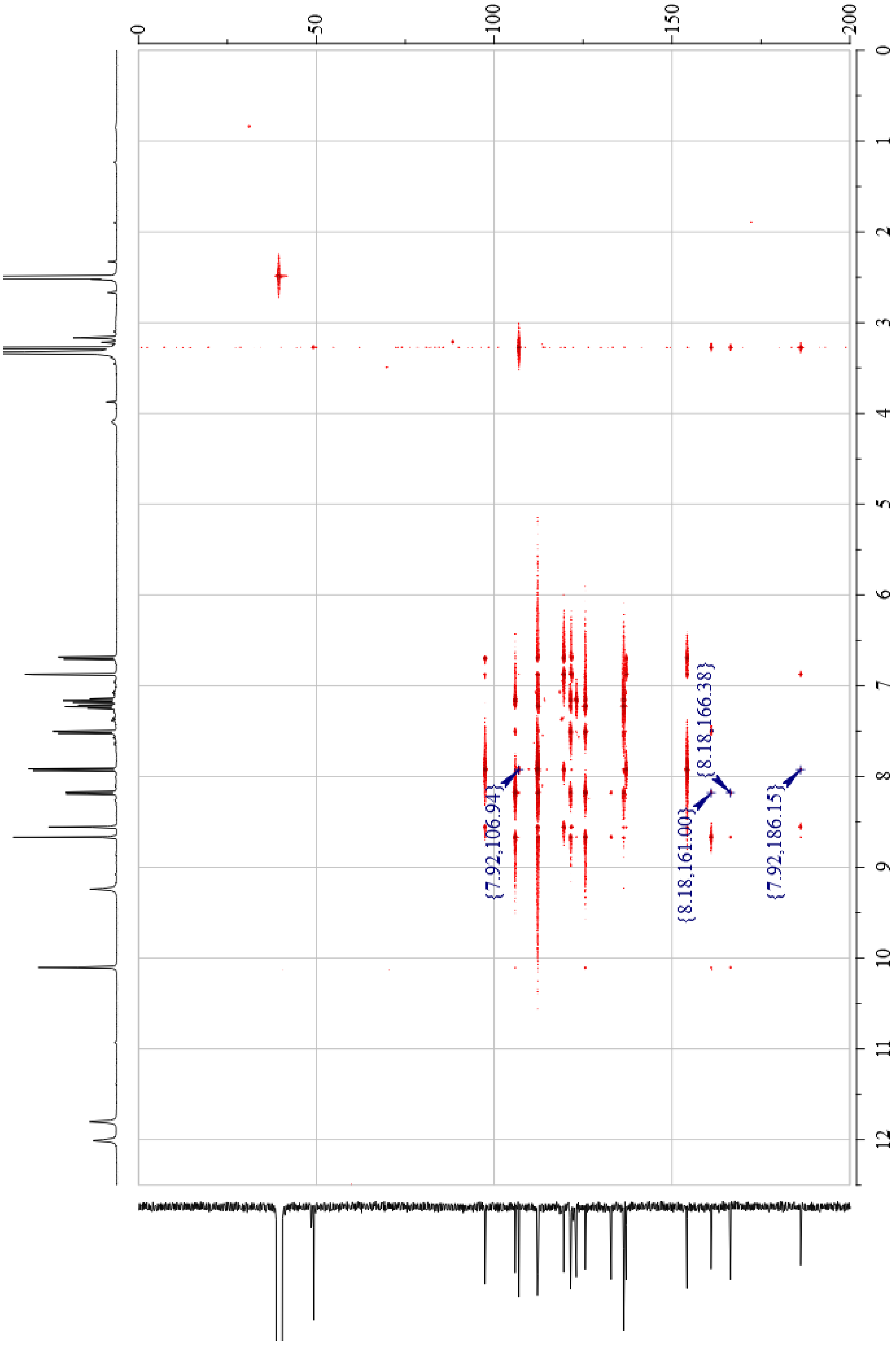

Figure S6. The LRHSQMBC at 2 Hz (800 MHz, DMSO-*d6*) spectrum of **1.**

Observed m/z	Int%	Err[ppm / mmu]	U.S.	Composition
389.1248	5.1	+11.1 / +4.3	24.0	C 30 H 15 N
		-4.0 / -1.6	20.5	C 22 H 13 N 8
		-7.5 / -2.9	20.0	C 24 H 15 N 5 O
		-10.9 / -4.2	19.5	C 26 H 17 N 2 O 2
		+6.3 / +2.5	16.5	C 17 H 13 N 10 O 2
		+18.0 / +7.0	19.5	C 27 H 17 O 3
		+2.9 / +1.1	16.0	C 19 H 15 N 7 O 3
		-0.6 / -0.2	15.5	C 21 H 17 N 4 O 4
		-4.0 / -1.6	15.0	C 23 H 19 N O 5
		-19.1 / -7.4	11.5	C 15 H 17 N 8 O 5
		+13.2 / +5.1	12.0	C 14 H 15 N 9 O 5

[Theoretical Ion Distribution] Page: 1

Molecular Formula : C21 H17 N4 O4

(m/z 389.1250, MW 389.3904, U.S. 15.5)

Base Peak : 389.1250, Averaged MW : 389.3889(a), 389.3896(w)

m/z	INT.	
389.1250	100.0000	**
390.1280	24.9787	**************
391.1306	3.7890	**
392.1331	0.4276	
393.1356	0.0387	
394.1381	0.0029	
395.1405	0.0002	

Figure S7. The HRFABMS data of **1**

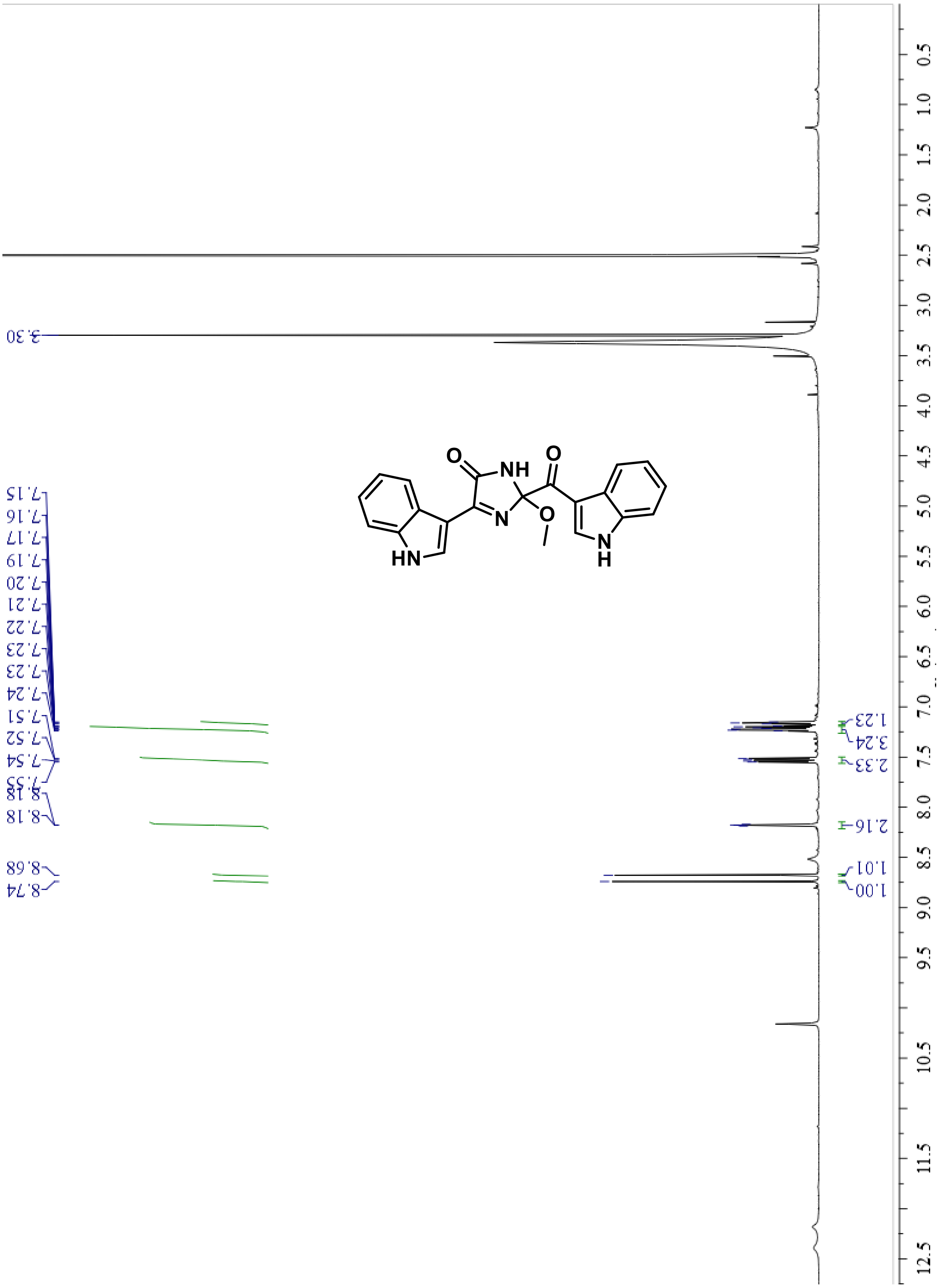

Figure S8. The ^{1}H NMR (800 MHz, DMSO-*d6*) spectrum of **2**

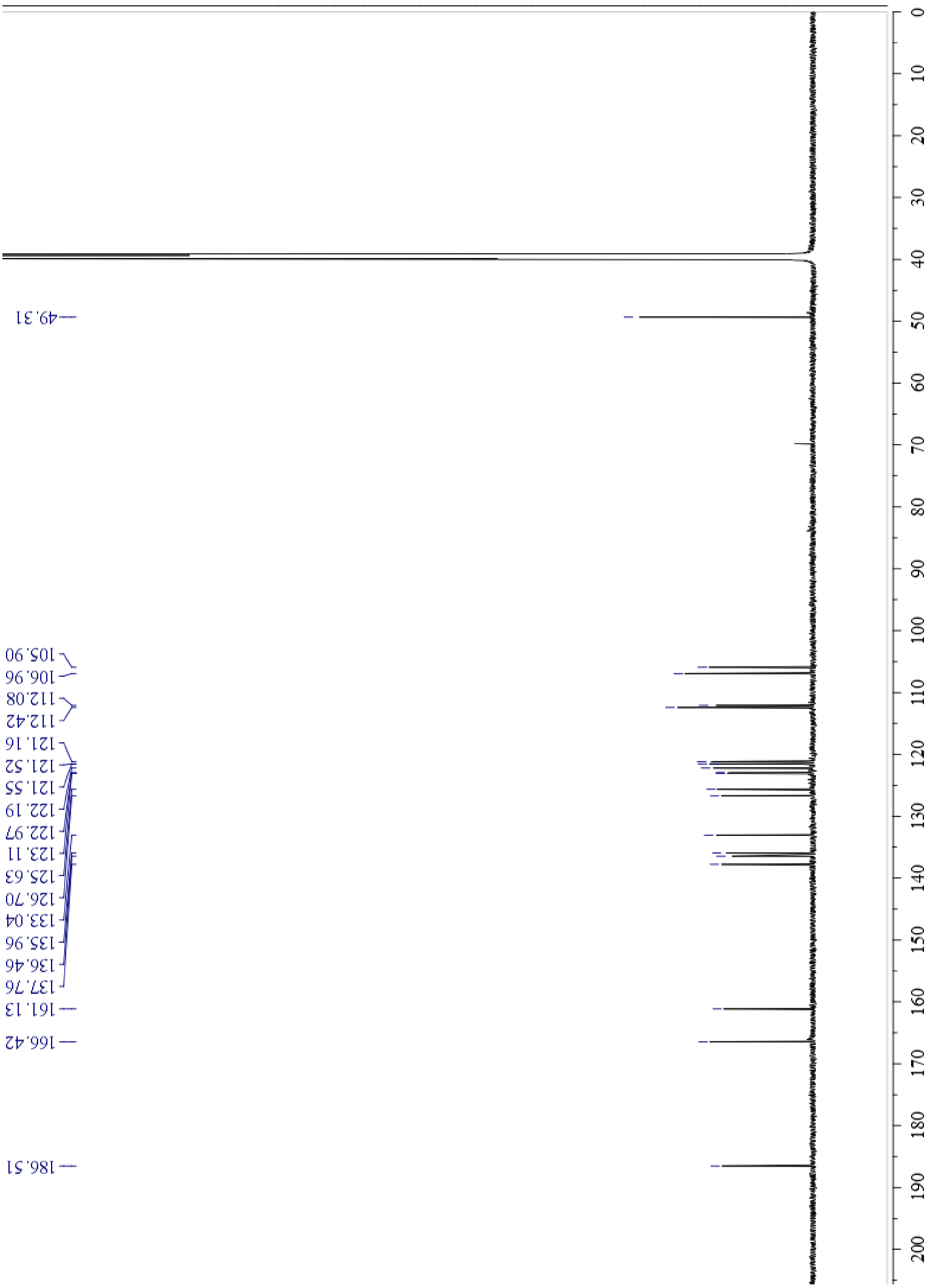

Figure S9. The ^{13}C NMR (200 MHz, DMSO-*d6*) spectrum of **2**

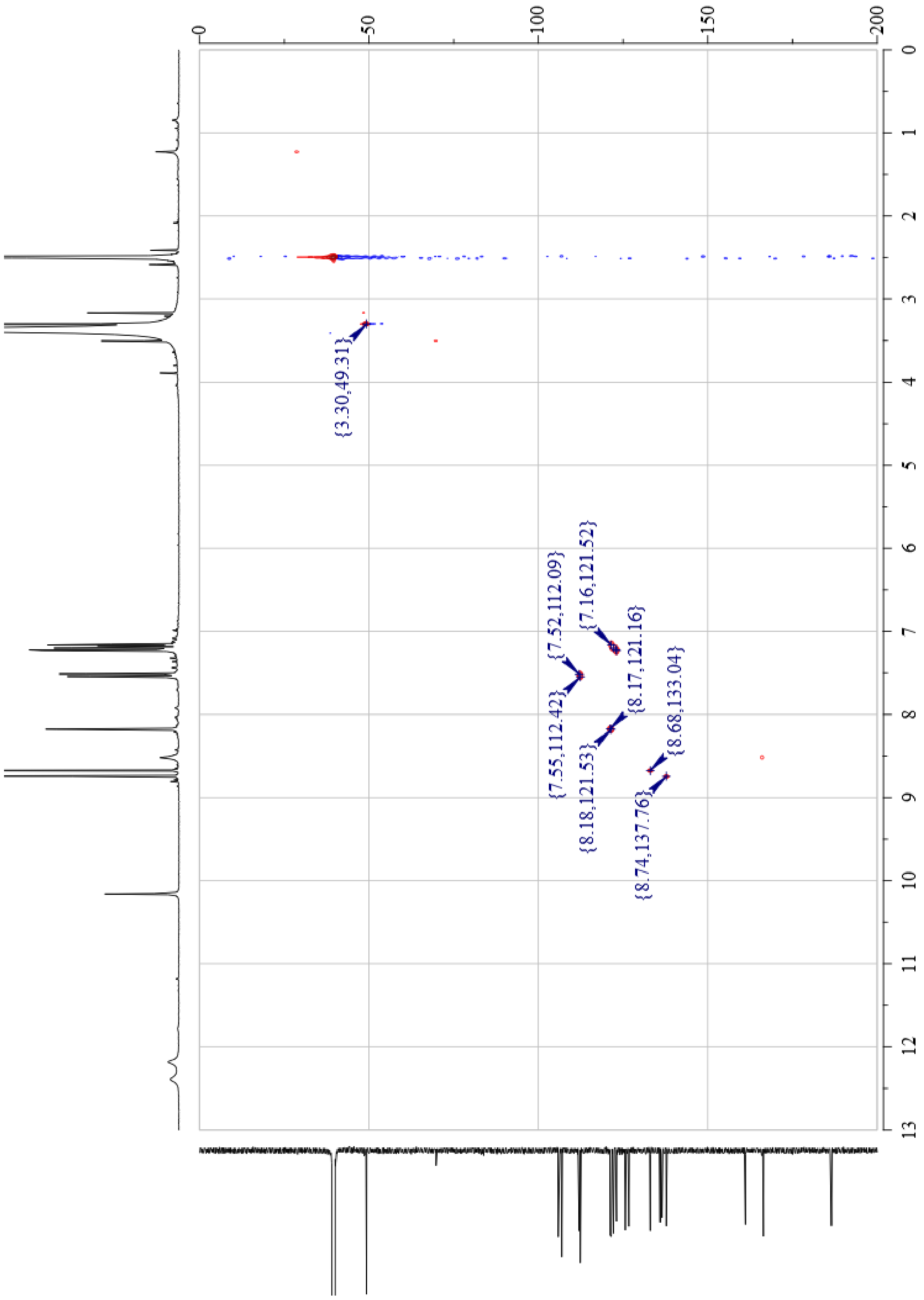

Figure S10. The HSQC spectrum (800 MHz, DMSO-*d6*) spectrum of **2**

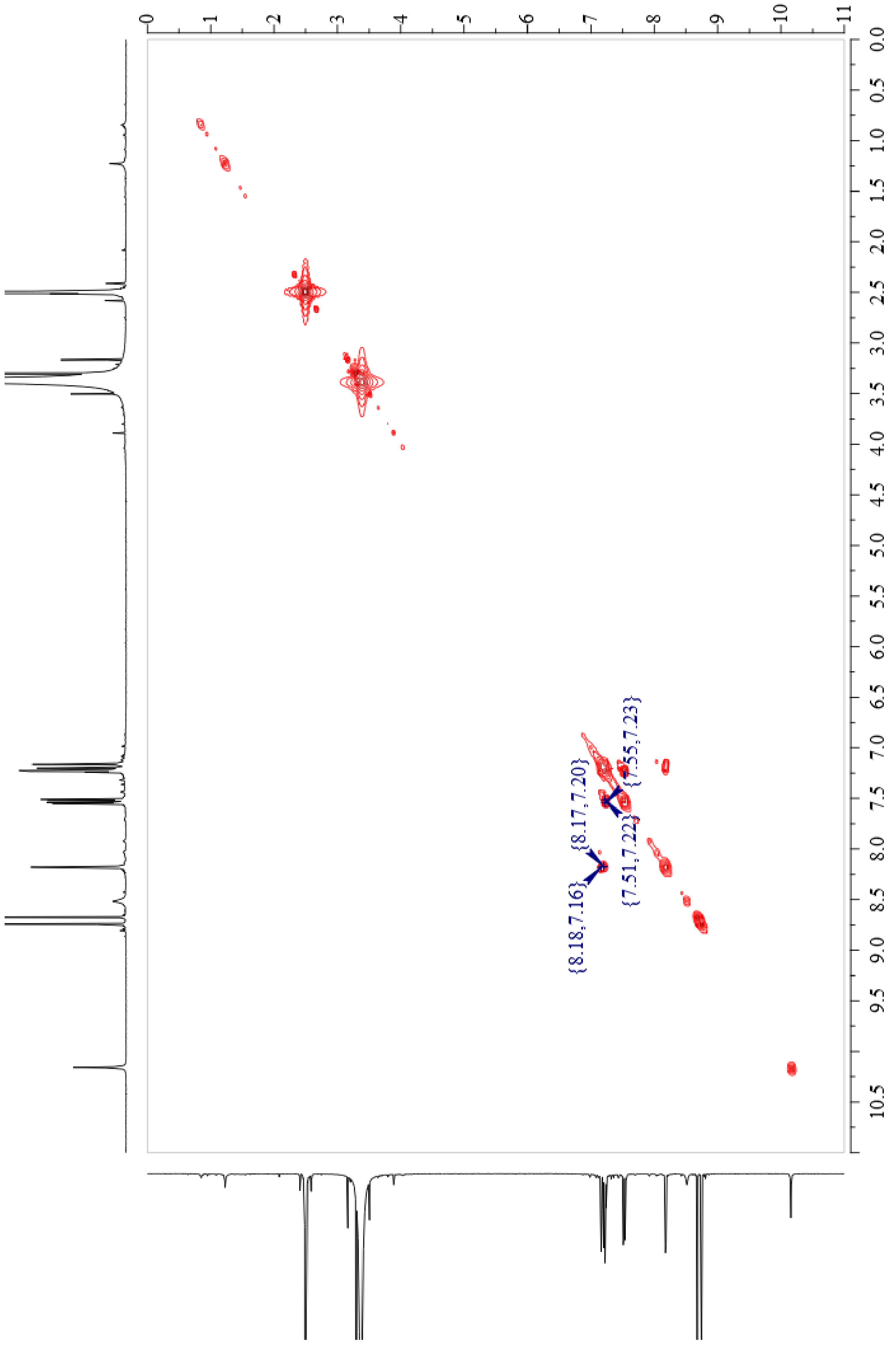

Figure S11. The COSY spectrum (800 MHz, DMSO-*d6*) spectrum of **2**

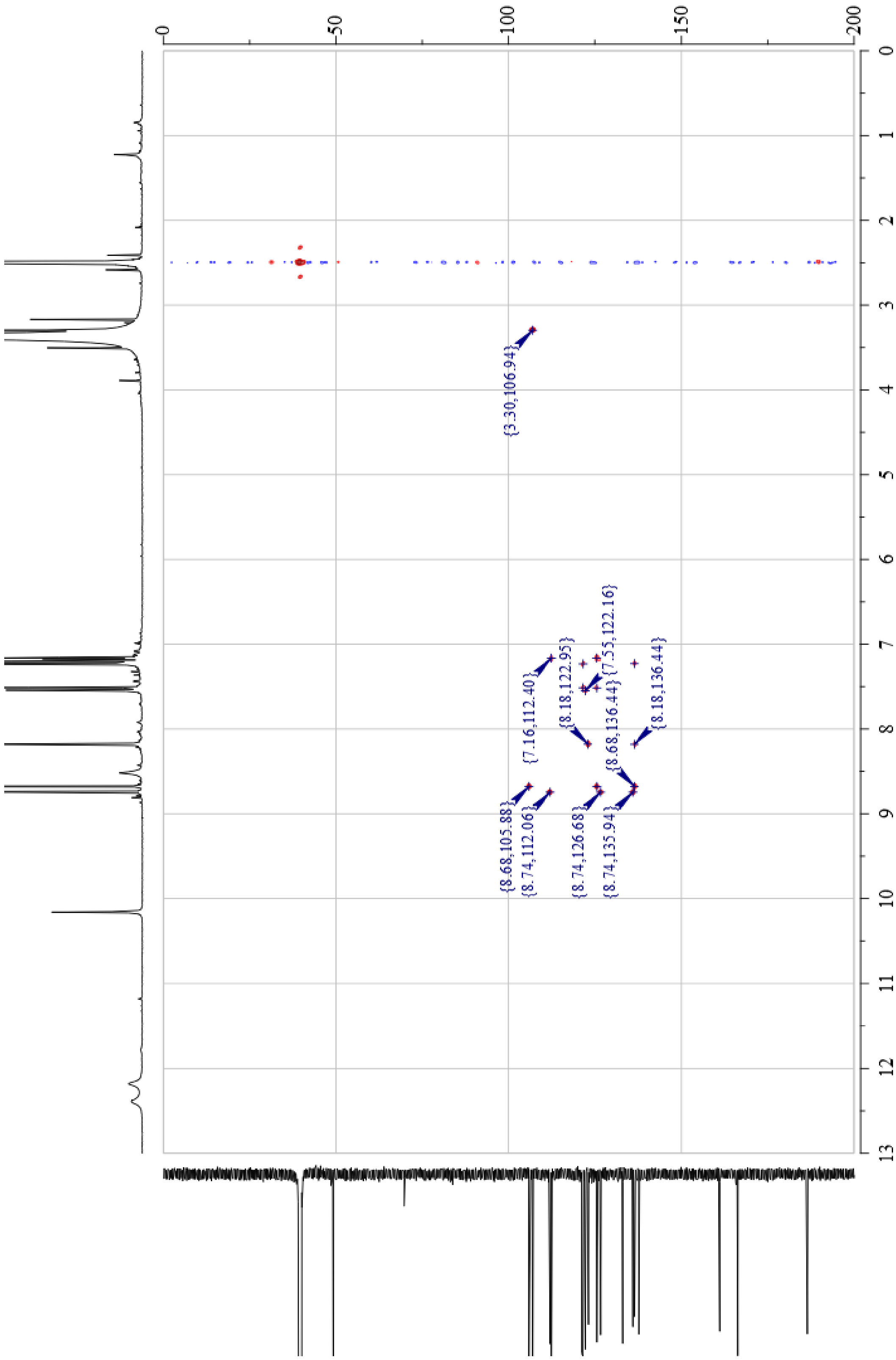

Figure S12. The HMBC spectrum (800 MHz, DMSO-*d6*) spectrum of **2**

Observed m/z	Int%	Err[ppm / mmu]	U.S.	Composition
373.1303	5.0	-6.4 / -2.4	20.0	C 24 H 15 N 5
		-10.0 / -3.7	19.5	C 26 H 17 N 2 O
		+7.9 / +3.0	16.5	C 17 H 13 N 10 O
		+4.3 / +1.6	16.0	C 19 H 15 N 7 O 2
		+0.7 / +0.3	15.5	C 21 H 17 N 4 O 3
		-2.9 / -1.1	15.0	C 23 H 19 N O 4
		-18.6 / -6.9	11.5	C 15 H 17 N 8 O 4
		+15.1 / +5.6	12.0	C 14 H 15 N 9 O 4
		+11.5 / +4.3	11.5	C 16 H 17 N 6 O 5
		+7.9 / +3.0	11.0	C 18 H 19 N 3 O 6
		-7.8 / -2.9	7.5	C 10 H 17 N 10 O 6
		+4.3 / +1.6	10.5	C 20 H 21 O 7
		-11.4 / -4.3	7.0	C 12 H 19 N 7 O 7
		-15.0 / -5.6	6.5	C 14 H 21 N 4 O 8
		+18.7 / +7.0	7.0	C 13 H 19 N 5 O 8
		-18.6 / -6.9	6.0	C 16 H 23 N O 9
		+15.1 / +5.6	6.5	C 15 H 21 N 2 O 9
		-0.6 / -0.2	3.0	C 7 H 19 N 9 O 9
		-4.2 / -1.6	2.5	C 9 H 21 N 6 O 10

[Theoretical Ion Distribution] Page: 1
Molecular Formula : C21 H17 N4 O3
(m/z 373.1301, MW 373.3910, U.S. 15.5)
Base Peak : 373.1301, Averaged MW : 373.3896(a), 373.3903(w)

m/z	INT.	
373.1301	100.0000	**
374.1331	24.9406	***************
375.1357	3.5790	**
376.1383	0.3762	
377.1408	0.0314	
378.1433	0.0022	
379.1459	0.0001	

Figure S13. The HRFABMS data of **2**

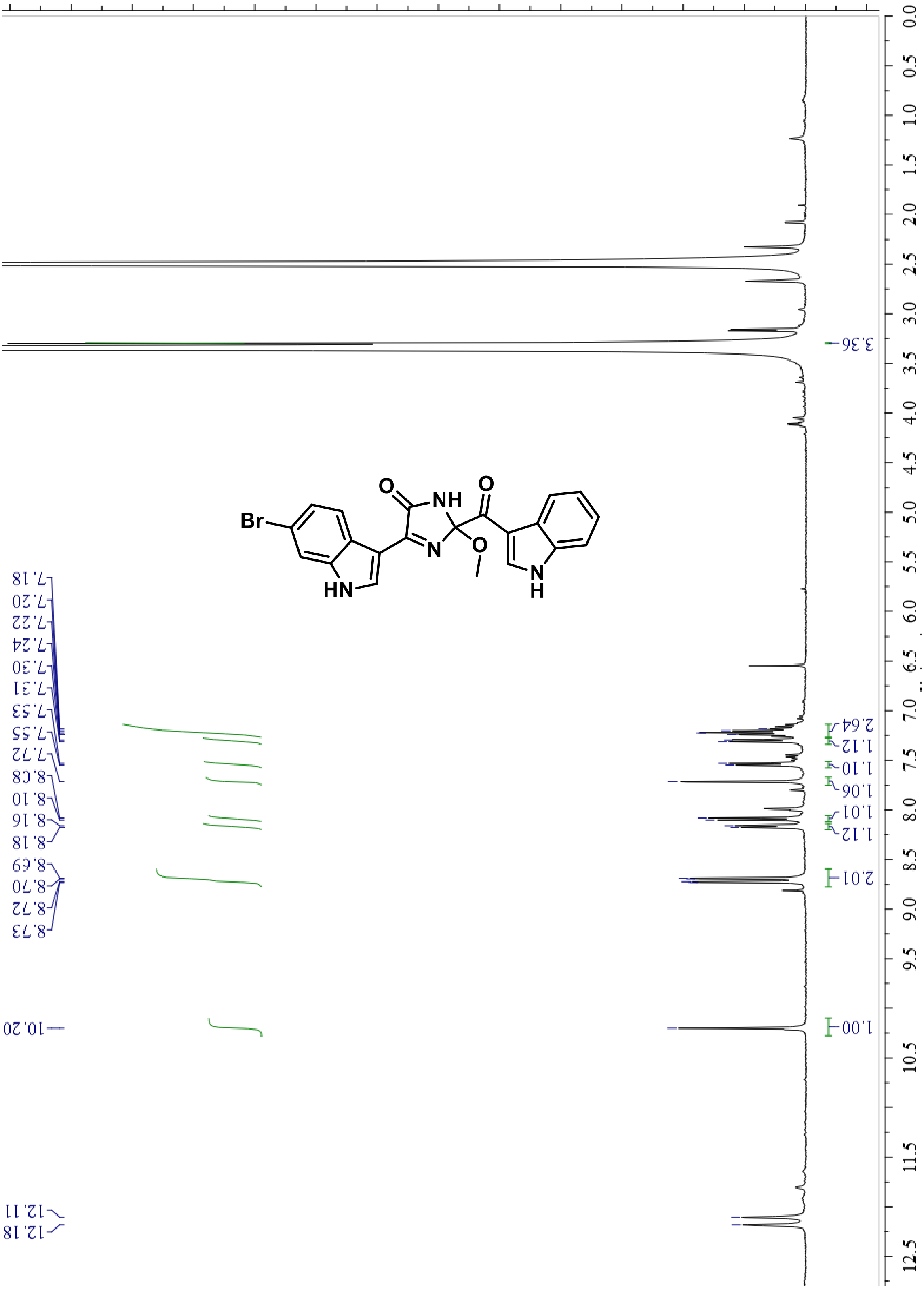

Figure S14. The ^{1}H NMR (800 MHz, DMSO-*d6*) spectrum of **3**

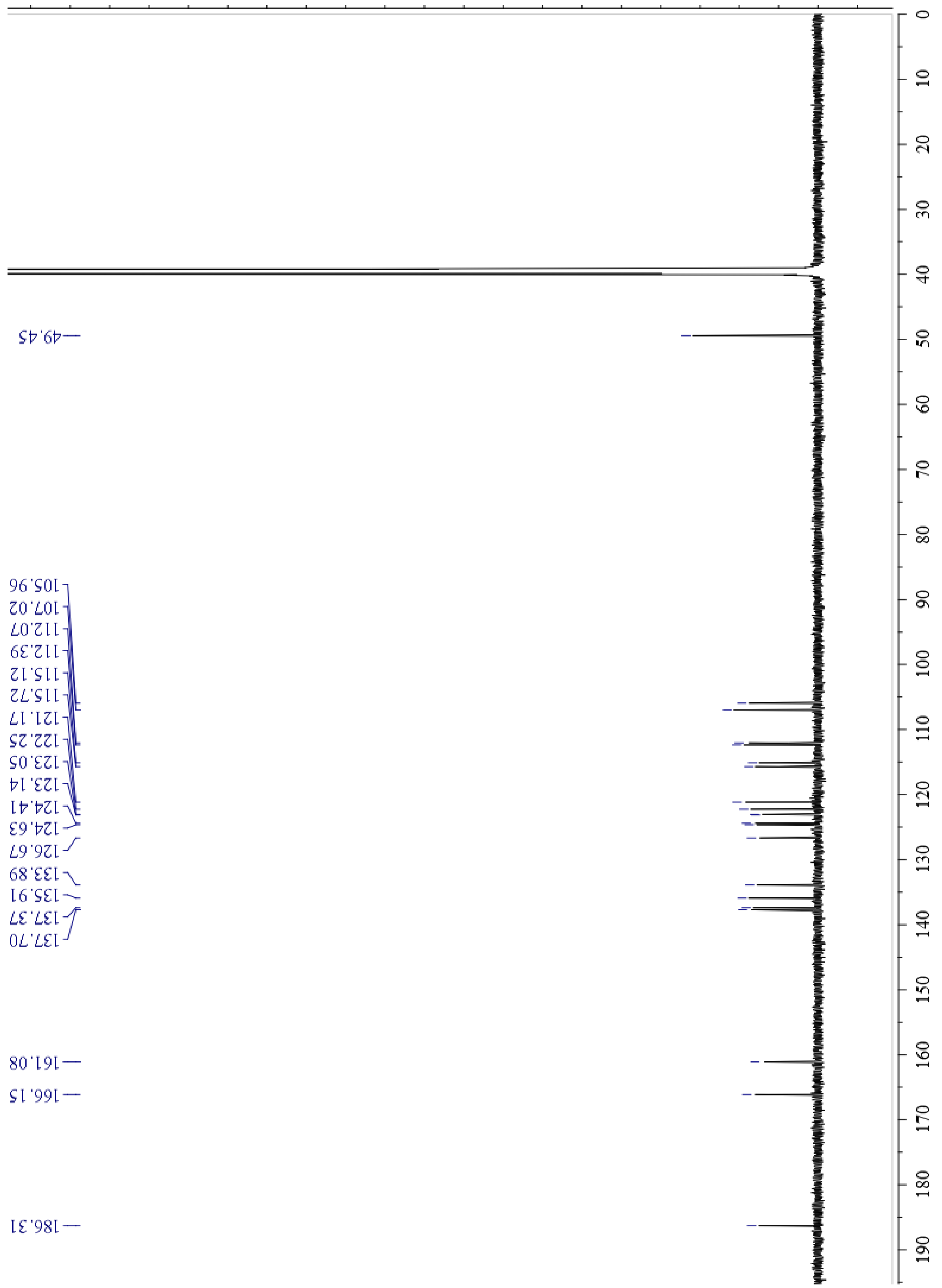

Figure S15. The ^{13}C NMR (200 MHz, DMSO-*d6*) spectrum of **3**

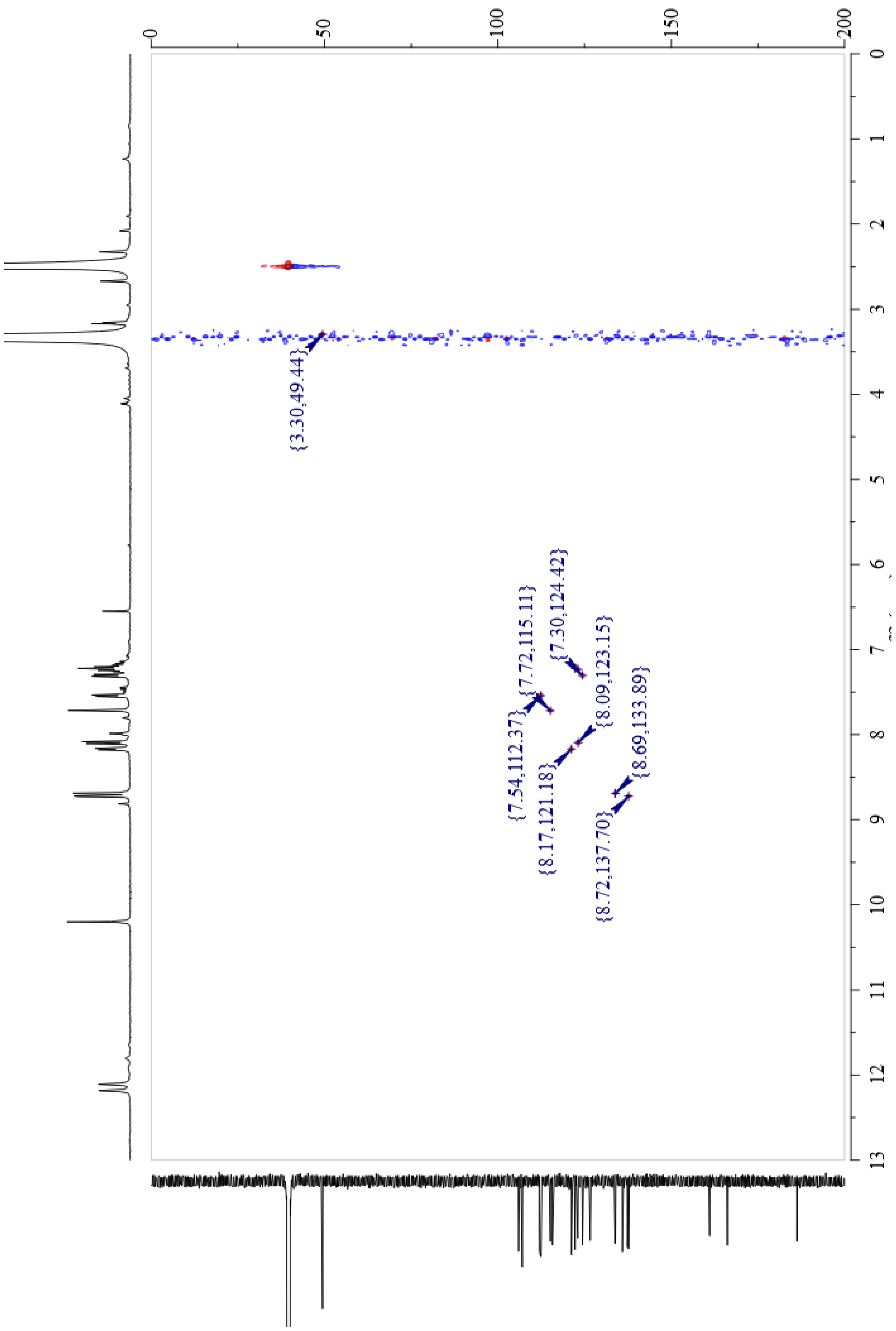

Figure S16. The HSQC (800 MHz, DMSO-*d6*) spectrum of **3**

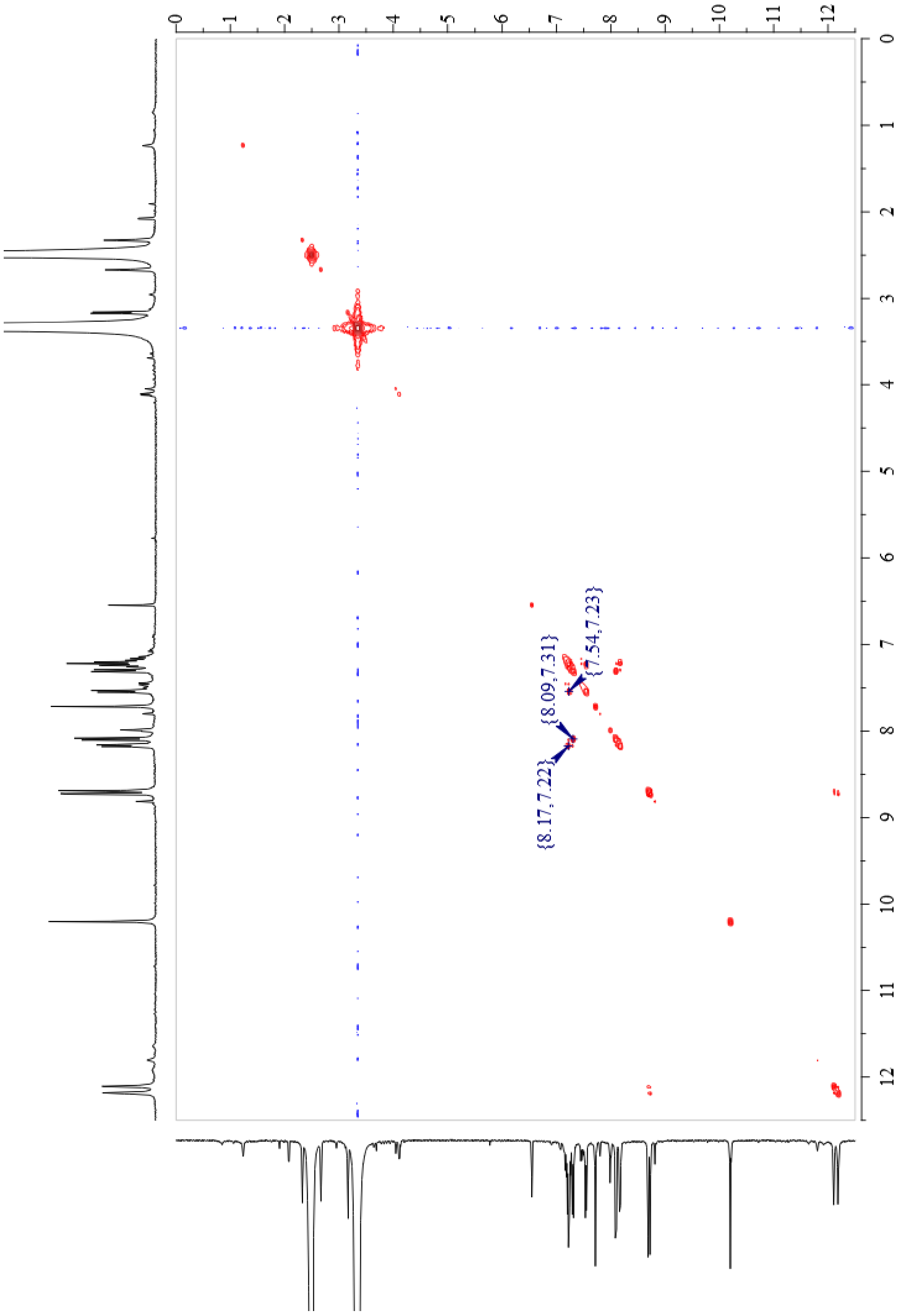

Figure S17. The COSY (800 MHz, DMSO-*d6*) spectrum of **3**

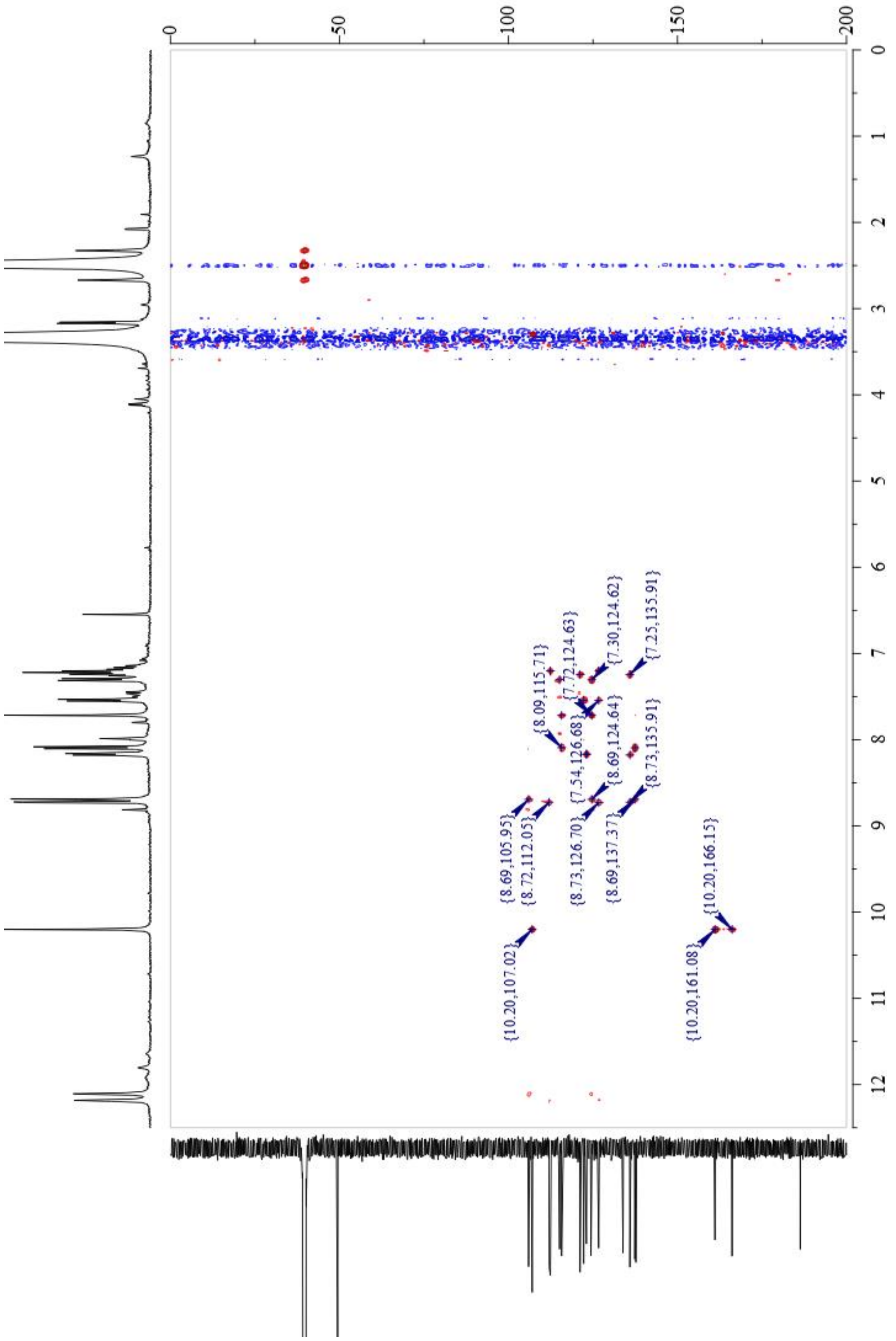

Figure S18. The HMBC (800 MHz, DMSO-*d6*) spectrum of **3**

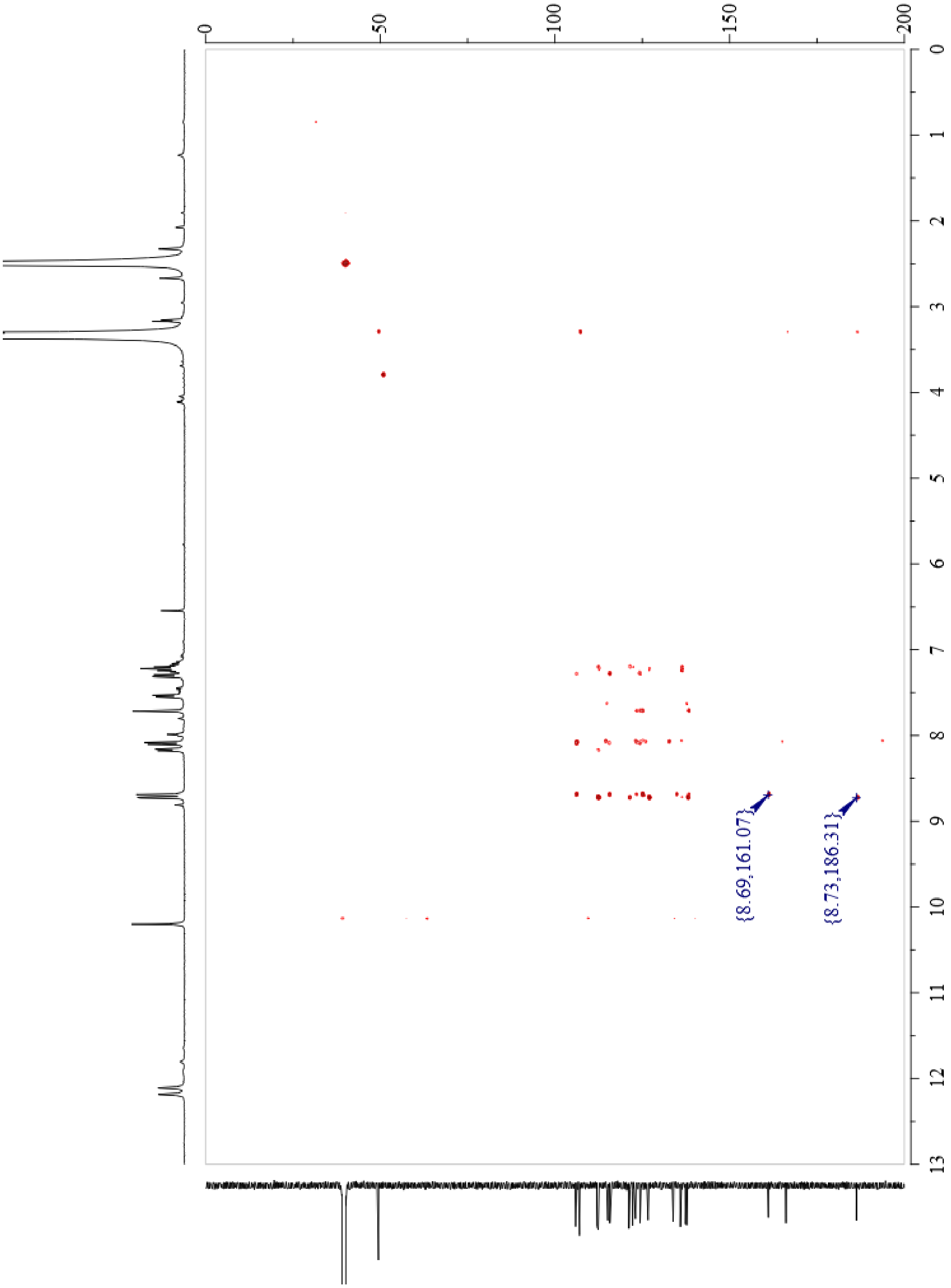

Figure S19. The dHMBC at 2Hz (800 MHz, DMSO-*d6*) spectrum of **3**

	Observed m/z	Int%	Err[ppm / mmu]	U.S.	Composition
1	451.0413	4.07	-10.2 / -4.6	8.5	C22 H27 79Br 81Br
2			+17.7 / +8.0	9.0	C21 H25 79Br 81Br N
3			-20.9 / -9.4	20.0	C26 H16 81Br N3
4			-4.3 / -2.0	20.0	C24 H14 79Br N5
5			+4.0 / +1.8	20.0	C27 H16 81Br N O
6			-7.3 / -3.3	19.5	C26 H16 79Br N2 O
7			+20.6 / +9.3	20.0	C25 H14 79Br N3 O
8			+1.7 / +0.8	5.0	C15 H25 79Br 81Br N5 O
9			+17.6 / +7.9	19.5	C27 H16 79Br O2
10			-1.3 / -0.6	4.5	C17 H27 79Br 81Br N2 O2
11			-11.9 / -5.4	16.0	C21 H16 81Br N5 O2
12			-14.9 / -6.7	15.5	C23 H18 81Br N2 O3
13			+13.0 / +5.8	16.0	C22 H16 81Br N3 O3
14			+1.6 / +0.7	15.5	C21 H16 79Br N4 O3
15			+10.0 / +4.5	15.5	C24 H18 81Br O4
16			-1.4 / -0.6	15.0	C23 H18 79Br N O4
17			-20.2 / -9.1	0.0	C13 H29 79Br 81Br N3 O4
18			+7.6 / +3.4	0.5	C12 H27 79Br 81Br N4 O4
19			+4.7 / +2.1	0.0	C14 H29 79Br 81Br N O5
20			-6.0 / -2.7	11.5	C18 H18 81Br N4 O5
21			-17.4 / -7.8	11.0	C17 H18 79Br N5 O5
22			+21.9 / +9.9	12.0	C17 H16 81Br N5 O5

[Theoretical Ion Distribution]
Molecular Formula : C21 H16 Br N4 O3
(m/z 451.0406, MW 452.2870, U.S. 15.5)
Base Peak : 453.0388, Averaged MW : 452.2853(a), 452.2881(w)

m/z	INT.
451.0406	99.1507**
452.0436	24.7288**************
453.0388	100.0000**
454.0416	24.4286**************
455.0443	3.4831**
456.0468	0.3650
457.0493	0.0304
458.0518	0.0021
459.0543	0.0001

Figure S20. The HRFABMS data of **3**

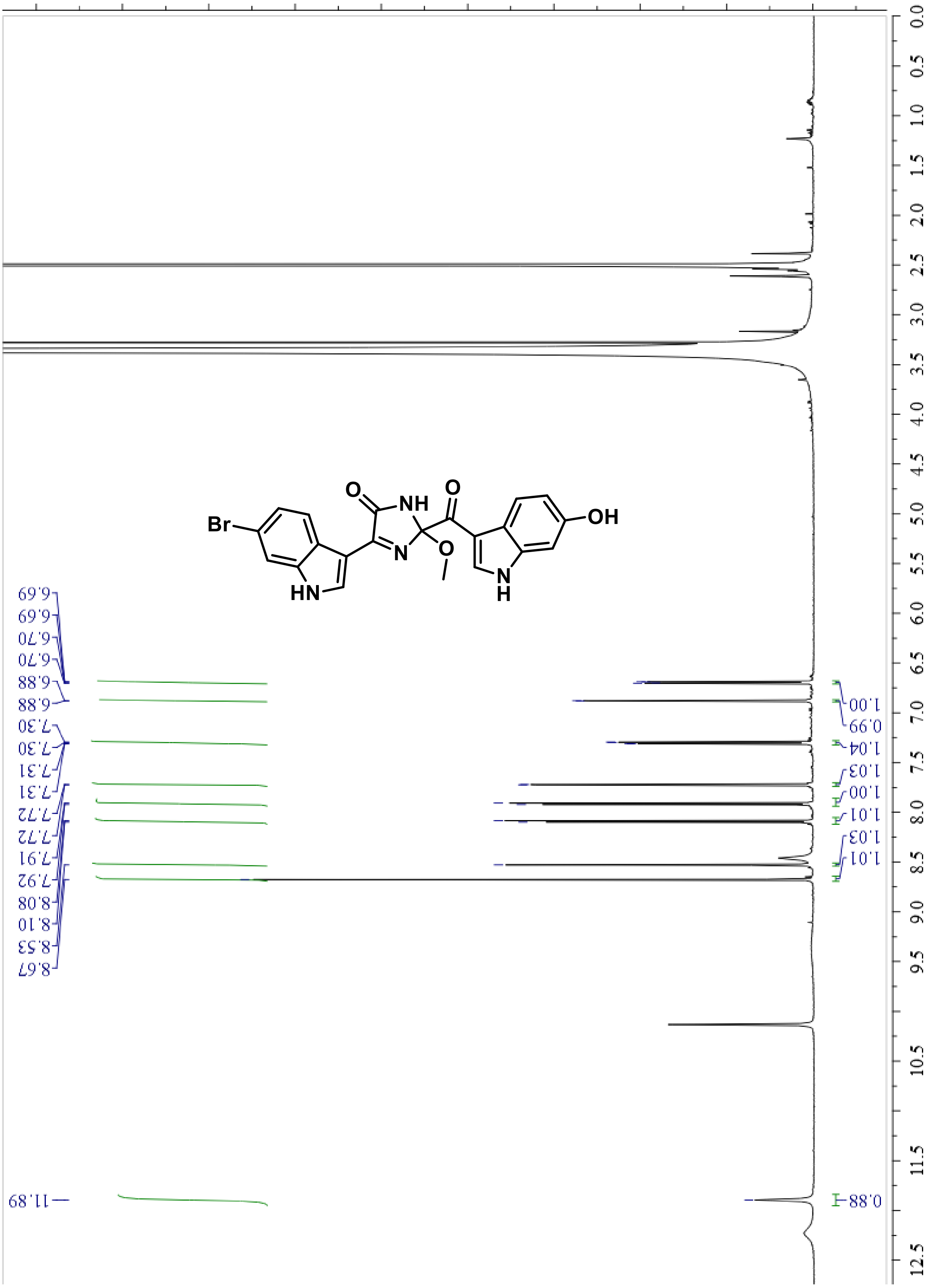

Figure S21. The ^{1}H NMR (800 MHz, DMSO-*d6*) spectrum of **4**

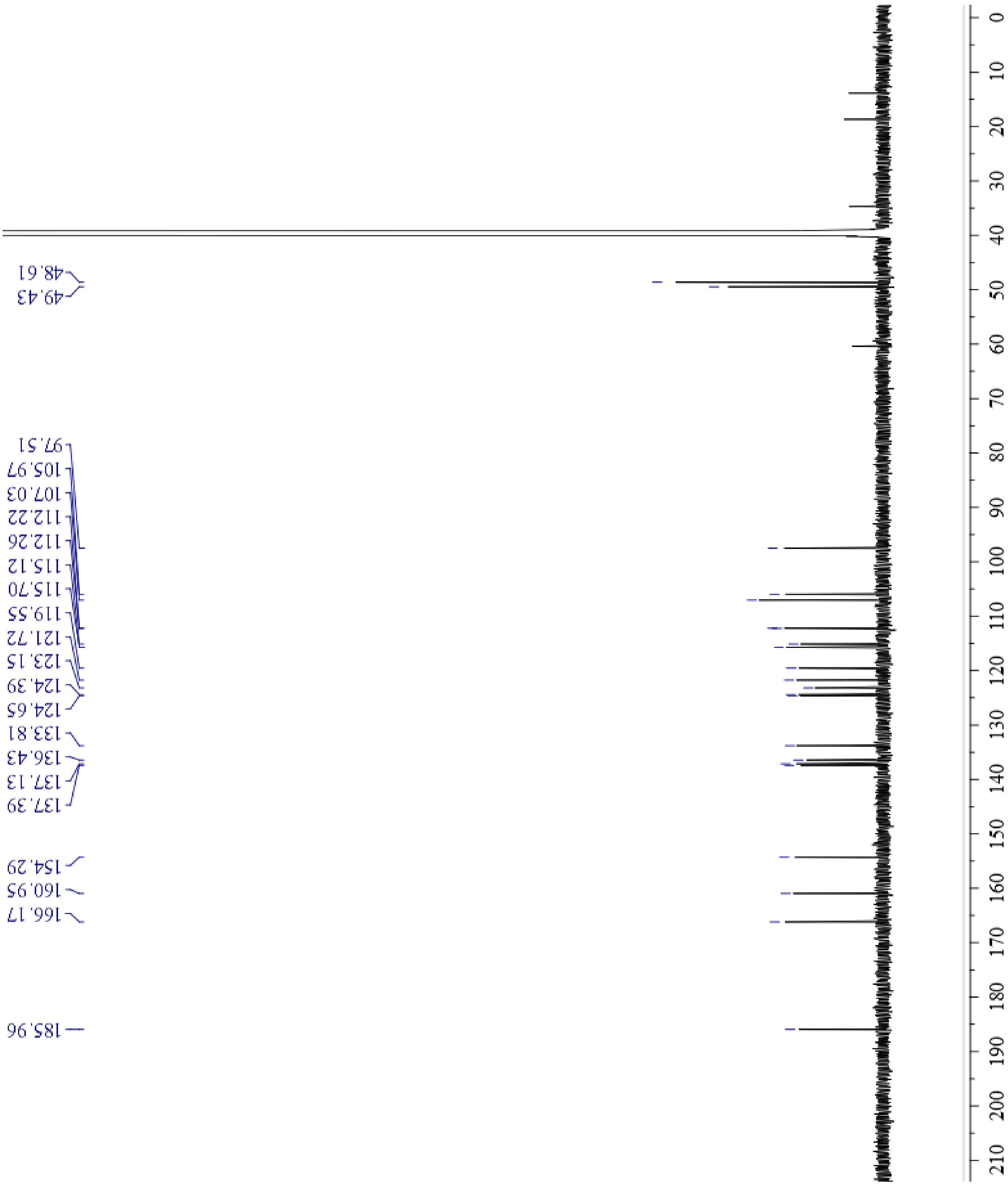

Figure S22. The ^{13}C NMR (200 MHz, DMSO-*d6*) spectrum of **4**

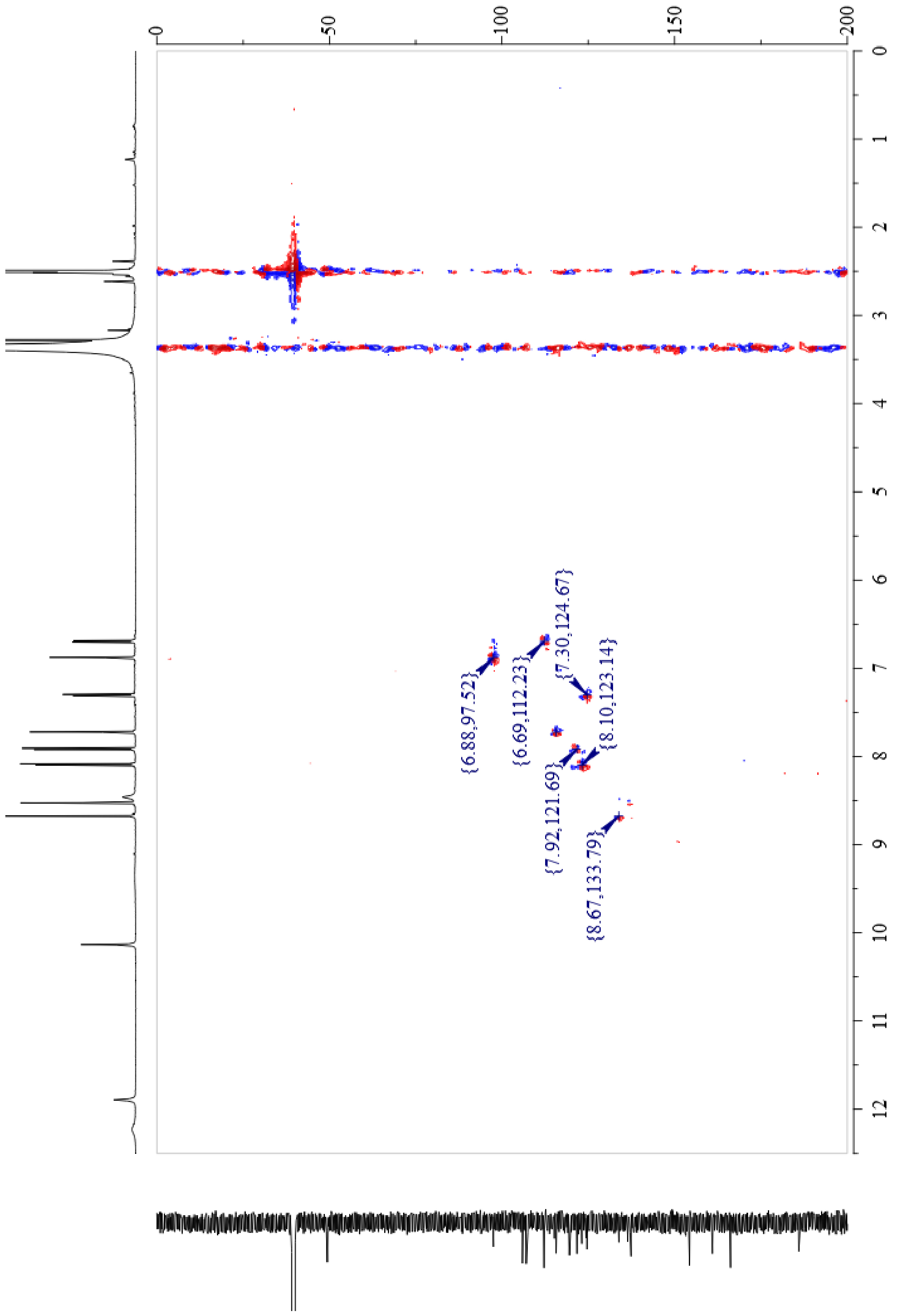

Figure S23. The HSQC (800 MHz, DMSO-*d6*) spectrum of **4**

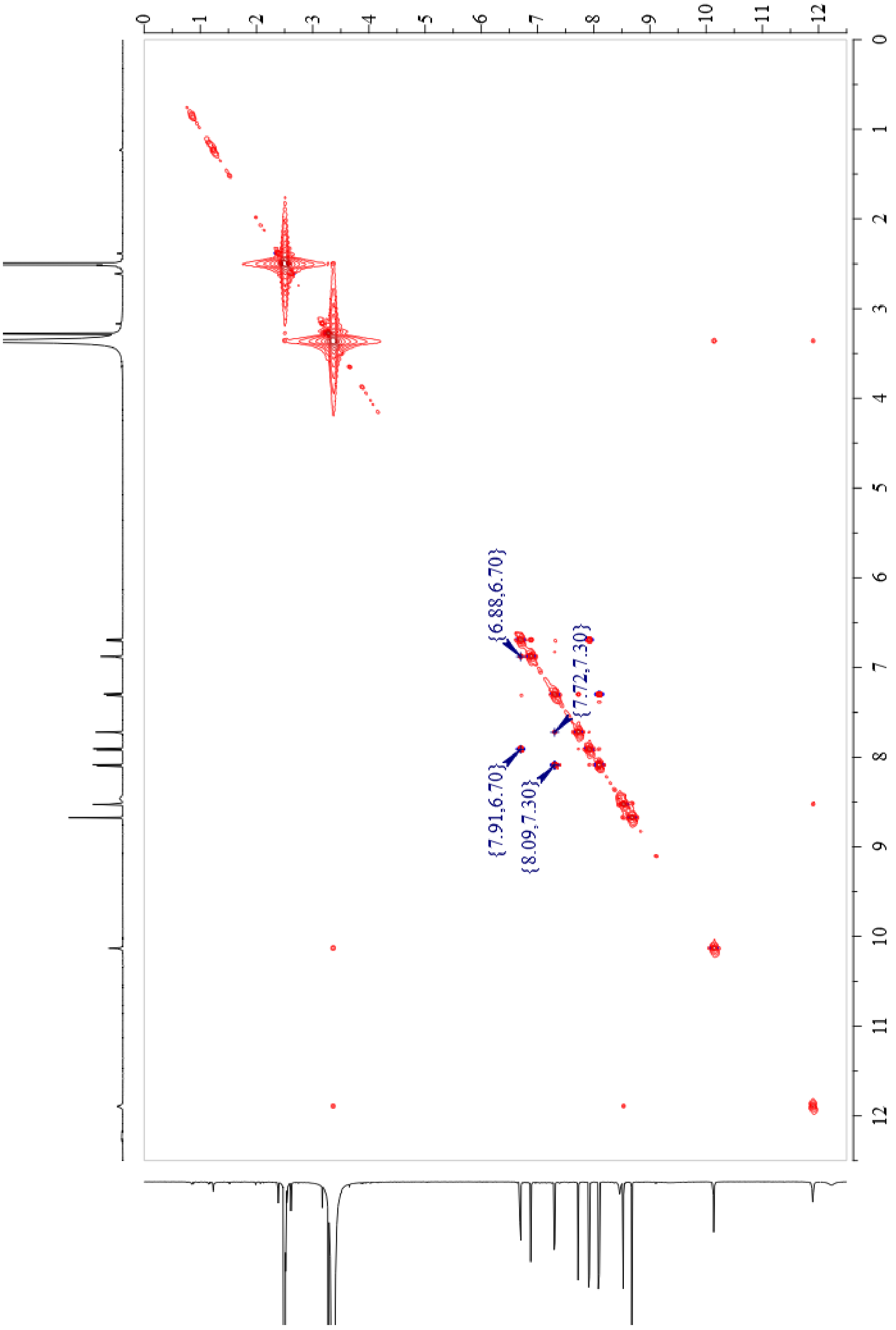

Figure S24. The COSY (800 MHz, DMSO-*d6*) spectrum of **4**

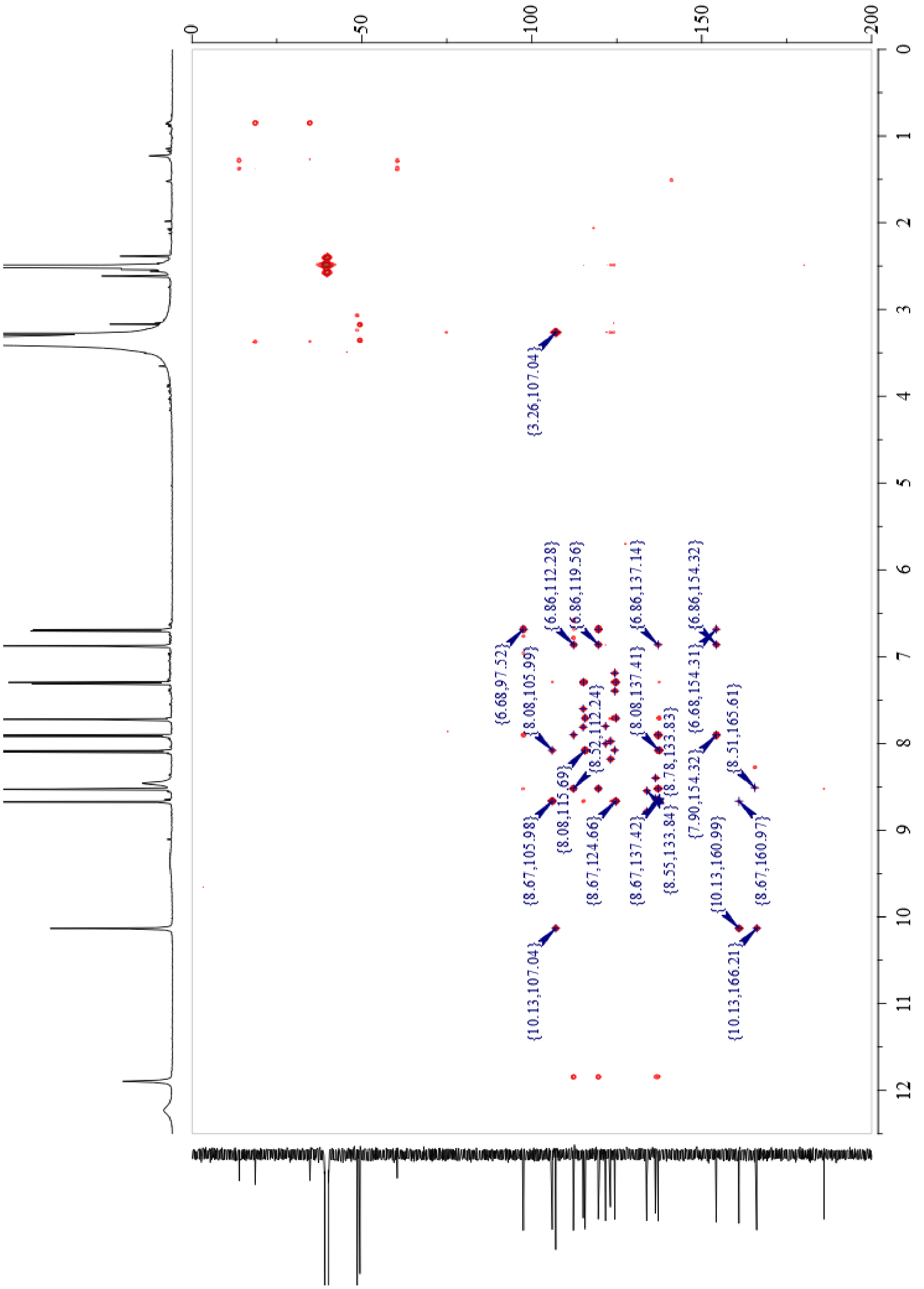

Figure S25. The HMBC (800 MHz, DMSO-*d6*) spectrum of **4**

Observed m/z Int%
467.0351 2.2

Err[ppm / mmu]	U.S.	Composition
-18.0 / -8.4	23.5	C 31 H 16 79Br
+19.9 / +9.3	24.5	C 31 H 14 81Br
+8.9 / +4.2	24.0	C 30 H 14 79Br N
+1.7 / +0.8	9.5	C 21 H 25 81Br 2 N 2
-9.3 / -4.3	9.0	C 20 H 25 79Br 81Br N 3
-1.4 / -0.6	35.5	C 34 H 3 N 4
+17.6 / +8.2	9.5	C 19 H 23 79Br 81Br N 4
+6.7 / +3.1	9.0	C 18 H 23 79Br 2 N 5
-19.6 / -9.2	20.5	C 24 H 14 81Br N 6
+7.3 / +3.4	21.0	C 23 H 12 81Br N 7
-3.6 / -1.7	20.5	C 22 H 12 79Br N 8
-10.9 / -5.1	6.0	C 13 H 23 81Br 2 N 9
+16.0 / +7.5	6.5	C 12 H 21 81Br 2 N 10
-12.2 / -5.7	8.5	C 22 H 27 79Br 81Br O
-4.3 / -2.0	35.0	C 36 H 5 N O
+14.7 / +6.9	9.0	C 21 H 25 79Br 81Br N O
+3.8 / +1.8	8.5	C 20 H 25 79Br 2 N 2 O
+4.4 / +2.1	20.5	C 25 H 14 81Br N 4 O
-6.5 / -3.0	20.0	C 24 H 14 79Br N 5 O
-13.8 / -6.4	5.5	C 15 H 25 81Br 2 N 6 O
+13.1 / +6.1	6.0	C 14 H 23 81Br 2 N 7 O
-16.8 / -7.9	31.5	C 28 H 3 N 8 O
+2.2 / +1.0	5.5	C 13 H 23 79Br 81Br N 8 O
+10.1 / +4.7	32.0	C 27 H N 9 O
-8.8 / -4.1	5.0	C 12 H 23 79Br 2 N 9 O
+18.1 / +8.5	5.5	C 11 H 21 79Br 2 N 10 O
+1.6 / +0.7	20.0	C 27 H 16 81Br N O 2
-9.4 / -4.4	19.5	C 26 H 16 79Br N 2 O 2
+17.5 / +8.2	20.0	C 25 H 14 79Br N 3 O 2
-16.7 / -7.8	5.0	C 17 H 27 81Br 2 N 3 O 2
+10.3 / +4.8	5.5	C 16 H 25 81Br 2 N 4 O 2
-19.7 / -9.2	31.0	C 30 H 5 N 5 O 2
-0.7 / -0.3	5.0	C 15 H 25 79Br 81Br N 5 O 2
+7.2 / +3.4	31.5	C 29 H 3 N 6 O 2
-11.7 / -5.4	4.5	C 14 H 25 79Br 2 N 6 O 2
+15.3 / +7.1	5.0	C 13 H 23 79Br 2 N 7 O 2
-11.0 / -5.1	16.5	C 19 H 14 81Br N 8 O 2
+15.9 / +7.4	17.0	C 18 H 12 81Br N 9 O 2
+5.0 / +2.3	16.5	C 17 H 12 79Br N 10 O 2
+14.7 / +6.8	19.5	C 27 H 16 79Br O 3
-19.5 / -9.1	4.5	C 19 H 29 81Br 2 O 3
+7.4 / +3.5	5.0	C 18 H 27 81Br 2 N O 3
-3.6 / -1.7	4.5	C 17 H 27 79Br 81Br N 2 O 3
+4.4 / +2.0	31.0	C 31 H 5 N 3 O 3
-14.5 / -6.8	4.0	C 16 H 27 79Br 2 N 3 O 3
+12.4 / +5.8	4.5	C 15 H 25 79Br 2 N 4 O 3
-13.9 / -6.5	16.0	C 21 H 16 81Br N 5 O 3
+13.1 / +6.1	16.5	C 20 H 14 81Br N 6 O 3
+2.1 / +1.0	16.0	C 19 H 14 79Br N 7 O 3
-5.2 / -2.4	1.5	C 10 H 25 81Br 2 N 8 O 3
-16.1 / -7.5	1.0	C 9 H 25 79Br 81Br N 9 O 3
-8.2 / -3.8	27.5	C 23 H 3 N 10 O 3
+10.8 / +5.0	1.5	C 8 H 23 79Br 81Br N 10 O 3
+1.5 / +0.7	30.5	C 33 H 7 O 4
-17.4 / -8.1	3.5	C 18 H 29 79Br 2 O 4
+9.5 / +4.4	4.0	C 17 H 27 79Br 2 N O 4
-16.7 / -7.8	15.5	C 23 H 18 81Br N 2 O 4
+10.2 / +4.8	16.0	C 22 H 16 81Br N 3 O 4
-0.8 / -0.4	15.5	C 21 H 16 79Br N 4 O 4
-8.0 / -3.8	1.0	C 12 H 27 81Br 2 N 5 O 4
-19.0 / -8.9	0.5	C 11 H 27 79Br 81Br N 6 O 4
+18.9 / +8.8	1.5	C 11 H 25 81Br 2 N 6 O 4
-11.1 / -5.2	27.0	C 25 H 5 N 7 O 4
+7.9 / +3.7	1.0	C 10 H 25 79Br 81Br N 7 O 4
+15.8 / +7.4	27.5	C 24 H 3 N 8 O 4
-3.0 / -1.4	0.5	C 9 H 25 79Br 2 N 8 O 4
-2.4 / -1.1	12.5	C 14 H 14 81Br N 10 O 4
+7.3 / +3.4	15.5	C 24 H 18 81Br O 5
-3.7 / -1.7	15.0	C 23 H 18 79Br N O 5
-10.9 / -5.1	0.5	C 14 H 29 81Br 2 N 2 O 5
+16.0 / +7.5	1.0	C 13 H 27 81Br 2 N 3 O 5
-14.0 / -6.5	26.5	C 27 H 7 N 4 O 5
+5.0 / +2.4	0.5	C 12 H 27 79Br 81Br N 4 O 5
+13.0 / +6.1	27.0	C 26 H 5 N 5 O 5
-5.9 / -2.8	0.0	C 11 H 27 79Br 2 N 5 O 5
-5.3 / -2.5	12.0	C 16 H 16 81Br N 7 O 5
-16.2 / -7.6	11.5	C 15 H 16 79Br N 8 O 5
+10.7 / +5.0	12.0	C 14 H 14 79Br N 9 O 5
+13.1 / +6.1	0.5	C 15 H 29 81Br 2 O 6
-16.8 / -7.9	26.0	C 29 H 9 N O 6
+2.2 / +1.0	0.0	C 14 H 29 79Br 81Br N O 6
+10.1 / +4.7	26.5	C 28 H 7 N 2 O 6
-8.8 / -4.1	-0.5	C 13 H 29 79Br 2 N 2 O 6
+18.1 / +8.5	0.0	C 12 H 27 79Br 2 N 3 O 6
-8.1 / -3.8	11.5	C 18 H 18 81Br N 4 O 6
-19.1 / -8.9	11.0	C 17 H 18 79Br N 5 O 6
+18.8 / +8.8	12.0	C 17 H 16 81Br N 5 O 6
+7.8 / +3.7	11.5	C 16 H 16 79Br N 6 O 6
-2.5 / -1.2	23.0	C 20 H 5 N 9 O 6
+15.3 / +7.1	-0.5	C 14 H 29 79Br 2 O 7
-11.0 / -5.1	11.0	C 20 H 20 81Br N O 7
+15.9 / +7.4	11.5	C 19 H 18 81Br N 2 O 7
+5.0 / +2.3	11.0	C 18 H 18 79Br N 3 O 7
-5.3 / -2.5	22.5	C 22 H 7 N 6 O 7
+3.4 / +1.6	8.0	C 11 H 16 81Br N 9 O 7
-7.6 / -3.6	7.5	C 10 H 16 79Br N 10 O 7
+2.1 / +1.0	10.5	C 20 H 20 79Br O 8
-8.2 / -3.8	22.0	C 24 H 9 N 3 O 8
+18.7 / +8.7	22.5	C 23 H 7 N 4 O 8
+0.5 / +0.2	7.5	C 13 H 18 81Br N 6 O 8
-10.5 / -4.9	7.0	C 12 H 18 79Br N 7 O 8
+16.4 / +7.7	7.5	C 11 H 16 79Br N 8 O 8
-11.1 / -5.2	21.5	C 26 H 11 O 9
+15.8 / +7.4	22.0	C 25 H 9 N O 9
-2.4 / -1.1	7.0	C 15 H 20 81Br N 3 O 9
-13.4 / -6.2	6.5	C 14 H 20 79Br N 4 O 9
+13.6 / +6.3	7.0	C 13 H 18 79Br N 5 O 9
+3.3 / +1.5	18.5	C 17 H 7 N 8 O 9
-15.0 / -7.0	3.5	C 7 H 18 81Br N 10 O 9
-5.3 / -2.5	6.5	C 17 H 22 81Br O 10
-16.2 / -7.6	6.0	C 16 H 22 79Br N O 10
+10.7 / +5.0	6.5	C 15 H 20 79Br N 2 O 10
+0.4 / +0.2	18.0	C 19 H 9 N 5 O 10
-17.8 / -8.3	3.0	C 9 H 20 81Br N 7 O 10
+9.1 / +4.2	3.5	C 8 H 18 81Br N 8 O 10
-1.9 / -0.9	3.0	C 7 H 18 79Br N 9 O 10

[Theoretical Ion Distribution]

Page: 1

Molecular Formula : C21 H16 Br N4 O4
(m/z 467.0355, MW 468.2864, U.S. 15.5)
Base Peak : 469.0337, Averaged MW : 468.2846(a), 468.2873(w)

m/z	INT.	
467.0355	98.9447	***
468.0385	24.7151	***************
469.0337	100.0000	**
470.0366	24.4653	***************
471.0391	3.6852	**
472.0416	0.4145	
473.0441	0.0374	
474.0465	0.0028	
475.0490	0.0002	

Figure S26. The HRFABMS data of **4**

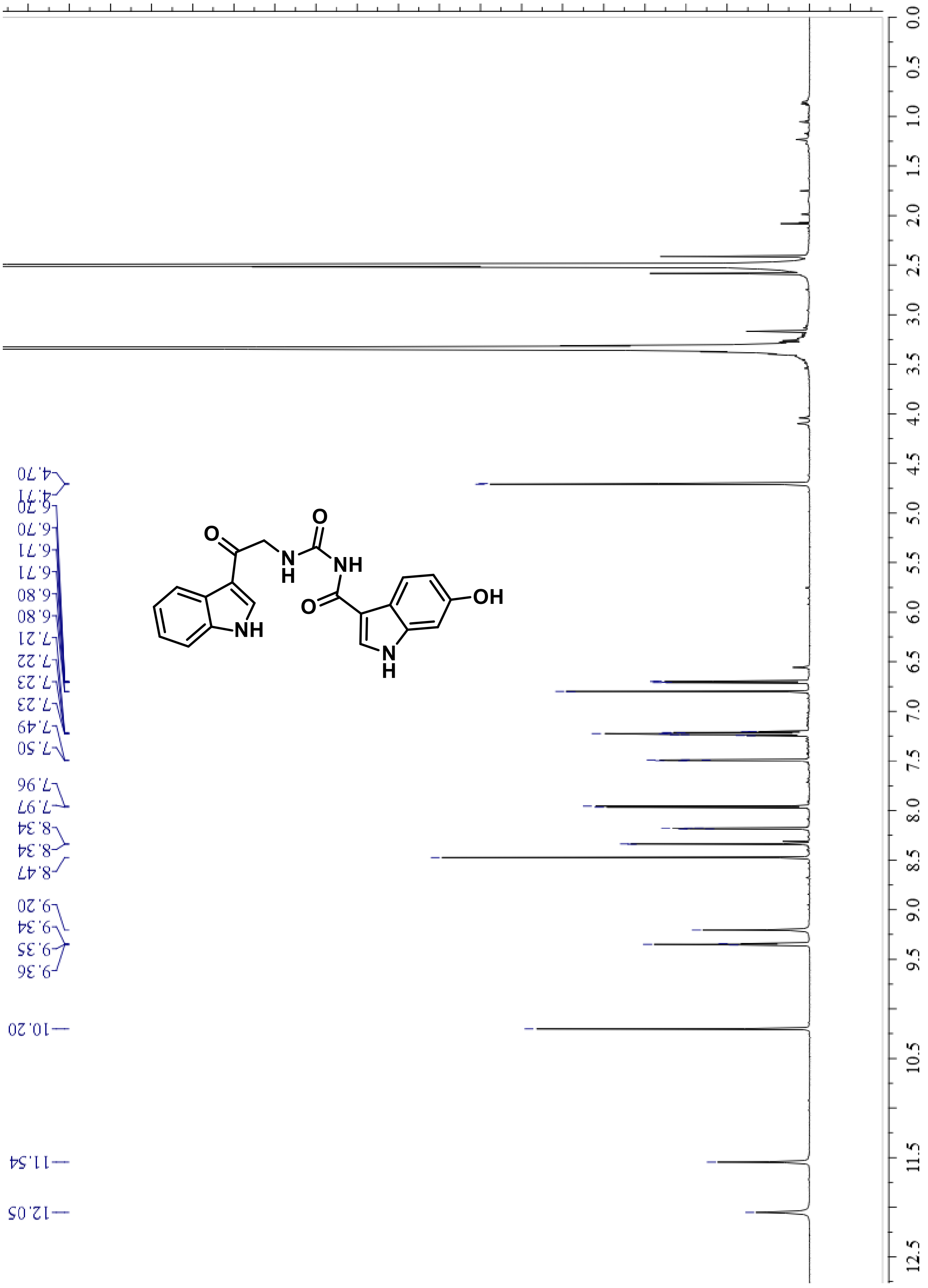

Figure S27. The ^{1}H NMR (800 MHz, DMSO-*d6*) spectrum of **5**

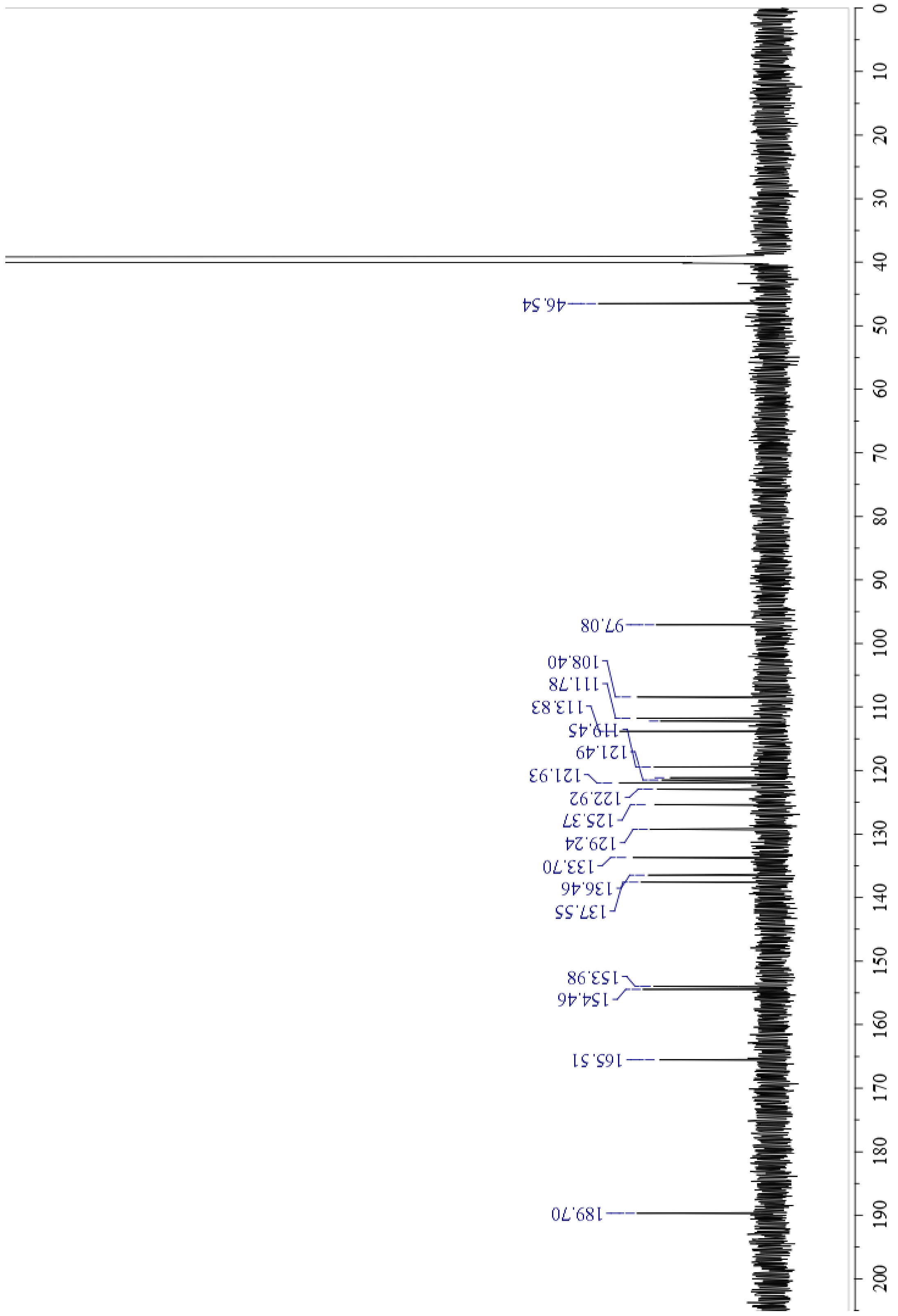

Figure S28. The ^{13}C NMR (200 MHz, DMSO-*d6*) spectrum of **5**

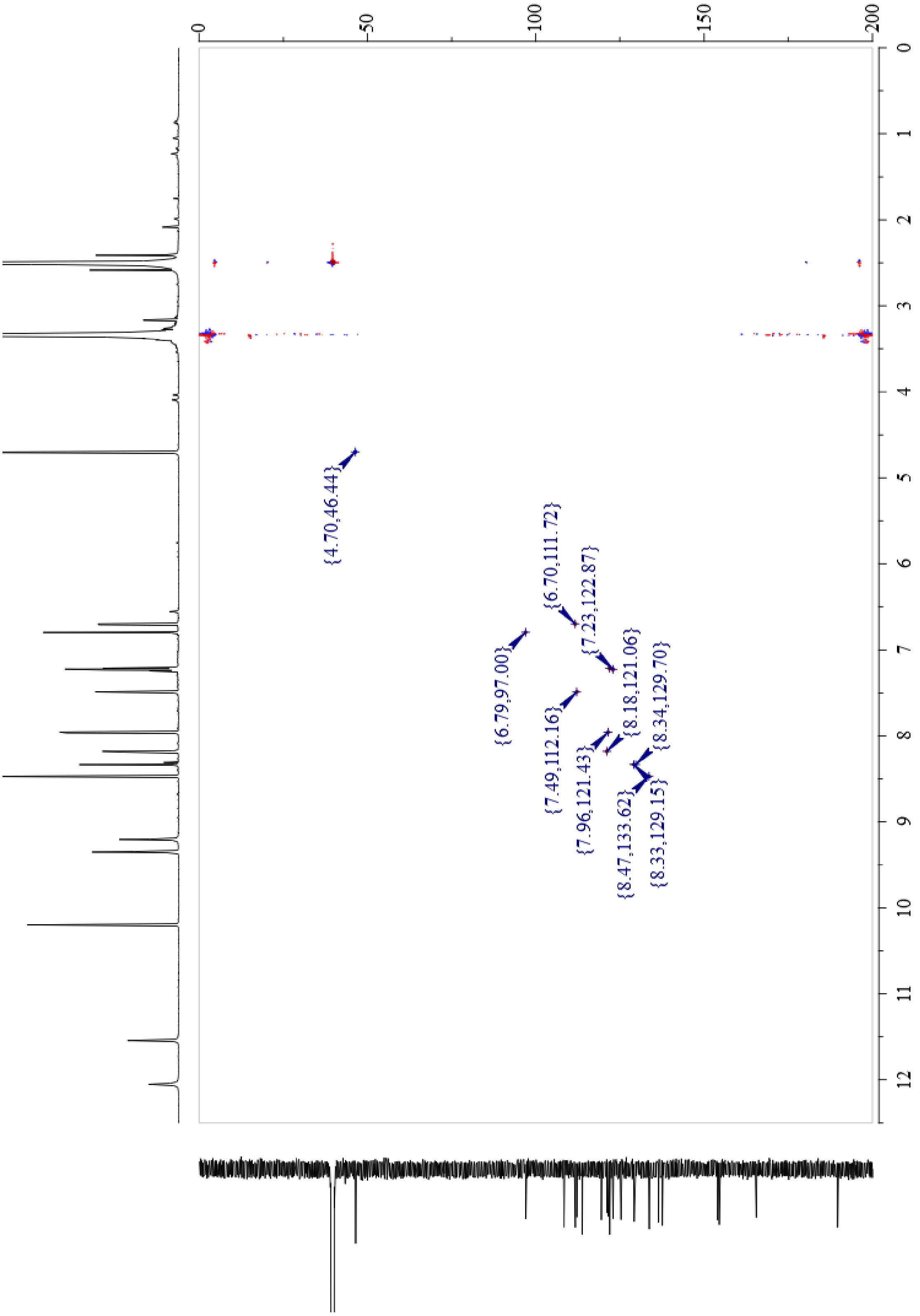

Figure S29. The HSQC (800 MHz, DMSO-*d6*) spectrum of **5**

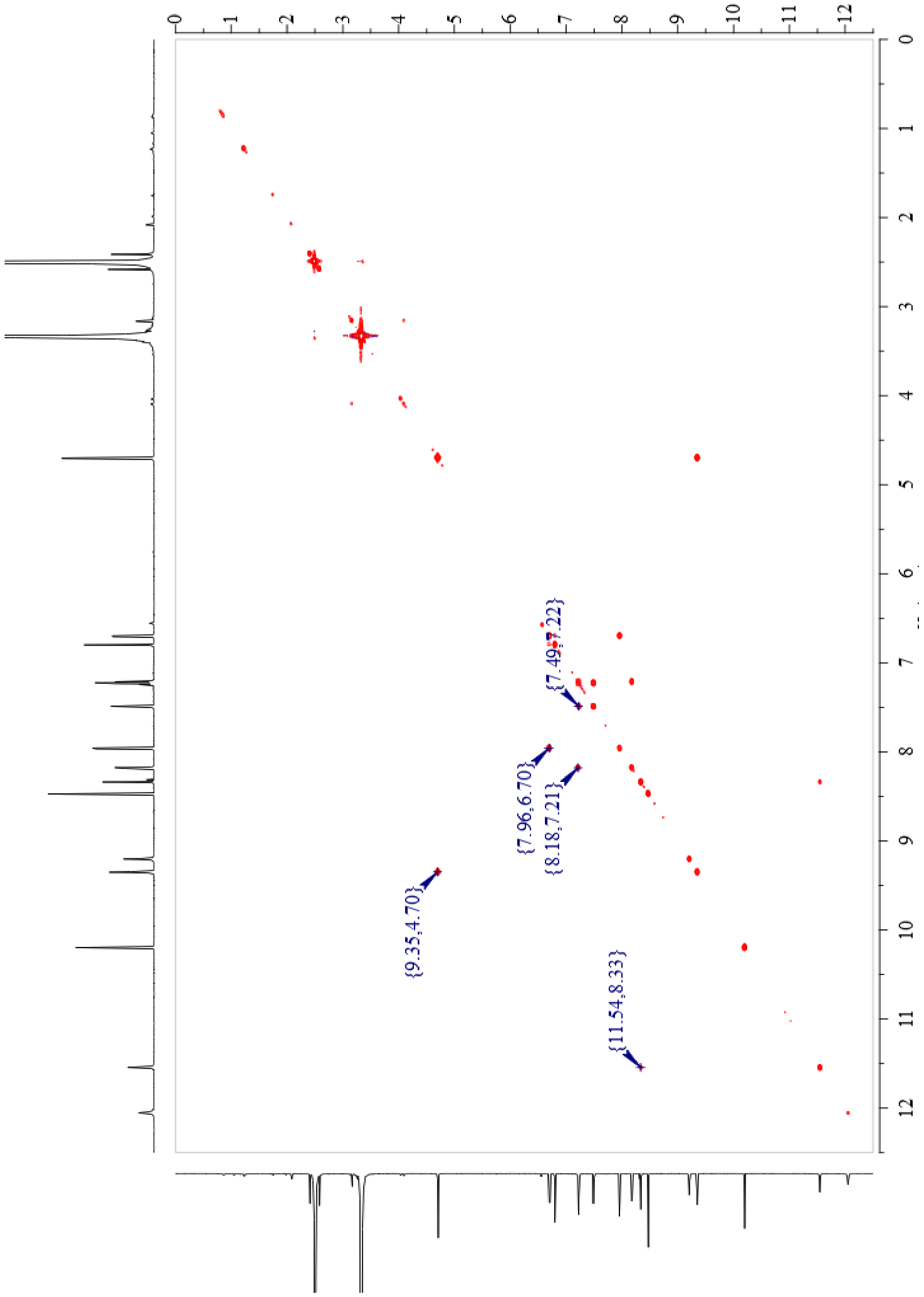

Figure S30. The COSY (800 MHz, DMSO-*d6*) spectrum of **5**

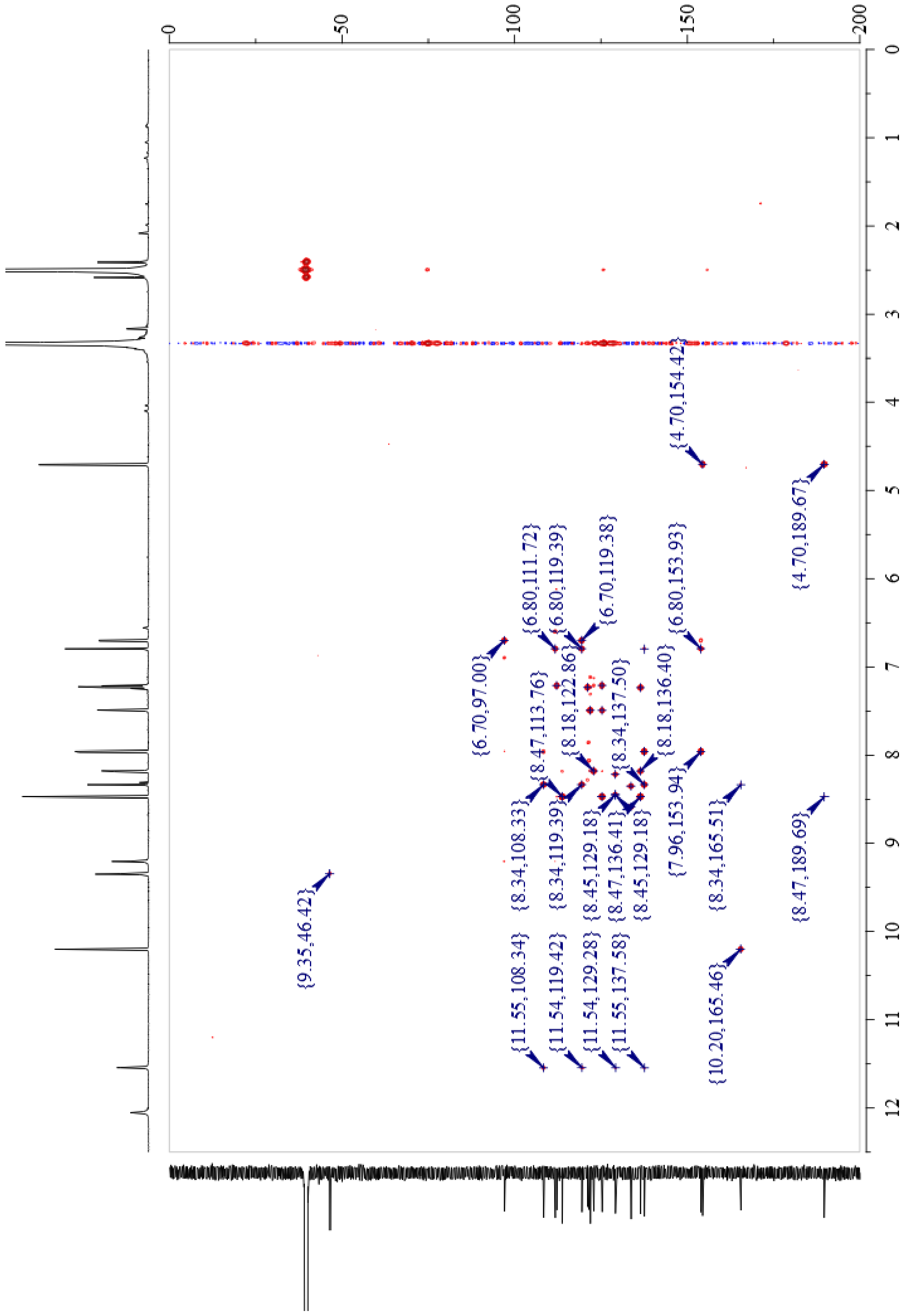

Figure S31. The HMBC (800 MHz, DMSO-*d6*) spectrum of **5**

Observed m/z	Int%	Err[ppm / mmu]	U.S.	Composition
377.1249	14.3	+11.8 / +4.5	23.0	C 29 H 15 N
		-3.7 / -1.4	19.5	C 21 H 13 N 8
		-7.3 / -2.8	19.0	C 23 H 15 N 5 O
		-10.9 / -4.1	18.5	C 25 H 17 N 2 O 2
		+6.9 / +2.6	15.5	C 16 H 13 N 10 O 2
		+18.9 / +7.1	18.5	C 26 H 17 O 3
		+3.4 / +1.3	15.0	C 18 H 15 N 7 O 3
		-0.2 / -0.1	14.5	C 20 H 17 N 4 O 4
		-3.8 / -1.4	14.0	C 22 H 19 N O 5
		-19.3 / -7.3	10.5	C 14 H 17 N 8 O 5
		+14.0 / +5.3	11.0	C 13 H 15 N 9 O 5
		+10.5 / +4.0	10.5	C 15 H 17 N 6 O 6
		+6.9 / +2.6	10.0	C 17 H 19 N 3 O 7
		-8.6 / -3.3	6.5	C 9 H 17 N 10 O 7
		+3.4 / +1.3	9.5	C 19 H 21 O 8
		-12.2 / -4.6	6.0	C 11 H 19 N 7 O 8
		-15.8 / -5.9	5.5	C 13 H 21 N 4 O 9
		+17.6 / +6.6	6.0	C 12 H 19 N 5 O 9
		-19.3 / -7.3	5.0	C 15 H 23 N O 10
		+14.0 / +5.3	5.5	C 14 H 21 N 2 O 10
		-1.5 / -0.6	2.0	C 6 H 19 N 9 O 10

[Theoretical Ion Distribution]   Page: 1
Molecular Formula : C20 H17 N4 O4
(m/z 377.1250, MW 377.3794, U.S. 14.5)
Base Peak : 377.1250, Averaged MW : 377.3779(a), 377.3786(w)

m/z	INT.	
377.1250	100.0000	**
378.1280	23.8664	**************
379.1305	3.5235	**
380.1330	0.3884	
381.1355	0.0344	
382.1379	0.0025	
383.1404	0.0002	

Figure S32. The HRFABMS data of **5**

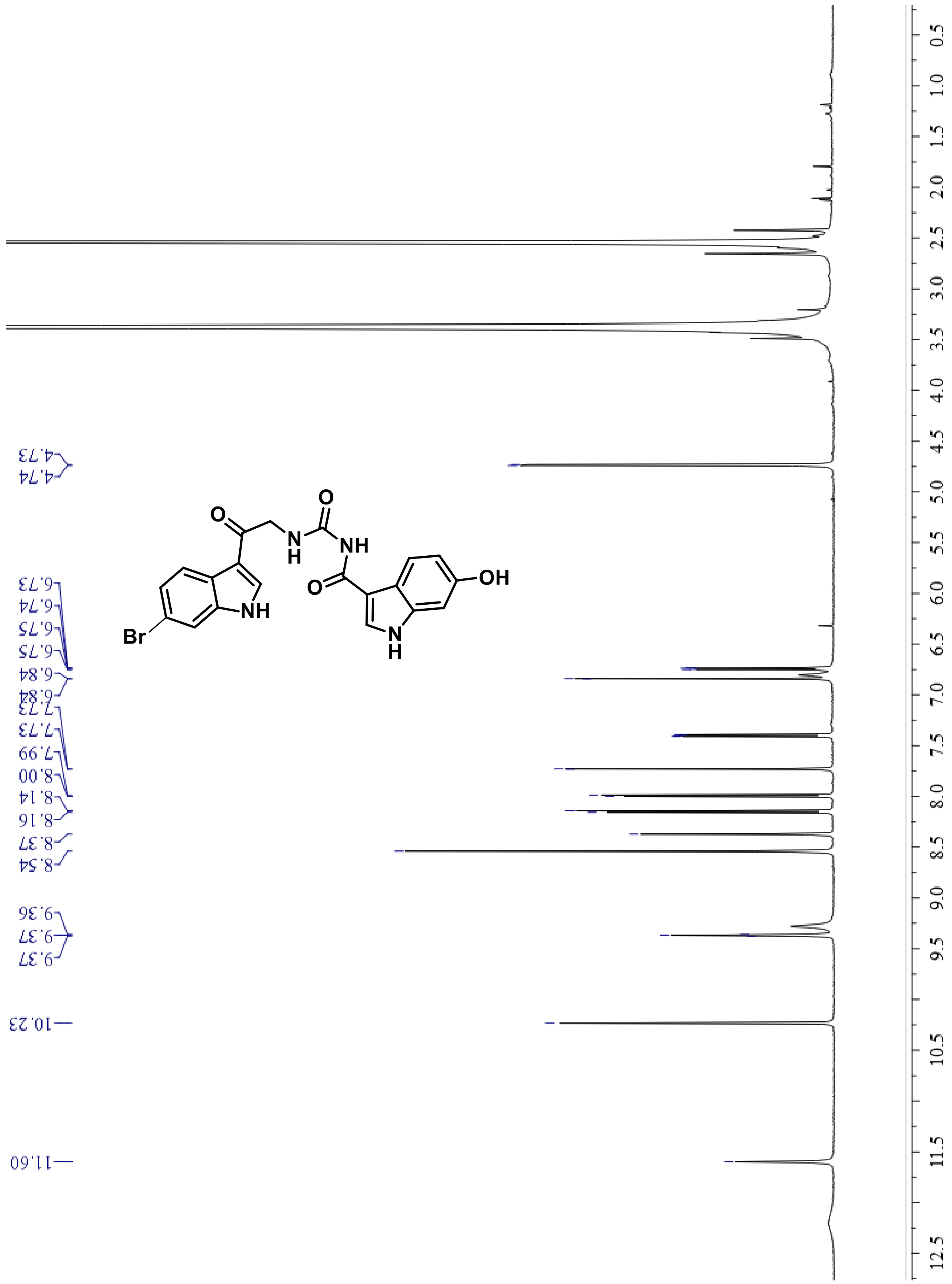

Figure S33. The ^{1}H NMR (800 MHz, DMSO-*d6*) spectrum of **6**

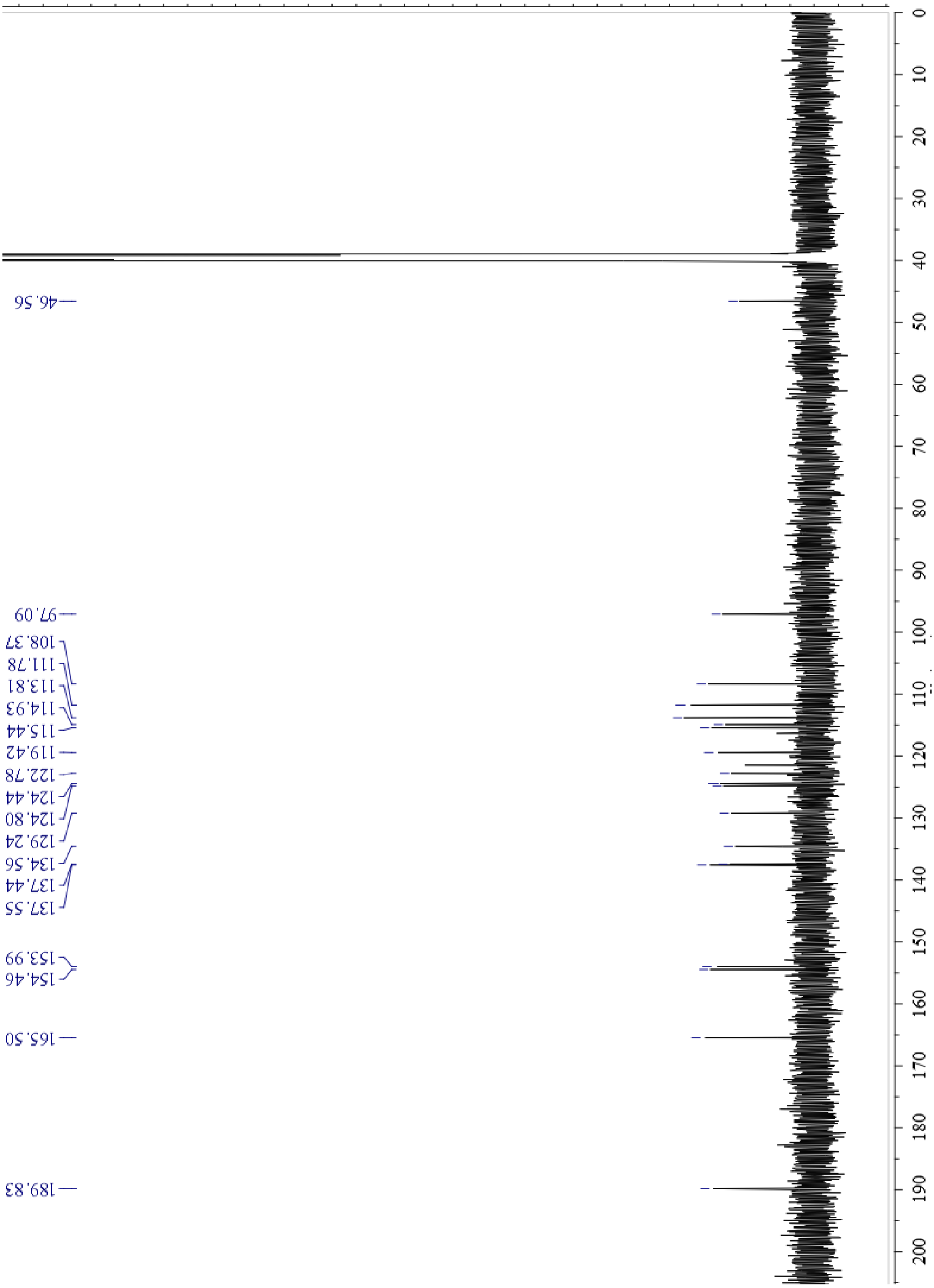

Figure S34. The ^{13}C NMR (200 MHz, DMSO-*d6*) spectrum of **6**

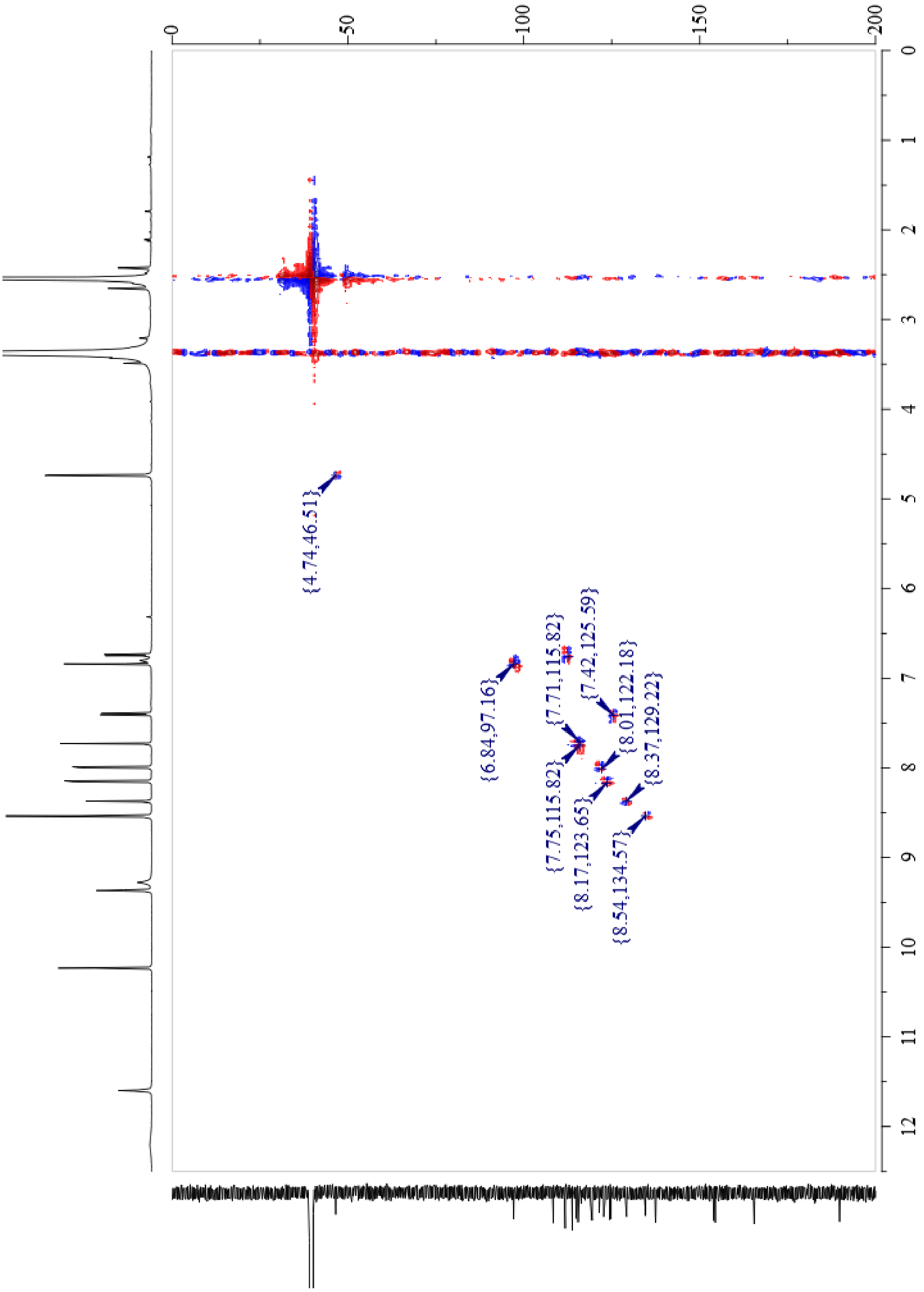

Figure S35. The HSQC (800 MHz, DMSO-*d6*) spectrum of **6**

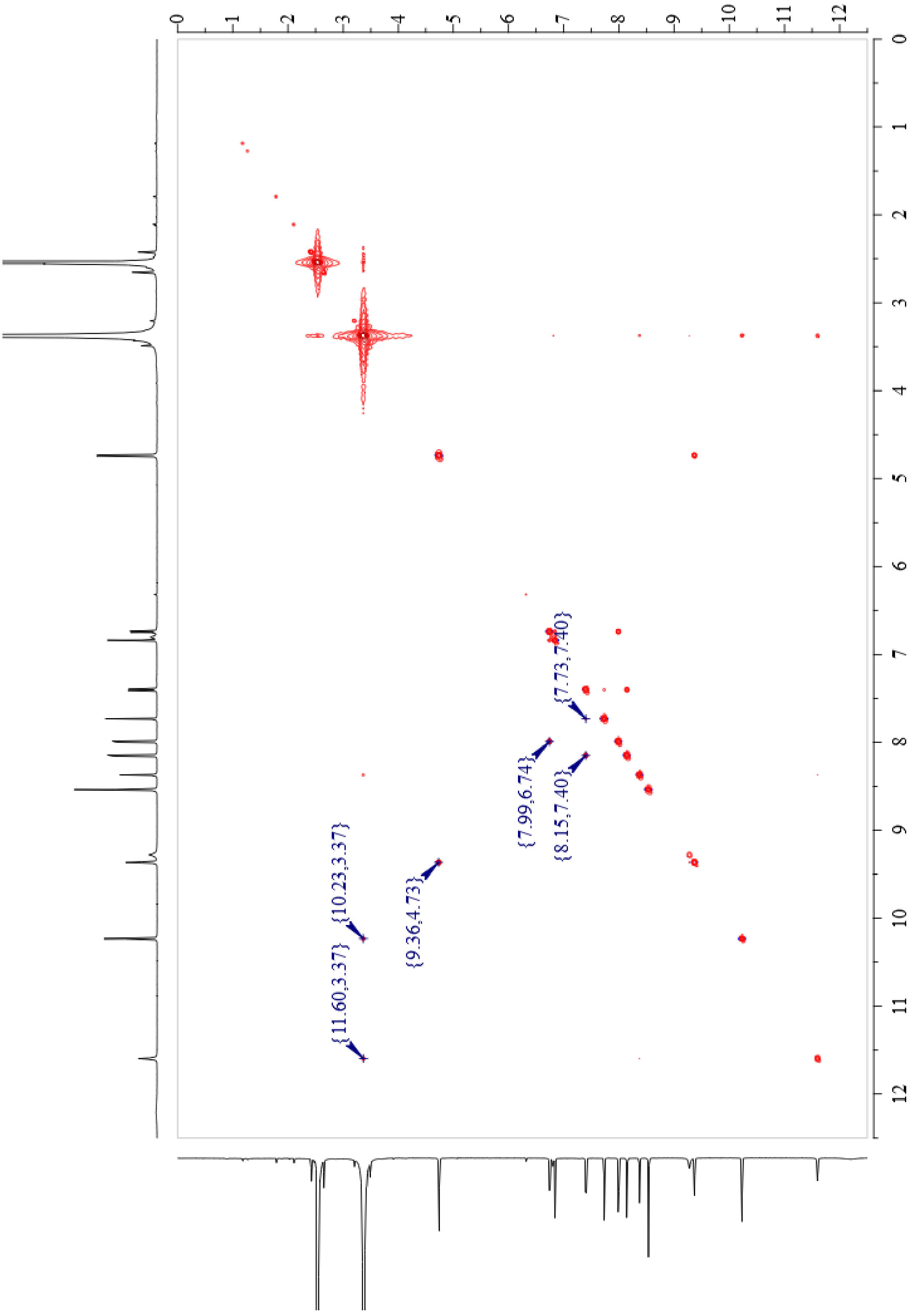

Figure S36. The COSY (800 MHz, DMSO-*d6*) spectrum of **6**

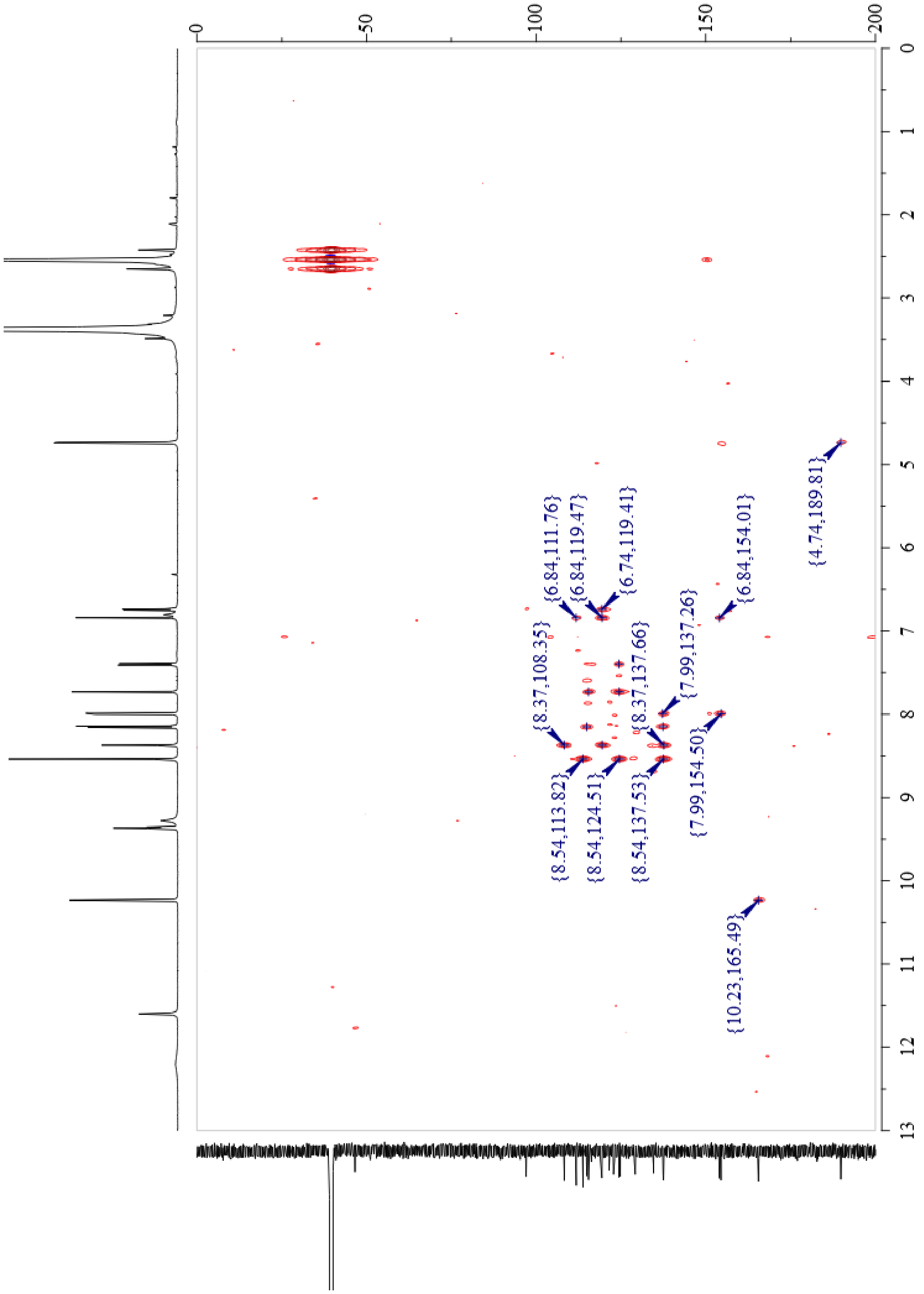

Figure S37. The HMBC (800 MHz, DMSO-*d6*) spectrum of **6**

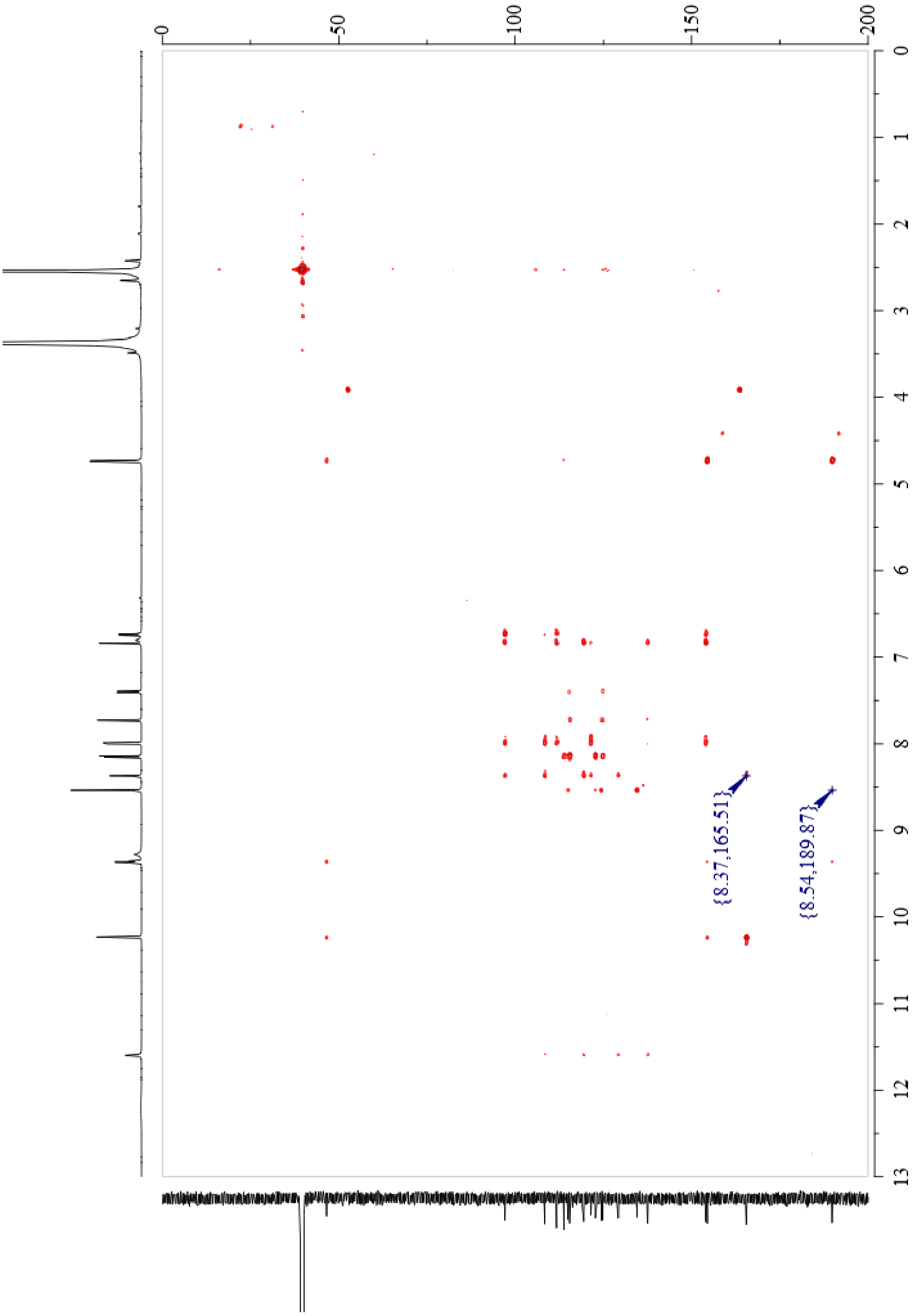

Figure S38. The dHMBC at 4Hz (800 MHz, DMSO-*d6*) spectrum of **6**

Observed m/z Int%
455.0356 0.6

Err[ppm / mmu]	U.S.	Composition
-17.5 / -8.0	22.5	C 30 H 16 79Br
+10.1 / +4.6	23.0	C 29 H 14 79Br N
+2.6 / +1.2	8.5	C 20 H 25 81Br 2 N 2
-8.6 / -3.9	8.0	C 19 H 25 79Br 81Br N 3
-0.5 / -0.2	34.5	C 33 H 3 N 4
-19.9 / -9.0	7.5	C 18 H 25 79Br 2 N 4
+19.0 / +8.7	8.5	C 18 H 23 79Br 81Br N 4
+7.8 / +3.5	8.0	C 17 H 23 79Br 2 N 5
-19.2 / -8.7	19.5	C 23 H 14 81Br N 6
+8.4 / +3.8	20.0	C 22 H 12 81Br N 7
-2.8 / -1.3	19.5	C 21 H 12 79Br N 8
-10.3 / -4.7	5.0	C 12 H 23 81Br 2 N 9
+17.4 / +7.9	5.5	C 11 H 21 81Br 2 N 10
-11.6 / -5.3	7.5	C 21 H 27 79Br 81Br O
-3.4 / -1.6	34.0	C 35 H 5 N O
+16.1 / +7.3	8.0	C 20 H 25 79Br 81Br N O
+4.8 / +2.2	7.5	C 19 H 25 79Br 2 N 2 O
+5.5 / +2.5	19.5	C 24 H 14 81Br N 4 O
-5.8 / -2.6	19.0	C 23 H 14 79Br N 5 O
-13.2 / -6.0	4.5	C 14 H 25 81Br 2 N 6 O
+14.4 / +6.6	5.0	C 13 H 23 81Br 2 N 7 O
-16.3 / -7.4	30.5	C 27 H 3 N 8 O
+3.2 / +1.4	4.5	C 12 H 23 79Br 81Br N 8 O
+11.3 / +5.1	31.0	C 26 H N 9 O
-8.1 / -3.7	4.0	C 11 H 23 79Br 2 N 9 O
+19.6 / +8.9	4.5	C 10 H 21 79Br 2 N 10 O
+2.5 / +1.2	19.0	C 26 H 16 81Br N O 2
-8.7 / -4.0	18.5	C 25 H 16 79Br N 2 O 2
+18.9 / +8.6	19.0	C 24 H 14 79Br N 3 O 2
-16.2 / -7.4	4.0	C 16 H 27 81Br 2 N 3 O 2
+11.5 / +5.2	4.5	C 15 H 25 81Br 2 N 4 O 2
-19.3 / -8.8	30.0	C 29 H 5 N 5 O 2
+0.2 / +0.1	4.0	C 14 H 25 79Br 81Br N 5 O 2
+8.4 / +3.8	30.5	C 28 H 3 N 6 O 2
-11.0 / -5.0	3.5	C 13 H 25 79Br 2 N 6 O 2
+16.6 / +7.6	4.0	C 12 H 23 79Br 2 N 7 O 2
-10.3 / -4.7	15.5	C 18 H 14 81Br N 8 O 2
+17.3 / +7.9	16.0	C 17 H 12 81Br N 9 O 2
+6.0 / +2.7	15.5	C 16 H 12 79Br N 10 O 2
+16.0 / +7.3	18.5	C 26 H 16 79Br O 3
-19.1 / -8.7	3.5	C 18 H 29 81Br 2 O 3
+8.5 / +3.9	4.0	C 17 H 27 81Br 2 N O 3
-2.7 / -1.2	3.5	C 16 H 27 79Br 81Br N 2 O 3
+5.4 / +2.5	30.0	C 30 H 5 N 3 O 3
-14.0 / -6.4	3.0	C 15 H 27 79Br 2 N 3 O 3
+13.7 / +6.2	3.5	C 14 H 25 79Br 2 N 4 O 3
-13.3 / -6.1	15.0	C 20 H 16 81Br N 5 O 3
+14.3 / +6.5	15.5	C 19 H 14 81Br N 6 O 3
+3.1 / +1.4	15.0	C 18 H 14 79Br N 7 O 3
-4.4 / -2.0	0.5	C 9 H 25 81Br 2 N 8 O 3
-15.6 / -7.1	0.0	C 8 H 25 79Br 81Br N 9 O 3
-7.5 / -3.4	26.5	C 22 H 3 N 10 O 3
+12.0 / +5.5	0.5	C 7 H 23 79Br 81Br N 10 O 3
+2.5 / +1.1	29.5	C 32 H 7 O 4
-16.9 / -7.7	2.5	C 17 H 29 79Br 2 O 4

+10.7 / +4.9	3.0	C 16 H 27 79Br 2 N O 4
-16.2 / -7.4	14.5	C 22 H 18 81Br N 2 O 4
+11.4 / +5.2	15.0	C 21 H 16 81Br N 3 O 4
+0.1 / +0.1	14.5	C 20 H 16 79Br N 4 O 4
-7.3 / -3.3	0.0	C 11 H 27 81Br 2 N 5 O 4
-18.6 / -8.5	-0.5	C 10 H 27 79Br 81Br N 6 O
-10.4 / -4.7	26.0	C 24 H 5 N 7 O 4
+9.1 / +4.1	0.0	C 9 H 25 79Br 81Br N 7 O
+17.2 / +7.8	26.5	C 23 H 3 N 8 O 4
-2.2 / -1.0	-0.5	C 8 H 25 79Br 2 N 8 O 4
-1.5 / -0.7	11.5	C 13 H 14 81Br N 10 O 4
+8.4 / +3.8	14.5	C 23 H 18 81Br O 5
-2.8 / -1.3	14.0	C 22 H 18 79Br N O 5
-10.3 / -4.7	-0.5	C 13 H 29 81Br 2 N 2 O 5
+17.4 / +7.9	0.0	C 12 H 27 81Br 2 N 3 O 5
-13.4 / -6.1	25.5	C 26 H 7 N 4 O 5
+6.1 / +2.8	-0.5	C 11 H 27 79Br 81Br N 4 O
+14.3 / +6.5	26.0	C 25 H 5 N 5 O 5
-4.5 / -2.0	11.0	C 15 H 16 81Br N 7 O 5
-15.7 / -7.1	10.5	C 14 H 16 79Br N 8 O 5
+11.9 / +5.4	11.0	C 13 H 14 79Br N 9 O 5
+14.4 / +6.6	-0.5	C 14 H 29 81Br 2 O 6
-16.3 / -7.4	25.0	C 28 H 9 N O 6
+11.3 / +5.1	25.5	C 27 H 7 N 2 O 6
-7.4 / -3.4	10.5	C 17 H 18 81Br N 4 O 6
-18.7 / -8.5	10.0	C 16 H 18 79Br N 5 O 6
+9.0 / +4.1	10.5	C 15 H 16 79Br N 6 O 6
-1.6 / -0.7	22.0	C 19 H 5 N 9 O 6
-10.4 / -4.7	10.0	C 19 H 20 81Br N O 7
+17.3 / +7.9	10.5	C 18 H 18 81Br N 2 O 7
+6.0 / +2.7	10.0	C 17 H 18 79Br N 3 O 7
-4.5 / -2.1	21.5	C 21 H 7 N 6 O 7
+4.4 / +2.0	7.0	C 10 H 16 81Br N 9 O 7
-6.9 / -3.1	6.5	C 9 H 16 79Br N 10 O 7
+3.1 / +1.4	9.5	C 19 H 20 79Br O 8
-7.5 / -3.4	21.0	C 23 H 9 N 3 O 8
+1.4 / +0.7	6.5	C 12 H 18 81Br N 6 O 8
-9.8 / -4.5	6.0	C 11 H 18 79Br N 7 O 8
+17.8 / +8.1	6.5	C 10 H 16 79Br N 8 O 8
-10.4 / -4.8	20.5	C 25 H 11 O 9
+17.2 / +7.8	21.0	C 24 H 9 N O 9
-1.5 / -0.7	6.0	C 14 H 20 81Br N 3 O 9
-12.8 / -5.8	5.5	C 13 H 20 79Br N 4 O 9
+14.9 / +6.8	6.0	C 12 H 18 79Br N 5 O 9
+4.3 / +2.0	17.5	C 16 H 7 N 8 O 9
-14.4 / -6.6	2.5	C 6 H 18 81Br N 10 O 9
-4.5 / -2.0	5.5	C 16 H 22 81Br O 10
-15.7 / -7.2	5.0	C 15 H 22 79Br N O 10
+11.9 / +5.4	5.5	C 14 H 20 79Br N 2 O 10
+1.3 / +0.6	17.0	C 18 H 9 N 5 O 10
-17.4 / -7.9	2.0	C 8 H 20 81Br N 7 O 10
+10.3 / +4.7	2.5	C 7 H 18 81Br N 8 O 10
-1.0 / -0.4	2.0	C 6 H 18 79Br N 9 O 10

[Theoretical Ion Distribution] Page: 1
Molecular Formula : C20 H17 N4 O4
(m/z 377.1250, MW 377.3794, U.S. 14.5)
Base Peak : 377.1250, Averaged MW : 377.3779(a), 377.3786(w)

m/z	INT.	
377.1250	100.0000	**
378.1280	23.8664	**************
379.1305	3.5235	**
380.1330	0.3884	
381.1355	0.0344	
382.1379	0.0025	
383.1404	0.0002	

Figure S39. The HRFABMS data of **6**

Parameters for calculating specific rotations
Functional: B3-LYP
Basis set: def-SVP
Gridsize: m3
Frequency: 0.7735713524617996E-01
Frequency / eV: 2.104995653760073
Frequency / nm: 589.0000000000000
Frequency / cm^ (-1): 16977.92869016530

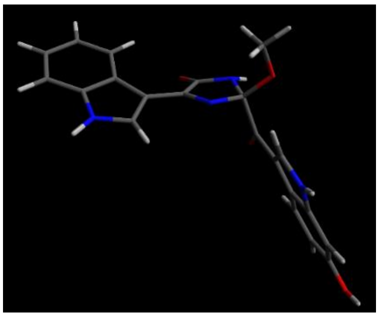
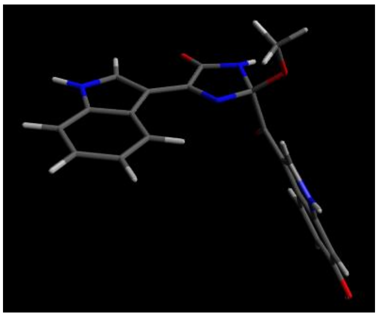
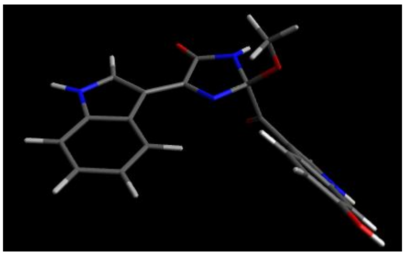
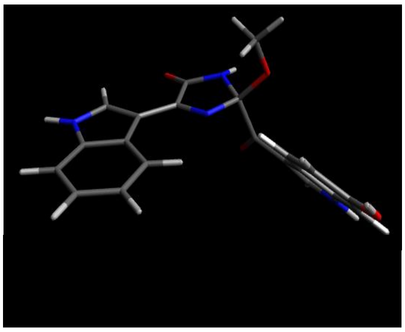
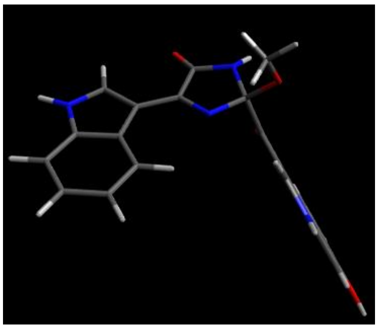
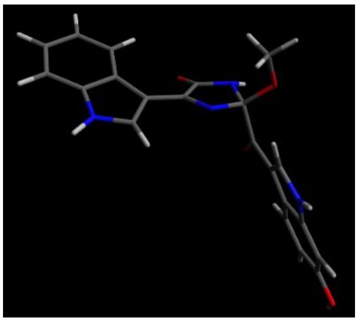

Major conformers (> 2% population) under the relative energy of 10 kJ/mol in calculated Boltzmann distribution.

Compound **1**	1/3*trace (dipole polarizability)	1/3*trace (rotatory dispersion)	Specific rotation [α] in deg*[dm(g/cc)]^(-1)
26.305 %	297.9	4.4	+ 440.4
23.069 %	298.6	5.5	+ 545.0
11.573 %	292.6	-9.4	-932.6
9.939 %	292.6	-10.4	-1033.8
5.847 %	306.8	-0.9	-90.9
5.141 %	292.6	-10.4	-1033.8

Figure S40. Calculated optical rotations of major conformers of **1.**

Parameters for calculating specific rotations
Functional: B3-LYP
Basis set: def-SVP
Gridsize: m3
Frequency: 0.7735713524617996E-01
Frequency / eV: 2.104995653760073
Frequency / nm: 589.0000000000000
Frequency / cm^ (-1): 16977.92869016530

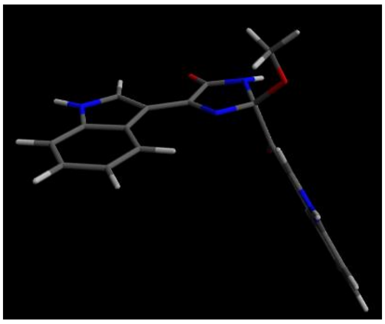
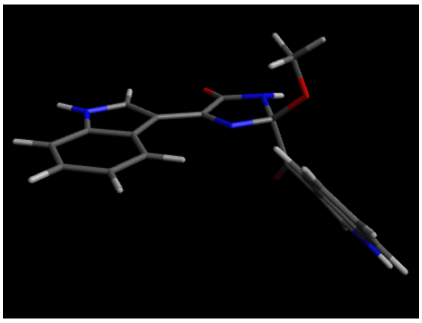
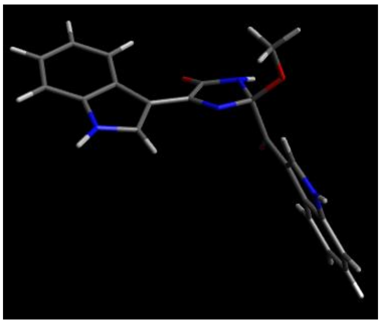
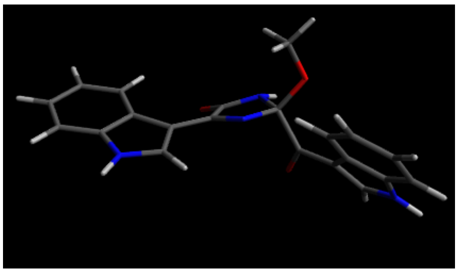
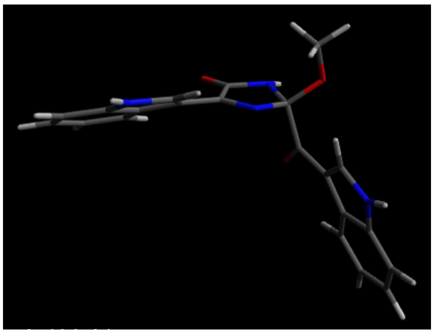
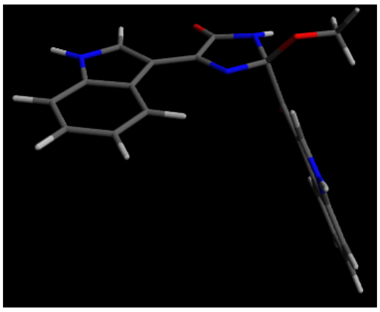

Major conformers (> 2% population) under the relative energy of 10 kJ/mol in calculated Boltzmann distribution.

Compound **2**	1/3*trace (dipole polarizability)	1/3*trace (rotatory dispersion)	Specific rotation [α] in deg*[dm(g/cc)]^(-1)
40.086 %	290.7	4.3	+ 446.6
23.392 %	285.9	-8.6	-893.2
10.293 %	299.5	-0.5	-53.7
4.800 %	295.4	-10.5	-1086.9
3.492 %	299.5	-0.5	-47.9
3.260 %	292.0	5.4	+ 560.4

Figure S41. Calculated optical rotations of major conformers of **2.**

Parameters for calculating specific rotations
Functional: B3-LYP
Basis set: def-SVP
Gridsize: m3
Frequency: 0.7735713524617996E-01
Frequency / eV: 2.104995653760073
Frequency / nm: 589.0000000000000
Frequency / cm^ (-1): 16977.92869016530

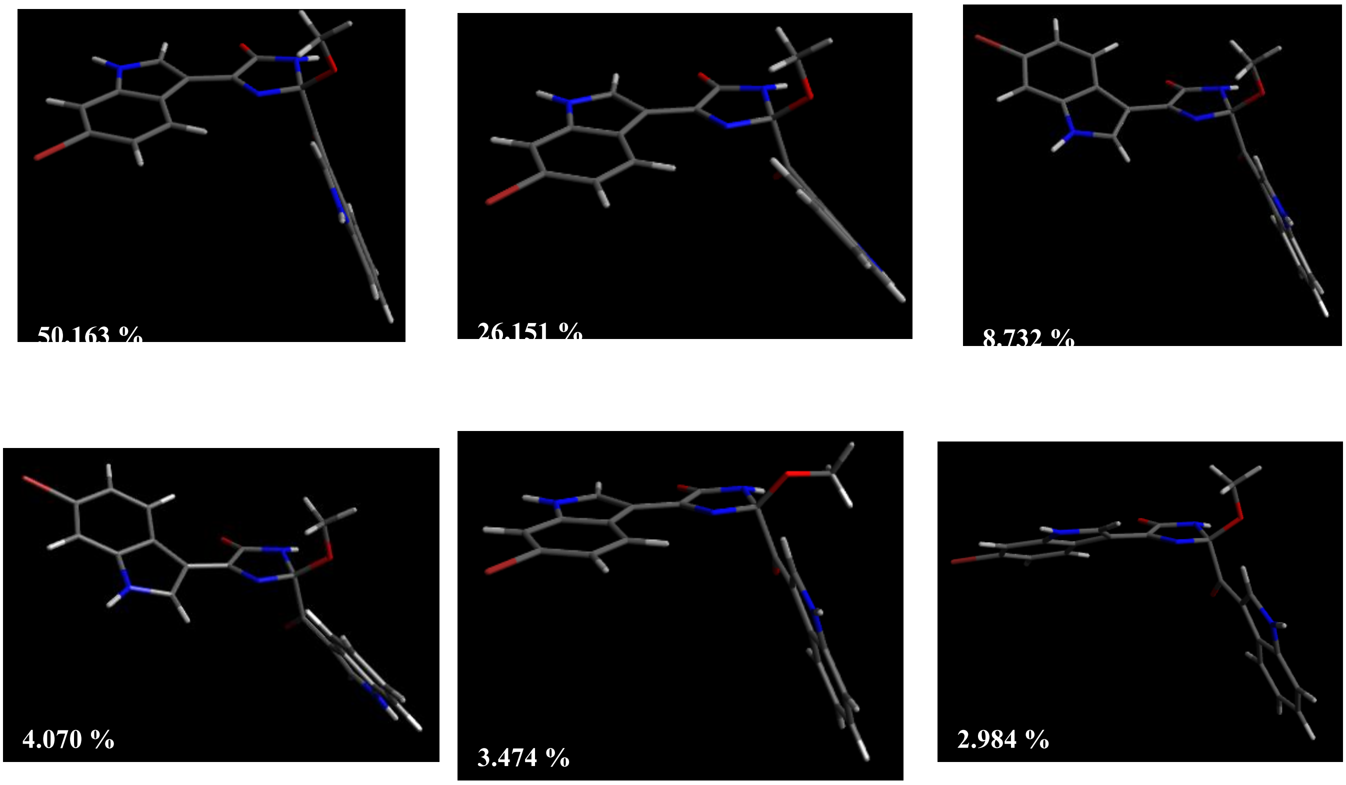

Major conformers (> 2% population) under the relative energy of 10 kJ/mol in calculated Boltzmann distribution.

Compound **3**	1/3*trace (dipole polarizability)	1/3*trace (rotatory dispersion)	Specific rotation [α] in deg*[dm(g/cc)]^(-1)
50.163 %	314.1	7.5	+ 646.6
26.151 %	308.5	-8.1	- 691.4
8.732 %	324.4	0.3	+ 33.0
4.070 %	322.6	-13.7	- 1176.2
3.474 %	315.1	8.6	+ 738.7
2.984 %	324.5	1.1	+ 93.5

Figure S42. Calculated optical rotations of major conformers of **3.**

Parameters for calculating specific rotations
Functional: B3-LYP
Basis set: def-SVP
Gridsize: m3
Frequency: 0.7735713524617996E-01
Frequency / eV: 2.104995653760073
Frequency / nm: 589.0000000000000
Frequency / cm^ (-1): 16977.92869016530

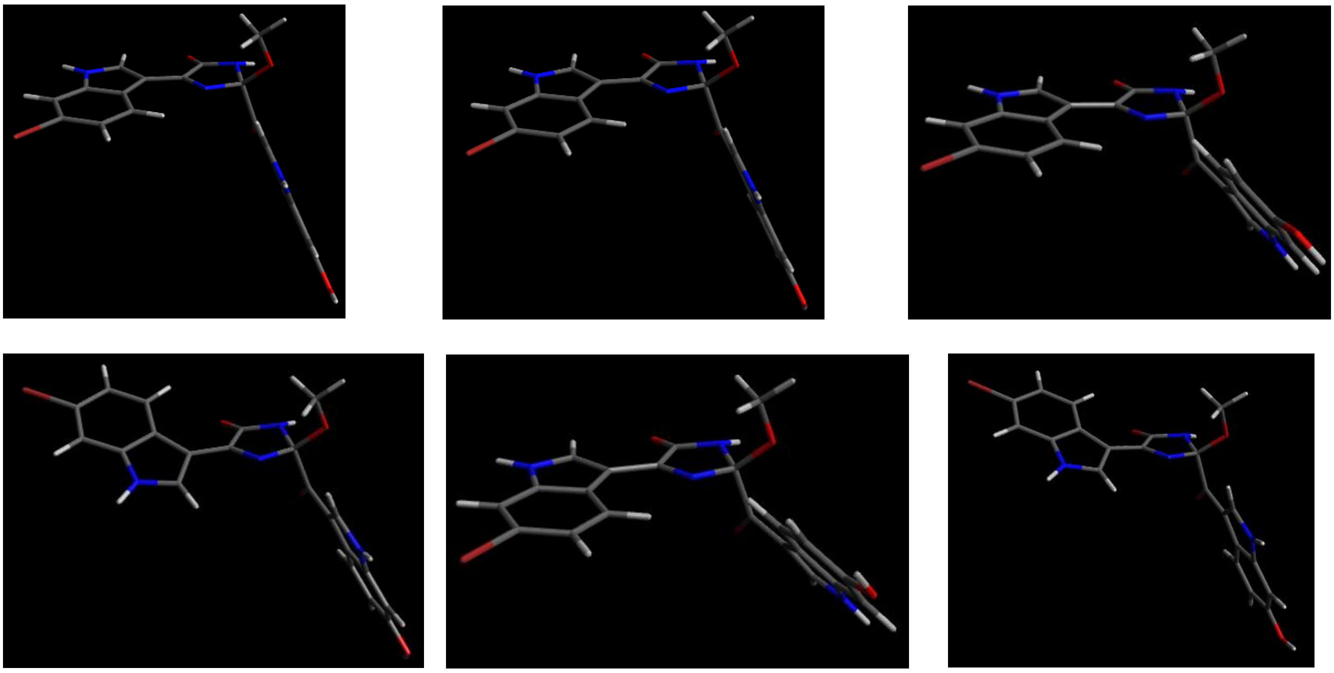

Major conformers (> 2% population) under the relative energy of 10 kJ/mol in calculated Boltzmann distribution.

Compound **4**	1/3*trace (dipole polarizability)	1/3*trace (rotatory dispersion)	Specific rotation [α] in deg*[dm(g/cc)]^(-1)
27.577 %	321.9	3.5	- 287.2
24.151 %	322.8	3.9	+ 327.2
12.285 %	318.2	-12.8	- 1064.0
10.592 %	318.3	-13.3	- 1103.5
4.826 %	333.0	-4.6	-344.3
4.222 %	333.9	-4.0	-330.6

Figure S43. Calculated optical rotations of major conformers of **4.**
